# A System-Level Review of Bio-Inspired Technologies for Next-Generation UAVs: From Aerodynamics to Energy Systems

**DOI:** 10.3390/biomimetics11080596

**Published:** 2026-08-20

**Authors:** Gyeongsu Sim, Hojin Jin, Sangyoon Woo, Won-Gyu Bae

**Affiliations:** 1 Department of Electrical Engineering, Soongsil University, Seoul 06978, Republic of Korea; hyundai6013@soongsil.ac.kr (G.S.); wsy04018@soongsil.ac.kr (S.W.); 2 School of Electronic Engineering, Soongsil University, Seoul 06978, Republic of Korea; alexjin9@soongsil.ac.kr

**Keywords:** bio-inspired UAVs, system-level biomimetics, cross-domain synergy, aeroacoustic propeller design, neuromorphic perception and control, multifunctional morphing wings, energy-autonomous UAVs

## Abstract

Despite the rapid proliferation of unmanned aerial vehicles (UAVs) across industrial, agricultural, and scientific domains, their deployment remains constrained by limited endurance, aerodynamic inefficiency, and acoustic emissions, all mediated by a shared onboard energy budget. Existing biomimetic UAV reviews have generally treated aerodynamics, structures, sensing, control, and energy systems as parallel topics rather than as interacting components of a unified aerial architecture. Drawing primarily on literature published between 2015 and June 2026 and identified through searches of Web of Science, Scopus, and Google Scholar, this review addresses this gap by examining bio-inspired technologies across six principal domains: aeroacoustic and passive flow control, aerodynamic efficiency, multifunctional structural composites, neuromorphic sensing and control, ionic energy storage, and energy harvesting. Its principal contribution is a cross-domain synergy analysis identifying five performance couplings and one structural enabling architecture through which these domains interact physically and functionally. Representative examples include serration-based propeller geometries that can simultaneously reduce noise and power demand; morphing wing surfaces that serve as both aerodynamic structures and triboelectric harvesting substrates; and neuromorphic spiking neural networks that have been reported, in specific event-vision inference benchmarks, to reduce inference energy by three to four orders of magnitude relative to embedded graphics processing unit (GPU)-based implementations. Mechanical harvesting outputs nonetheless remain orders of magnitude below propulsion requirements and are thus positioned as supplementary. Four systemic barriers (unquantified mass–energy balance, undocumented durability, aeroelastic co-design gaps, and heterogeneous metrics) are evaluated, and the resulting synthesis indicates that advancing bio-inspired UAVs requires a transition from structural imitation to functional, system-level biomimetics.

## 1. Introduction

### 1.1. UAV Limitations and the Rise of Biomimetics

Unmanned aerial vehicles (UAVs) have become routine tools across precision agriculture, infrastructure inspection, logistics, and environmental monitoring, with platforms ranging from gram-scale micro air vehicles to multi-kilogram fixed-wing endurance systems. Yet the electrically powered small-to-medium configurations that underpin most operational deployments continue to face three interrelated performance constraints. Flight endurance remains one of the most persistent: commercial multirotor systems typically sustain approximately 20–40 min under representative payloads [1,2], and incremental advances in lithium-based battery chemistry have not fundamentally altered this ceiling [2]. Aerodynamic efficiency presents a related limitation, as fixed-geometry propulsion systems incur meaningful drag penalties whenever mission profiles deviate from their design conditions. Acoustic emissions constitute a third constraint of increasing practical and regulatory significance; the blade–wake interactions responsible for rotor noise concurrently impose propulsive losses [3,4], and bio-inspired propeller designs have demonstrated that this coupling can be addressed simultaneously: studies report concurrent noise reductions and efficiency improvements from biologically derived blade geometries [5,6].

These constraints are not independent: each is mediated by the onboard energy budget, which propagates as a binding limit across propulsion, sensing, control, and acoustic performance simultaneously. A UAV platform’s finite energy reserve constrains every subsystem that draws from it: sensor payload, autonomous computation, actuation bandwidth, and mission duration alike. This coupling is the principal reason that bio-inspired energy-storage and energy-harvesting strategies must be examined alongside aeromechanical technologies in any integrated assessment, rather than deferred to a separate programme.

This diversity of platform scales and mission contexts matters for any review of bio-inspired strategies: aerodynamic regimes, energy budgets, and operational requirements differ substantially across this spectrum, and a bio-inspired mechanism effective at micro air vehicle (MAV) scale does not necessarily transfer to multirotor or fixed-wing configurations. Platform relevance is, therefore, considered throughout the review where evidence permits. Biological flyers (insects, bats, and birds) achieve levels of aerodynamic adaptability, energy economy, and acoustic performance that remain difficult to reproduce in engineered systems at comparable scales. In this review, biomimetics is treated not as visual resemblance to biological form, but as the principled abstraction of transferable performance principles for UAV engineering.

### 1.2. Limitations of Existing Biomimetic UAV Reviews

Several review contributions have established a valuable reference base for this field. Geronel and Bueno [7] provide a broad and valuable overview of bio-inspired flapping-wing UAVs, with emphasis on aerodynamic effects, control strategies, design aspects, and selected cooperative-flight applications. Because their review is primarily centred on flapping-wing vehicle design and control, bio-inspired energy-storage and energy-harvesting concepts, as well as platform-level interactions among aerodynamic, structural, sensing, control, and energy subsystems, remain outside its main scope. Several domain-focused reviews provide substantial technical depth and serve as important reference points: Tanaka et al. [8] classify biomimetic drone studies according to operational challenges; Shahid et al. [9] review bio-inspired morphing across aerodynamic and hydrodynamic systems; Harvey et al. [10] examine avian-inspired wing and tail morphing for UAV flight control and stability; and Prisăcariu and Dumitrescu [11] review low-Reynolds-number unsteady aerodynamics, structural–aerodynamic coupling, and biomimetic design strategies for MAVs.

Across these works, a consistent structural pattern emerges: aerodynamics, sensing, control, and energy systems are treated as parallel topics rather than as interacting components of a shared engineering architecture. The functional relationships among subsystems (their mutual performance dependencies, co-optimisation opportunities, and compatibility constraints) remain largely unexamined. This is not a minor omission. In biological flyers, aerodynamic performance, structural compliance, sensory feedback, and metabolic energy management are co-evolved features of an integrated adaptive system; engineering translations that address these features in isolation may capture only part of the performance potential they collectively represent. The novelty of the present review, therefore, lies not in expanding topical coverage alone, but in treating bio-inspired UAV technologies as potentially coupled elements of a shared, energy-constrained aerial system.

### 1.3. Towards System-Level Bio-Inspired UAV Technologies

Existing biomimetic UAV research has largely emphasised isolated morphological analogies, including flapping-wing mechanics, kinematic replication, and structural mimicry of biological forms. In this review, system-level biomimetics is defined as the coordinated engineering integration of bio-inspired functions and technologies across aerodynamic, structural, sensing, control, and energy subsystems, with their mutual dependencies, trade-offs, and synergies evaluated at the vehicle level rather than as isolated component improvements. Under this definition, the physical and functional coupling among these subsystems is treated as a central design consideration rather than an afterthought.

Three representative couplings motivate this perspective. In propulsion and aeroacoustics, rotor noise and propulsive drag share related vortex formation mechanisms; bio-inspired blade geometries (including owl-feather trailing-edge serrations and humpback-whale leading-edge tubercles) may, therefore, yield correlated improvements in noise and energy performance, though the specific trade-offs remain operating-condition dependent and under active investigation. In the structural and energy domains, cyclic deformation associated with flapping or morphing wing surfaces can provide mechanical input to wing-integrated triboelectric harvesters, as demonstrated in lightweight prototype systems [12,13]. However, long-term flight durability, added mass, and integration complexity remain important practical challenges. In the control and power-management domain, neuromorphic event-driven vision and control architectures have been reported to substantially reduce the energy cost of onboard perception and control inference under specific operating conditions [14,15], potentially easing pressure on the shared energy budget that constrains UAV subsystems.

Several bio-inspired energy technologies reviewed in Section 5 and Section 6, including ionic-gradient electrochemical systems and nanofluidic osmotic conversion, remain at early research stages with experimental demonstrations largely confined to the component level and limited validation under UAV-relevant conditions [16,17]. These technologies are included as long-term research directions relevant to endurance and energy-architecture considerations, not as near-term deployable solutions. In particular, nanofluidic osmotic conversion is treated here as a long-term ground-based or maritime charging infrastructure concept, rather than as an immediate onboard UAV power source, because it depends on externally maintained salinity gradients. Recent convergence across enabling technologies has made integrated bio-inspired investigation increasingly feasible: advances in lightweight flexible electronics, neuromorphic event-driven hardware, additive or advanced manufacturing for complex aerodynamic geometries, and artificial intelligence (AI)-assisted flight autonomy have each lowered practical barriers to multi-domain implementation [7,14,15,18]. Together, these developments make it timely to revisit bio-inspired UAV research not as a collection of isolated biological analogies, but as a set of potentially interacting engineering strategies for future energy-constrained aerial systems.

Against this background, this review makes three contributions. First, it consolidates bio-inspired UAV technologies spanning aeromechanical, structural, sensing, control, energy-storage, and energy-harvesting domains within a unified analytical framework. Second, it evaluates each technology against UAV-relevant engineering constraints (including mass budget, endurance implications, integration feasibility, and evidence maturity) rather than aerodynamic performance in isolation. Third, it synthesises functional coupling mechanisms across these domains to identify design principles and research priorities for future energy-efficient UAV architectures.

### 1.4. Scope and Organisation of This Review

The review covers bio-inspired UAV technologies reported primarily from 2015 through June 2026; foundational references beyond this range are included selectively where they establish the physical basis for reviewed technologies. Literature was identified through Web of Science, Scopus, and Google Scholar using domain-specific search terms for each technical area, supplemented by cross-domain queries targeting the functional relationships examined in Section 7. Inclusion required peer-reviewed publication, an explicitly bio-inspired engineering rationale, and quantitative reporting sufficient for comparative assessment. This review is positioned as a narrative critical review and comparative engineering synthesis rather than a formal systematic review or meta-analysis; coverage is prioritised for technologies for which platform-level relevance, quantitative performance evidence, or clear integration implications can be identified, and depth across the six technical domains is accordingly uneven by design.

To improve transparency and reproducibility without retrospectively recasting this narrative review as a systematic review, domain-specific Boolean searches were re-executed during revision in the Web of Science Core Collection (Topic) and Scopus (TITLE-ABS-KEY) as a coverage audit, using 2015–2026 publication-year filters and a 30 June 2026 eligibility cutoff. Google Scholar and citation chaining were retained for supplementary discovery. Peer-reviewed studies were prioritised when they provided quantitative performance, validated mechanistic, or system-integration evidence relevant to UAV transfer. Exact search strings, eligibility criteria, execution dates, result counts, and coverage-audit notes are provided in Supplementary Material S1.

**Figure 1:**
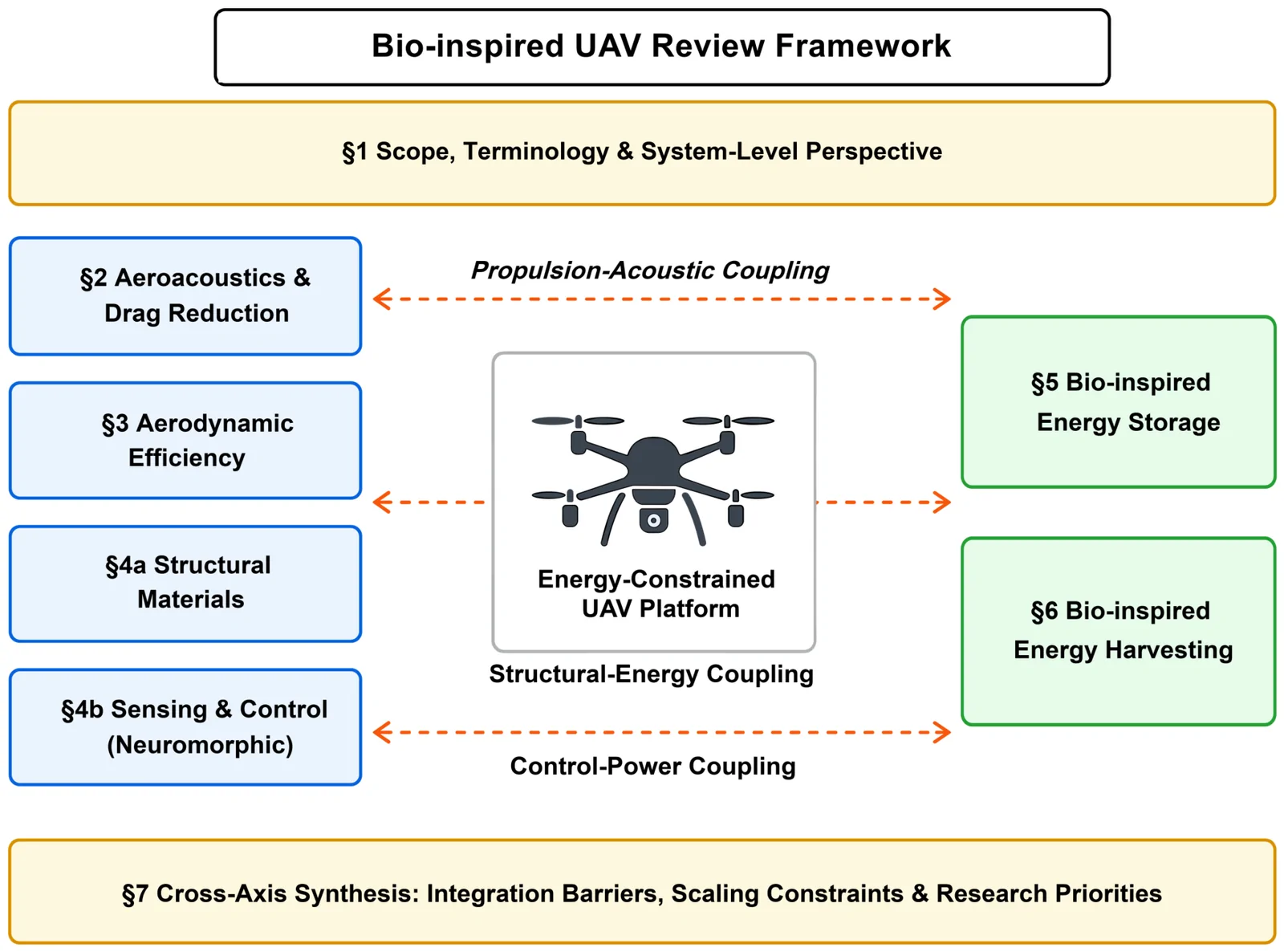
Review framework for bio-inspired unmanned aerial vehicle (UAV) technologies, organised around the conceptual foundation established in Section 1, the principal technical domains covered in Section 2, Section 3, Section 4, Section 5 and Section 6, and the cross-axis synthesis developed in Section 7. Section 1 defines the scope, terminology, and system-level perspective of the review. Section 2, Section 3 and Section 4 cover bio-inspired aeroacoustic, aerodynamic, structural, sensing, and control technologies, whereas Section 5 and Section 6 address bio-inspired energy-storage and energy-harvesting technologies. Representative cross-domain relationships are illustrated around the energy-constrained UAV platform through propulsion–acoustic (C-1), structural–energy (C-2), and control–power (C-4) couplings. Section 7 integrates these domains through analysis of integration barriers, scaling constraints, and research priorities.

Figure 1 presents the review framework as a systems-engineering diagram. Section 1 establishes the scope, terminology, and system-level perspective that frames the review; Section 2, Section 3, Section 4, Section 5 and Section 6 constitute the principal technical domains and Section 7 provides the cross-axis synthesis of their interactions, integration barriers, scaling constraints, and research priorities.

Section 2 reviews aeroacoustic and drag-reduction technologies informed by owl-feather trailing-edge serrations, humpback-whale leading-edge tubercles, and shark-skin riblet surfaces. Section 3 examines bio-inspired aerodynamic efficiency strategies, including avian morphing wings, albatross-inspired dynamic soaring (DS), V-formation energy recovery, and leading-edge vortex exploitation in flapping-wing flight. Section 4 addresses bio-inspired structural materials, sensing systems (including compound-eye optics, lateral-line flow sensing, and neuromorphic event cameras) and autonomous control architectures. Section 5 reviews bio-inspired energy-storage concepts, including ionic-gradient electrochemical systems and nanofluidic osmotic conversion, alongside hydrogen fuel cell configurations as practical high-energy-density comparators against which these emerging concepts and conventional lithium-polymer technology can be evaluated. Section 6 reviews bio-inspired energy harvesting through bio-structured photovoltaics and triboelectric/piezoelectric nanogenerators integrated with flexible wing structures. Section 7 consolidates these findings into a cross-axis synthesis that compares coupling mechanisms, identifies integration barriers, and derives research priorities for more energy-efficient UAV architectures. Section 8 draws the analysis towards a broader discussion of future development directions.

## 2. Bio-Inspired Passive Flow Control: Aeroacoustic and Drag-Reduction Technologies

Passive surface-geometry modification offers a practically attractive approach to improving UAV rotor performance, requiring neither power input nor actuation infrastructure [3]. This section reviews four bio-inspired strategies (owl-feather serrations, multi-organism integrated propeller designs, humpback whale tubercles, and shark dermal denticle riblets) that address aeroacoustic emission or aerodynamic drag through distinct boundary-layer flow-control mechanisms. Although all four modify near-surface flow structure to achieve performance gains, they do so through physically distinct mechanisms: serration approaches act through vortical coherence disruption, tubercles through counter-rotating vortex pair generation, and riblets through quasi-streamwise vortex displacement. The review scope intentionally includes riblets alongside propeller-scale serration approaches; their inclusion is motivated by the frequency with which shark-skin-inspired surfaces are proposed for UAV fuselage and blade applications in the literature, warranting systematic assessment of the evidence base and integration constraints. Table 1 provides a qualitative comparative summary. The biological organisms cited evolved their surface features under selective pressures specific to their own functional contexts (aerial predation, low-speed aquatic manoeuvring, and oceanic cruising) and the functional transfer to UAV hardware involves varying degrees of extrapolation, as noted throughout.

### 2.1. Owl-Feather-Inspired Serration Geometries

Barn owls (*Tyto alba*) and related Strigiformes achieve near-silent flight through complementary feather adaptations. Comb-like serrations on leading-edge (LE) primaries passively control the laminar-turbulent transition on the suction surface, attenuating aeroacoustic source intensity, while a fringe on the trailing-edge (TE) suppresses trailing-edge self-noise [24]. In UAV propeller applications, both mechanisms address the dominant noise sources through passive near-blade flow restructuring, and the engineering translation is direct: serration geometry constitutes a blade edge profile modification manufacturable without significant mass penalty.

A combined computational fluid dynamics (CFD) and experimental study comparing five LE serration morphologies (hook, flaplet, wave, sawtooth, and plate) on a UAV propeller identified the sawtooth configuration as yielding the greatest noise attenuation, with a mean reduction of approximately 2.43 dB and a maximum of 4.18 dB relative to a smooth baseline, accompanied by a 3.53% thrust enhancement under the specific test conditions employed [19]. Field validation on an actual quad-rotor UAV confirmed the sawtooth propeller’s practical noise reduction of 4.73 dB at 5 m hovering height and 3.79 dB at 8 m, representing the most direct UAV-operational evidence in the serration literature [19]. The condition-specific nature of these results is practically significant: the aerodynamic consequence of serration addition depends on the rotor–serration interaction regime and cannot be assumed to extend uniformly across the full operating envelope. CFD-based aeroacoustic predictions are also sensitive to turbulence-model selection and mesh resolution; reported values should, therefore, be interpreted as indicative of comparative ranking rather than as absolute quantitative predictions.

A bio-inspired coupled propeller (BCP) integrating both LE and TE serrations demonstrated concurrent noise reduction of up to 3.1 dB and a 7.9% improvement in the average force-to-speed ratio ($I_{g}$) under laboratory conditions; outdoor hover testing on a quad-rotor UAV confirmed noise attenuation of 3.8 dB at 3 m altitude, 3.3 dB at 6 m, and 2.6 dB at 12 m altitude [5]. It should be noted that BCP power consumption at fixed rotational speed was slightly higher than that of the smooth baseline, although consumption under equivalent thrust conditions was comparable, rendering net endurance implications operating-regime dependent [5]. The causal pathway (LE serrations modifying boundary-layer transition before it reaches the TE, thereby reducing the turbulent boundary-layer intensity that the TE fringe must subsequently attenuate) is physically plausible but has not been confirmed by velocity-field measurements in the rotating frame. That both acoustic and aerodynamic performance can be simultaneously improved by passive blade geometry modification is the most practically significant finding in the serration literature to date; independent replication from other research groups remains limited, with separate investigations of TE serration geometries on UAV propellers from a distinct group reporting up to 4.4 dBA noise reduction through varied serration configurations [25], and this body of evidence should be interpreted as strongly motivated rather than conclusively established.

### 2.2. Multi-Organism Geometric Integration: The 3D-SC Propeller

**Figure 2:**
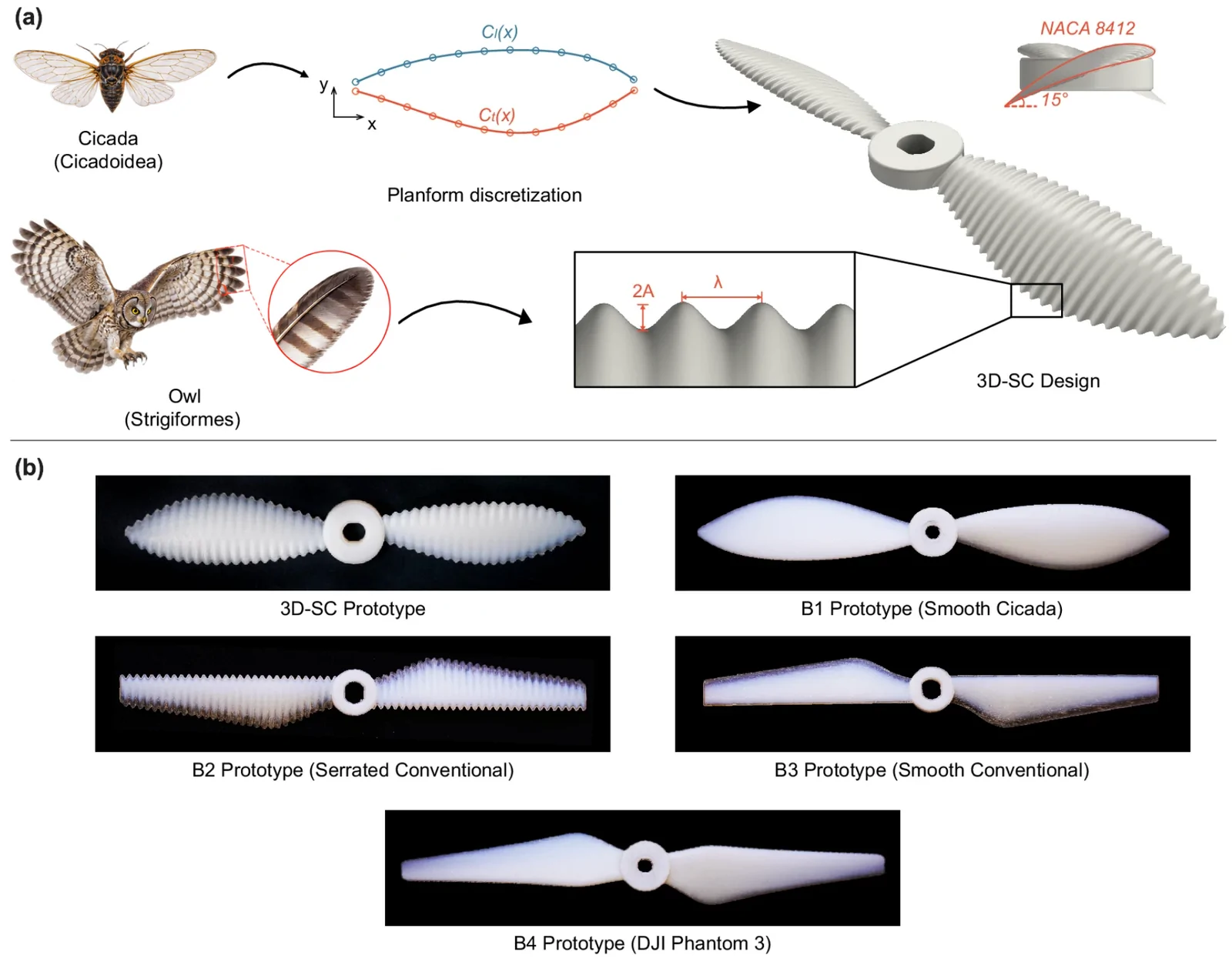
Design concept and prototype implementation of the 3D-SC propeller inspired by owl feather serrations and cicada wing geometry. (**a**) Biomimetic design workflow showing the translation of cicada wing planform characteristics and owl-inspired serrated surface structures into a discretised propeller geometry. The resulting 3D-SC design incorporates sinusoidal serration parameters defined by wavelength (λ) and amplitude (2A). (**b**) Fabricated 3D-SC prototype and benchmark propellers used for experimental comparison, including smooth cicada-inspired, serrated conventional, smooth conventional, and commercial DJI Phantom 3 configurations. It illustrates a representative example of hybrid biomimetic integration in UAV propeller design. Adapted from Wei et al. [6] under the Creative Commons Attribution 4.0 International License; third-party animal imagery in the source figure was omitted and replaced with newly prepared scientific illustrations.

Wei et al. reported a propeller combining sinusoidal LE serration geometry derived from owl feathers with the planform geometry of cicada (Cicadoidea) forewings, designated the 3D-SC (Three-Dimensional Sinusoidal Cicada) propeller [6] (Figure 2). The serration component constitutes direct functional biomimicry, whereas the cicada planform represents bio-derived geometric abstraction: cicada forewings evolved for flapping-wing flight at Reynolds number $Re\approx 2.0\times 10^{3}$, far below the $Re\approx 10^{4}$–$10^{5}$ range of the rotating propeller. The authors cite numerical and empirical evidence suggesting cicada planform aerodynamic benefits extend across a wider Re range, but this transfer has not been independently validated and should be treated as a design assumption rather than a confirmed principle. Benchmarked against four configurations, including a DJI Phantom 3 reference, the 3D-SC prototype demonstrated a maximum reduction of 5.5 dB in unweighted overall sound pressure level (OASPL) at far-field measurement (5 m distance, 30° angle, 50 gf thrust) and a 20.2% reduction in mechanical power consumption relative to the DJI Phantom 3. Three caveats apply: the prototype diameter was constrained to 6 inches by the limited strength of the digital ABS material used in the Polyjet additive manufacturing process, substantially below the 10–18 inch range typical of operational UAVs; the authors confirmed partial scalability in supplementary 12-inch tests, but full advance-ratio characterisation at operational scale was not conducted; and Polyjet manufacturing introduces surface roughness that may confound attribution of performance gains to the bio-inspired features specifically.

### 2.3. Humpback Whale Tubercle-Inspired Leading-Edge Geometries

The rounded leading-edge protuberances on the pectoral flippers of the humpback whale (Megaptera novaeangliae) are associated with exceptional low-speed manoeuvrability. Wind-tunnel experiments on scaled flipper models established that tubercles can yield higher maximum lift coefficients and delay the stall angle by approximately 40% ($\alpha_{\max}$ shifting from ∼12° to ∼16.3°), with the protuberances acting analogously to vortex generators that energise the boundary layer and help maintain attached flow [26]. This biological evidence motivates the engineering concept, but the humpback flipper operates aquatically at $Re\approx 10^{6}$, while UAV propeller blades operate at $Re\approx 10^{4}$–$10^{5}$. Dynamic similarity is, therefore, not guaranteed by geometric scaling alone. Because Reynolds number governs the relative importance of inertial and viscous effects, reducing the operating Reynolds number can alter boundary-layer transition, separation and reattachment, and the resulting aerodynamic loading. A Reynolds-dependent numerical comparison of geometrically similar tubercled airfoils at $Re=1.2\times 10^{5}$ and $1.5\times 10^{6}$ showed that the underlying mechanism involving streamwise and transverse vorticity persisted across both flow regimes, whereas the associated stall and lift characteristics differed substantially [20]. Complementary experimental and numerical evidence at $Re=1.0\times 10^{5}$ further demonstrates that tubercle-induced flow-control effects, including gradual stall behaviour and configuration-dependent aerodynamic benefits, can remain operative in Reynolds-number regimes relevant to small UAVs and MAVs [21]. Accordingly, humpback-whale tubercles should be transferred to UAVs as a mechanism-level design principle requiring Reynolds-specific validation, rather than through direct geometric scaling of whale-scale hydrodynamic performance. The more relevant aeronautical evidence comes primarily from wind-turbine-blade studies.

**Figure 3:**
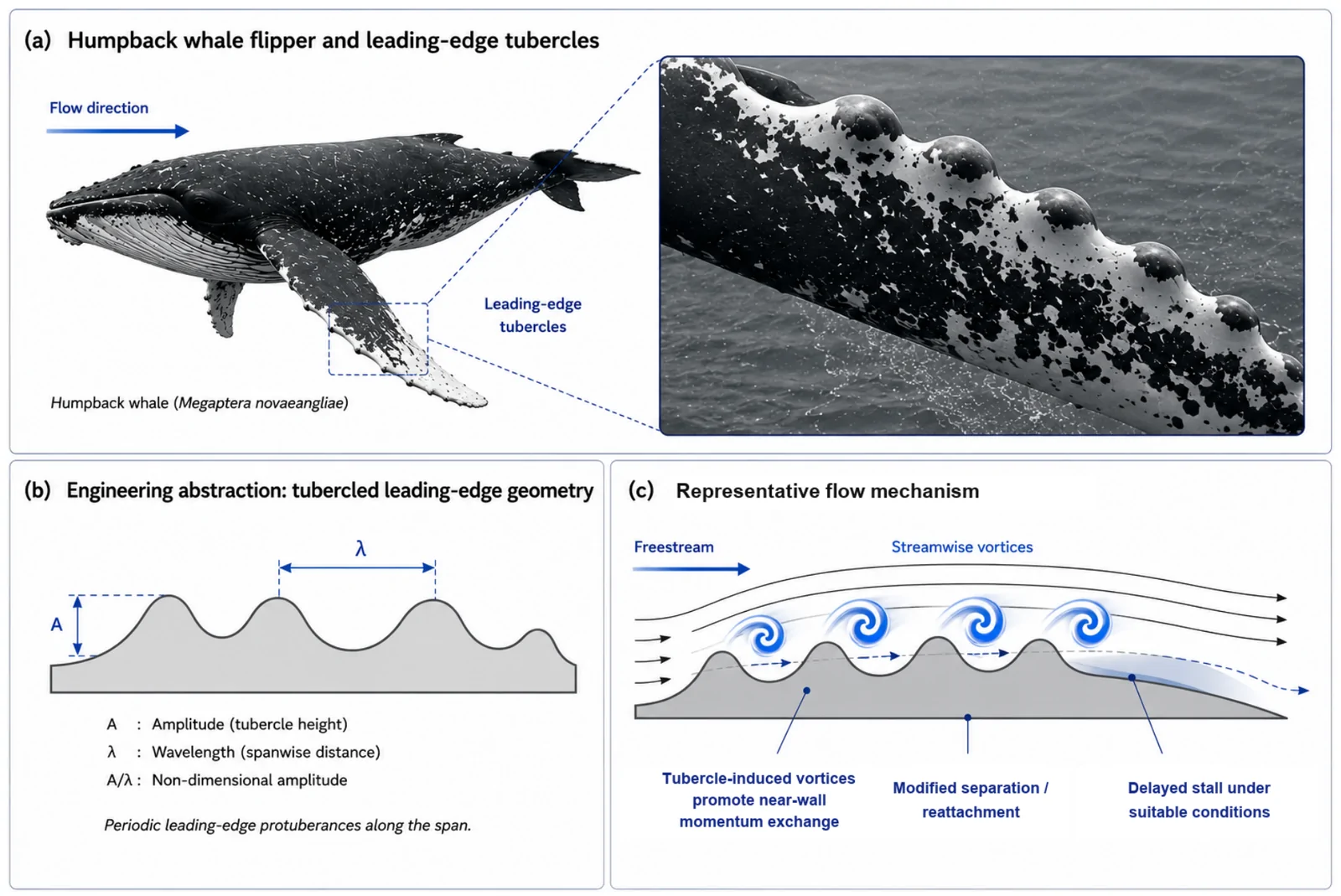
Biological morphology, engineering abstraction, and representative flow mechanism of humpback-whale-inspired leading-edge tubercles. (**a**) Humpback whale pectoral flipper and enlarged view of the leading-edge tubercle morphology. (**b**) Engineering abstraction of periodic leading-edge protuberances characterised by amplitude *A*, wavelength λ, and the non-dimensional amplitude A/λ. (**c**) Schematic representation of the associated flow mechanism, illustrating tubercle-induced vortical structures and their potential role in promoting near-wall momentum exchange, modifying boundary-layer separation and reattachment, and delaying stall under suitable operating conditions. Schematic prepared by the authors based on the mechanisms and numerical and experimental evidence reported in Rostamzadeh et al. and Ladha et al. [20,21].

Figure 3 summarises the biological morphology, engineering abstraction, and representative flow mechanism underlying humpback-whale-inspired leading-edge tubercles. The Reynolds-number constraints governing their transfer to UAV applications are addressed separately in the preceding discussion and further synthesised in Section 7.8.

A numerical parametric study of tubercle amplitude and wavelength on a horizontal-axis wind turbine blade found that certain configurations could reduce reverse-flow separation regions, while also demonstrating that the aerodynamic effect is governed by the amplitude-to-wavelength ratio (A/λ) and the spanwise tubercle coverage [22]. The same numerical study found the aerodynamic effect to be strongly operating-condition dependent: tubercles degraded blade performance at low wind speeds ($V_{\infty }\leq 7\;m/s$) but increased blade torque at higher wind speeds ($V_{\infty }=20$–25m/s), where delayed peak separation and trough-generated vortices suppressing spanwise flow reduced the stall strength. Placing tubercles over the outer 60% of the blade span with a small amplitude-to-wavelength ratio eliminated the root backflow region while maintaining overall blade performance [22].

The critical practical limitation is that the benefit is confined to high-incidence, near- and post-stall conditions: at low wind speeds the tubercles instead generated vortex disturbances and blockage effects that reduced performance without a compensating separation-delay benefit [22]. This operating-regime penalty constitutes a fundamental unresolved trade-off: the crossover angle at which stall-tolerance exceeds the cruise-efficiency cost is geometry- and Reynolds-number dependent and has not been resolved at UAV-relevant scales. For multirotor platforms operating propellers well below stall-critical conditions, this trade-off is particularly unfavourable. The evidence base is most appropriately applied to fixed-wing UAV wing design in turbulent or gusty environments where stall robustness is a primary objective. More broadly, recent flow-control literature shows that separation-control effectiveness depends on local flow conditions and, for actively excited systems, on nondimensional actuation parameters including dimensionless excitation frequency and momentum coefficient, with effective or optimal actuation settings remaining case-dependent on geometry, actuation location, angle of attack, and freestream conditions [27].

### 2.4. Shark Dermal Denticle-Inspired Riblet Surfaces

**Figure 4:**
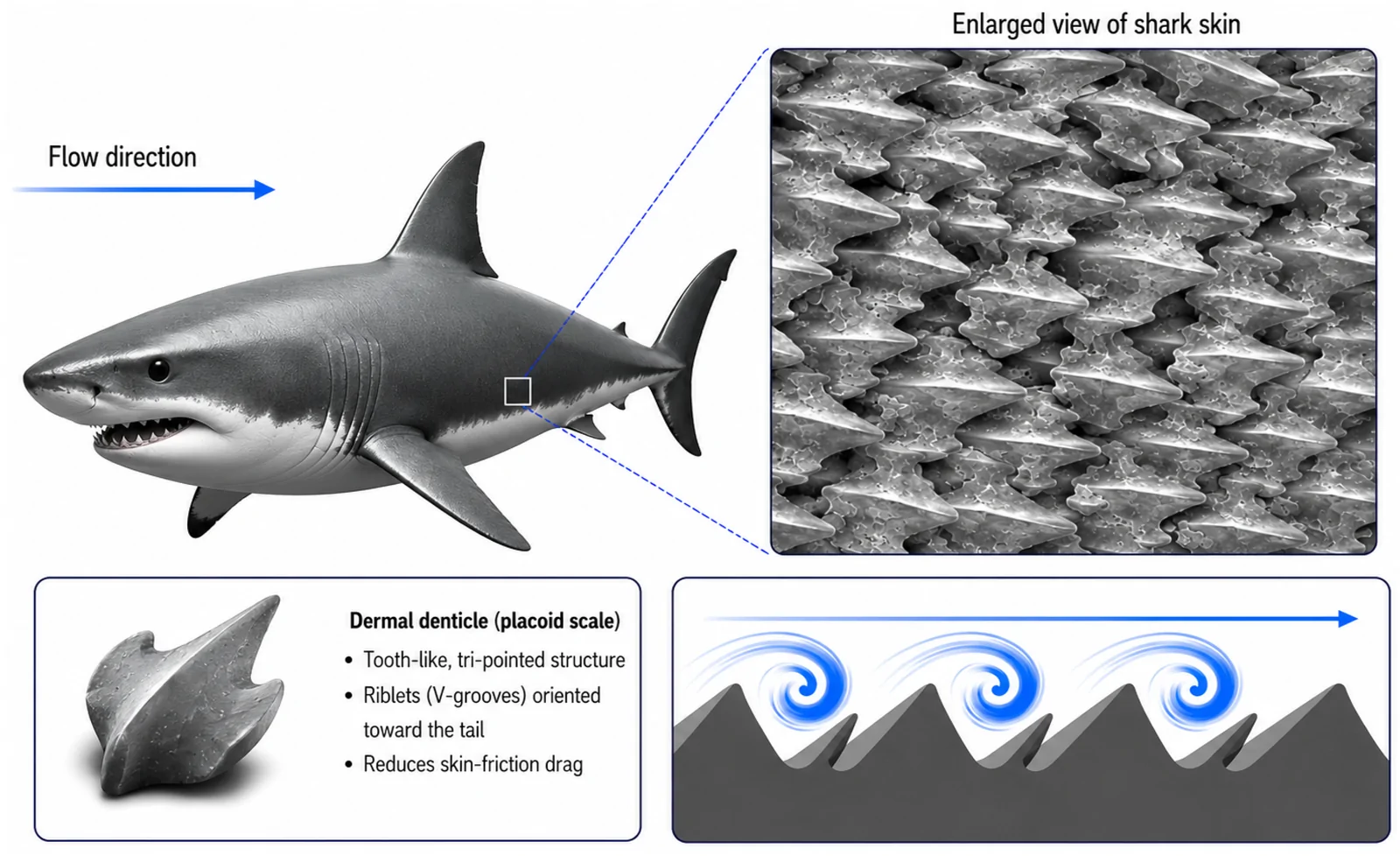
Schematic of shark-skin-inspired riblet structures for passive drag reduction. Shark dermal denticles consist of tooth-like, riblet-forming surface features aligned with the primary flow direction. These longitudinal microstructures suppress near-wall transverse motion and modify the development of streamwise vortical structures, thereby reducing skin-friction drag. It illustrates the biological morphology of dermal denticles and the corresponding engineering interpretation used in biomimetic riblet surface design. Schematic prepared by the authors based on the biological morphology and flow-control mechanisms described in Refs. [23,28,29,30,31,32].

Shark dermal denticles present V-shaped longitudinal microgrooves oriented with the primary flow direction (Figure 4). The drag-reduction mechanism (displacement of quasi-streamwise vortices in the turbulent viscous sublayer, reducing instantaneous wall shear stress) is the most extensively studied passive near-wall flow-control technique in engineering fluid mechanics [28]. The governing parameter is the dimensionless riblet spacing $s^{+}=su_{\tau }/\nu$; maximum drag reduction has been reported at approximately $s^{+}\approx 15$ wall units in flat-plate turbulent boundary-layer studies [29], with the groove cross-sectional area parameter $A_{g}^{+1/2}\approx 10.7$ providing a more geometry-independent optimum predictor across different riblet shapes [29] (the closely related value $A_{g}^{+1/2}\approx 11$ marking the onset of the viscous-regime breakdown beyond which drag reduction deteriorates [29]). Outside the optimal range, net drag increases are consistently reported.

Several limitations substantially constrain UAV relevance. A large-eddy simulation study of a novel biomimetic riblet topology reported up to 21.45% skin-friction drag reduction at Re=40,459 under pipe-flow conditions [23], but large-eddy simulation (LES)-derived values are sensitive to subgrid-scale model selection and near-wall mesh resolution, and no validation on rotating aeronautical hardware exists. Experimental comparison of shark skin specimens with engineered analogues has shown that the biological surface achieves additional drag reduction through passive scale erection in reversing flows (a dynamic reconfiguration without a current engineering equivalent [30]), meaning static riblets capture only a subset of the biological mechanism [31]. Surface wettability modifications of three-dimensionally printed denticle arrays further confirm that geometry alone does not fully replicate shark-skin performance [32].

Three constraints are acute for UAV applications. The optimal $s^{+}$ varies along the blade span with flight speed and revolutions per minute (RPM); a geometry optimised for cruise operates outside the drag-reduction window during hover and transition, potentially incurring net drag penalties across a significant fraction of the mission profile. Microgroove features are susceptible to particulate contamination, as even partial infilling shifts the effective $s^{+}$ below the drag-reduction threshold. Manufacturing tolerance for sub-100 μm groove reproduction and long-term dimensional integrity under aerodynamic loading have not been demonstrated beyond laboratory conditions. Riblets are, therefore, most appropriate for platforms operating in controlled, clean-air, fixed-regime cruise conditions where skin-friction drag constitutes a meaningful fraction of total profile losses: a description applicable to few current UAV mission profiles.

### 2.5. Synthesis: Comparative Assessment and Integration Barriers

The four strategies address aeroacoustic or drag-reduction objectives through physically distinct mechanisms at different spatial scales and flow regimes. Direct quantitative comparison across strategies is not appropriate given the heterogeneous evidence base; studies differ in Reynolds number, fluid medium, validation methodology, and normalisation convention. Table 1 presents a qualitative comparative summary structured to support engineering assessment rather than benchmark ranking. Serration-based strategies hold the strongest case for UAV relevance: evidence derives from rotating propeller configurations at Reynolds numbers characteristic of small UAV rotors, and they are the only strategies for which concurrent acoustic and aerodynamic improvements have been experimentally demonstrated on rotating hardware. Tubercle geometries have a reasonable evidence base for fixed-wing UAV wing applications, but no rotating-hardware validation exists, and the pre-stall drag penalty constitutes a fundamental unresolved trade-off. Riblets possess the most thoroughly characterised physical mechanism but the weakest UAV-specific evidence, with primary data from non-aeronautical flow environments.

Four cross-cutting barriers apply across all strategies. First, systematic in-flight validation across representative mission profiles is absent for all four approaches; the outdoor hover measurements reported in Section 2.1 represent partial field confirmation but do not characterise performance across the full advance-ratio envelope. Second, each strategy exhibits pronounced sensitivity to operating regime, such that benefits demonstrated at one condition are not guaranteed to persist across hover–transition–cruise profiles. Third, manufacturing quality and mechanical durability of bio-inspired surface features under aerodynamic loading and environmental exposure have not been characterised at operational scale. Fourth, system-level mass, aeroelastic, and rotor-balance implications have not been evaluated within UAV endurance budgets. These barriers are not equally severe: serration approaches, operating through flow coherence disruption at length scales substantially larger than typical particulate sizes, may be more tolerant of contamination than riblet surfaces, which require sub-millimetre dimensional precision to maintain their drag-reduction mechanism: an inference that follows from the physical arguments in Section 2.1 and Section 2.4 but awaits experimental confirmation under representative outdoor conditions.

The concurrent acoustic and aerodynamic improvements demonstrated for serration-based designs, consistent with the hypothesis that these objectives share partially overlapping fluid-mechanical contributors at low Reynolds numbers, are examined in the context of integrated UAV endurance in Section 7.1. Engineering targets required for operational deployment across all four strategies define part of the research agenda in Section 7.4.

Biomimetic Classification distinguishes the nature of the biological-to-engineering transfer. Evidence values are indicative within the cited conditions and should not be cross-compared as absolute benchmarks. UAV Evidence Context reports the validation platform of the primary data, not UAV operational conditions.

## 3. Bio-Inspired Aerodynamic Efficiency Strategies

Whereas Section 2 examined passive surface-level adaptations (owl-feather trailing-edge serrations, humpback-whale leading-edge tubercles, and shark-skin riblet geometries) for aeroacoustic noise reduction and drag mitigation, Section 3 shifts focus to active and systemic bio-inspired flight strategies that enhance aerodynamic efficiency. Birds and insects do not rely solely on fixed-geometry wing surfaces; they continuously reconfigure wing planform, exploit atmospheric energy gradients, capitalise on collective aerodynamic interactions, and leverage unsteady vortex dynamics to achieve flight efficiencies that contemporary fixed-geometry UAVs are only beginning to approach. Harvey and Inman [33] provided the first systematic comparison of avian and UAV gliding efficiency, concluding that neither birds nor comparably sized UAVs consistently outperform the other in the supercritical Reynolds number regime, underscoring that biological inspiration does not automatically confer engineering superiority and that quantitative, mechanism-resolved design is required. Nevertheless, biological flyers consistently demonstrate multi-mode performance (combining environmental energy harvesting, social coordination, and unsteady aerodynamics) that no single fixed-geometry UAV replicates simultaneously [10,11,34].

Four strategy classes are reviewed: (Section 3.1) avian morphing wings, which continuously adapt sweep, span, dihedral, camber, and leading-edge geometry to operational conditions; (Section 3.2) albatross-inspired DS, extracting net mechanical energy from atmospheric wind-shear gradients; (Section 3.3) V-formation flight and wake energy recovery, exploiting upwash from conspecifics to reduce induced drag across the formation; and (Section 3.4) leading-edge vortex (LEV) exploitation in flapping-wing micro air vehicles (MAVs), sustaining lift at angles of attack that would stall conventional fixed-wing configurations. Across these strategies, a recurring pattern is evident: morphological adaptability, environmental energy harvesting, social coordination, and unsteady flow control represent mutually reinforcing dimensions of flight efficiency, whose concurrent optimisation motivates the system-level synthesis in Section 7. Table 2 compares morphing degrees of freedom; Table 3 summarises quantified energy benefits for strategies Section 3.2, Section 3.3 and Section 3.4.

### 3.1. Avian-Inspired Morphing Wings for UAV Flight Efficiency

Avian wing morphology provides continuous, multi-axis reconfigurability through the coordinated actuation of shoulder, elbow, wrist, and digit joints. Each articular degree of freedom (DoF) produces a distinct aerodynamic effect: shoulder-driven sweep modulates the aerodynamic centre and static margin; elbow extension governs span and aspect ratio; wrist deflection controls camber distribution and lift-curve slope and digit-based alula deployment delays leading-edge separation at high angles of attack (AoA). Harvey et al. [10] systematically catalogued these DoFs across a broad phylogenetic sample, demonstrating that all major avian lineages exploit some combination of sweep, dihedral, twist, or camber morphing during gliding flight, and that each mode confers measurable aerodynamic benefit at appropriate Reynolds numbers. Harvey and Inman [33] found no definitive evidence that any gliding bird species is either more or less aerodynamically efficient than a comparably sized UAV; however, their survey indicated that species operating in the subcritical Reynolds number regime ($Re≲7\times 10^{4}$) may inspire UAV designs that efficiently extend their operational range into this regime, framing avian multi-axis morphing as a functionally motivated target for UAV design improvement. The absence of in-flight reconfigurability forces fixed-geometry UAVs to operate at off-design conditions for the majority of their mission profile, incurring avoidable drag and lift deficits [10,33].

Variable wing sweep has been reported to reduce drag by approximately 18% when transitioning from the extended (∼20°) to the folded (∼60°) configuration [9]; asymmetric wing-sweep actuation additionally enables roll control without conventional ailerons, as demonstrated by Ajanic et al. [43] in the LisHawk avian-inspired platform, where yaw is produced by a deflectable vertical tail fin. Span extension, inspired by the telescoping feather arrangement of common swifts (*Apus apus*), was estimated to yield approximately 15% fuel saving at constant power for a 40% span increase in the cited configuration [9]; wind-tunnel testing of the biologically inspired Fish Bone Active Camber (FishBAC) compliant structure (whose internal skeletal architecture replicates the fish spine and rib arrangement) demonstrated lift-to-drag ratio (L/D) improvements of 20–25% over conventional hinged flap configurations across UAV-relevant angles of attack [35], with subsequent modular FishBAC wing designs confirming large lift coefficient changes (ΔCL ≈ 0.55) at low drag penalty [36]. Recent University of Southampton work further extended FishBAC morphing to a three-dimensional NACA6409 wing in ground effect at $Re=3.2\times 10^{5}$, showing that aerodynamic performance depends on the chordwise morphing start location and spanwise implementation [44]. Complementarily, Pabon et al. experimentally investigated a distinct Fishbone Skin-Actuated-Camber (SAC) wing at $Re=2.7\times 10^{5}$ and $4.05\times 10^{5}$, demonstrating smooth camber variation without surface buckling and improved aerodynamic efficiency relative to an ideal flap under the higher-Reynolds-number condition [45]. Complementing these morphing-wing studies, recent aerodynamic design work has treated trailing-edge morphing as a flight-condition-dependent optimisation problem using surrogate modelling and CFD across multiple subsonic conditions [46].

**Figure 5:**
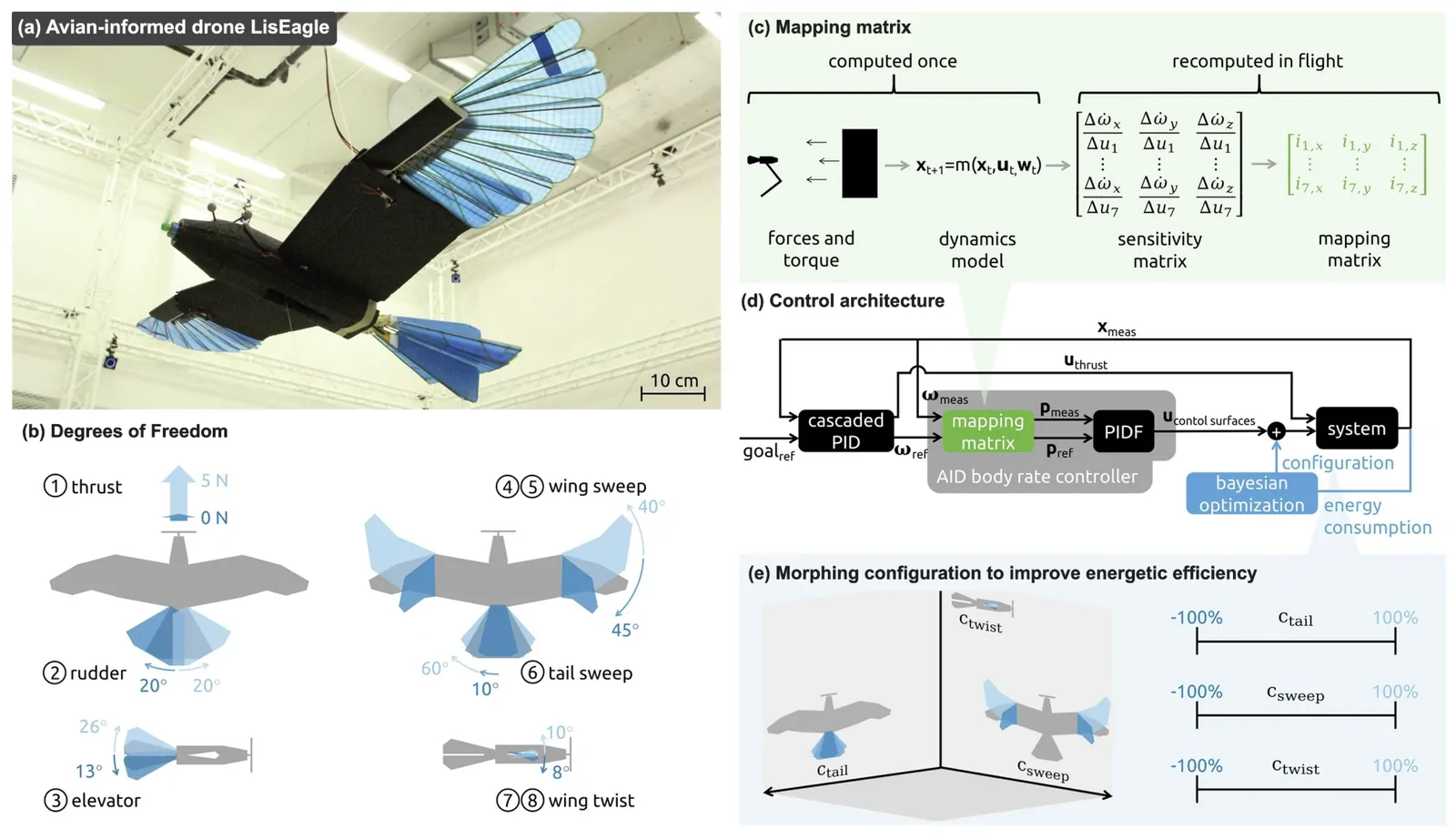
Representative avian-inspired morphing UAV (LisEagle) and its adaptive flight-control framework. (**a**) Prototype of the LisEagle platform. (**b**) Eight controllable degrees of freedom, including thrust, rudder, elevator, wing sweep, tail sweep, and wing twist. (**c**,**d**) Mapping-based control architecture coordinating morphing configurations with flight dynamics. (**e**) Optimisation of wing and tail morphing parameters to improve energetic efficiency during flight. The platform demonstrates how multiple biologically inspired morphing mechanisms can be integrated into a single UAV system. Adapted from Jeger et al. [47].

These kinematic strategies operate on distinct aerodynamic principles from flow-control DoFs: sweep and span alter gross lift distribution, whereas camber, dihedral, and alula devices govern local flow separation and stability. Wing dihedral variation provides lateral–directional stability control and gust-load alleviation, and avian-inspired drones with coordinated wing–tail morphing have been reported to yield energy efficiency gains of up to 11.5% across cruise speeds relative to non-morphing configurations; Jeger et al. [47] attribute these gains to adaptive wing-sweep, wing-twist, and tail-sweep modulation tracking instantaneous aerodynamic demand. Shape memory alloy (SMA)-driven camber morphing (±8°) has been reported to improve L/D by 18% under specific actuation conditions [9], while alula-inspired leading-edge devices reduce stall speed by approximately 20% [9]. PigeonBot [48] represents a well-documented UAV realisation of digit-based morphing, employing real feathers integrated with wrist and finger joints to produce smooth, continuous wing shape reconfiguration. The LisEagle platform and its integrated morphing-control framework are shown in Figure 5.

Principal engineering barriers include the actuator mass penalty (payload fraction reduced by approximately 6 percentage points, from 24% to 18%, owing to morphing-mechanism mass [9]), flexible skin durability under repeated large-deflection cycling, control complexity arising from multi-DoF coupling, and manufacturing scalability of compliant mechanisms [9,10]. Notably, the cyclic deformation of morphing wing surfaces constitutes a candidate substrate for triboelectric nanogenerator integration: a cross-domain coupling mechanism examined in Section 7.

Reported values are not directly comparable across studies, as they were obtained under different Reynolds numbers, geometries, actuation mechanisms, test conditions, and performance metrics.

### 3.2. Albatross-Inspired DS for Energy-Neutral UAV Flight

DS extracts kinetic energy from vertical wind-shear layers in which horizontal wind speed increases with altitude, such that net mechanical energy gain over a complete trajectory cycle equals or exceeds drag-induced losses, enabling sustained low-power or unpowered flight. The mechanism was formalised by Lord Rayleigh in 1883 and is most visibly exploited by the wandering albatross (*Diomedea exulans*), which sustains transoceanic foraging flights of thousands of kilometres with minimal wing actuation by continuously cycling through the wind-shear boundary layer above the sea surface [38].

**Figure 6:**
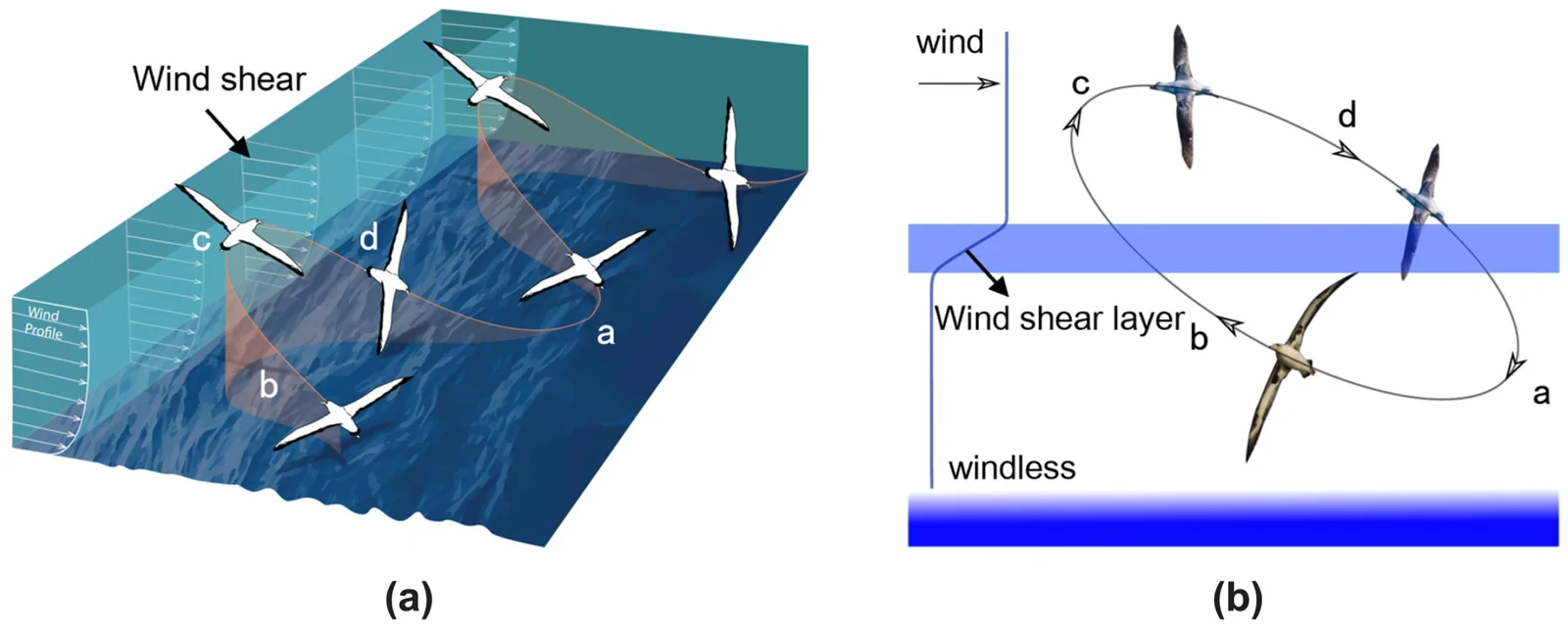
Representative DS trajectories and energy extraction from atmospheric wind gradients. (**a**) Three-dimensional visualisation of a forward DS cycle across a wind-shear layer. (**b**) Circular DS pattern showing the four characteristic phases of the Rayleigh cycle: low-altitude turn, windward climb, high-altitude turn, and leeward descent. Labels a–d denote the low-altitude turn, windward climb, high-altitude turn, and leeward descent, respectively. Repeated transitions across regions of differing wind velocity enable soaring flyers to harvest environmental energy and reduce propulsion requirements. Adapted from Zhang et al. [37].

The canonical Rayleigh cycle comprises four sequential phases [37,38,49]: (i) a low-altitude turn towards the wind near the sea surface, where wind speed is minimal; (ii) a windward climb across the shear layer, gaining airspeed from the increasing relative wind velocity; (iii) a high-altitude turn to a leeward heading at the top of the shear layer, where wind speed is maximal; and (iv) a leeward descent back through the shear layer, completing the cycle with net forward displacement. Energy neutrality over a complete cycle requires the work done by the wind-velocity gradient on the vehicle to equal or exceed the total drag losses integrated along the trajectory [38], a condition met by the albatross across thousands of kilometres of open-ocean flight. Mir et al. [38] report a minimum wind speed of approximately 5 m/s for sustained DS under the specific vehicle and wind-shear-profile assumptions considered in their analysis, below which the modelled energy extracted from wind shear is insufficient to offset drag losses. This 5 m/s value is a condition-specific reference wind speed under the assumed shear profile, not a universal minimum wind-shear-gradient threshold. Two common trajectory modes exist: the travelling pattern (series of 180° turns advancing across the wind) and the loiter pattern (closed loop above a fixed position) [37], both governed by the available wind-shear profile and the same cycle-level energy-balance requirement. Representative trajectory geometries are shown in Figure 6.

Zhang et al. [37] demonstrated that dynamic dihedral variation (−50° to −5°) increased total energy gain by 25.43% and reduced the minimum wind-shear gradient by 15.56% relative to fixed −5°, reinforcing the functional coupling between morphing wing technology and atmospheric energy harvesting. Liu et al. [50] further characterised the trajectory-segment contribution to the DS energy budget, providing a physics-based foundation for mission-level planning. Trajectory-optimisation approaches such as smooth free-cycle DS in unspecified shear wind [51] have additionally demonstrated autonomous DS trajectory generation under variable wind-field conditions, directly connecting this bio-inspired flight strategy to the intelligent autonomous systems focus of the present Special Issue.

Nonetheless, three barriers constrain near-term deployment: the inherent instability of optimal DS trajectories demands robust, low-latency feedback control highly sensitive to wind-field estimation errors [38]; the wind-shear gradients required for sustained DS restrict the operational domain primarily to open ocean and ridge environments; and energy-gain scaling to micro-UAV mass regimes remains experimentally unvalidated. Proof-of-concept DS flights with instrumented radio-controlled (RC) gliders and small fixed-wing UAVs in naturally occurring wind-shear environments have nonetheless confirmed the physical feasibility of the energy-extraction mechanism [38], and DS remains the only passive-propulsion flight strategy empirically validated at organism scale in biological systems: its transition to fully autonomous UAV platforms constitutes one of the highest-leverage opportunities in endurance engineering.

### 3.3. V-Formation Flight and Wake Energy Recovery in UAV Formations

V-formation flight exploits the upwash generated outboard and aft of a leading aircraft’s wingtip vortex, enabling trailing members to reduce their effective angle of attack and induced drag. Lissaman and Shollenberger [39] established the theoretical basis using lifting-line theory, predicting a maximum flight range increase of up to 71% for a 25-bird formation relative to solo flight: a range extension, not a direct 71% energy reduction, a distinction of practical significance for UAV endurance calculations. Weimerskirch et al. [40] provided empirical corroboration with trained great white pelicans (*Pelecanus onocrotalus*), recording heart-rate reductions of 11.4–14.5% during V-formation flight relative to solo flight, and total energy savings of 11.4–14.0%, attributable primarily to extended glide intervals enabled by the upwash field rather than to direct aerodynamic power reduction alone (∼1.7–3.4%). Portugal et al. [52] extended the biological evidence base to the Northern Bald Ibis (*Geronticus eremita*), demonstrating with Global Positioning System (GPS) and accelerometer instrumentation that formation members actively maintained positions in the leader’s upwash and synchronised wing-beat phase relative to the leader’s downstroke: a biological analogue of cooperative sensing and control that anticipates the autonomous swarm architectures reviewed in Section 4.

At the biological level, Mirzaeinia et al. [53] analytically estimated, using a horseshoe-vortex drag model, that leader–follower position switching in a 14-member Canada Goose V-formation improves flight range and endurance by a minimum of 44.6% (rising to approximately 161% under the most favourable wingtip spacing) and further demonstrated that periodic leader–follower rotation is required to equalise cumulative energy expenditure across all formation members, since the leading position receives no upwash benefit. The most recent and highest-fidelity quantification was provided by Mulla et al. [41], who conducted Reynolds-averaged Navier–Stokes (RANS) CFD simulations of a three-aircraft Bayraktar TB2-class medium-altitude, long-endurance (MALE) formation across a structured design-of-experiments space spanning lateral offset (Δy/b), vertical offset (Δz/b), cruise speed, and angle of attack. For near-tip inboard spacing (Δy/b ≈−0.075 to −0.0375), trailing aircraft L/D increased by 20.07–25.92% at α = 0°–2° and up to 39.18% at α = 4°, with drag reductions of 9–22%. These results provide platform-specific, quantitative guidance directly applicable to MALE swarm mission planning. The narrow beneficial spacing band (outside which downwash impingement increases rather than decreases drag) and the real-time aerodynamic sensing and inter-platform communication required to maintain optimal inter-vehicle positioning represent the primary implementation challenges, topics addressed in Section 4. Integration of formation aerodynamics with propulsion energy management and mission scheduling at the system level remains an open challenge directly relevant to the energy-autonomous UAV concept in Section 7. It is noted that V-formation energy recovery applies specifically to fixed-wing platforms in cruise flight; multirotor UAV formations cannot exploit wingtip upwash in the same manner, constraining this strategy to fixed-wing swarms.

### 3.4. Leading-Edge Vortex Exploitation in Flapping-Wing MAV/UAV Flight

Conventional steady aerodynamic theory cannot account for the lift coefficients generated during flapping flight at low Reynolds numbers. Ellington et al. [54] first identified the LEV as the primary mechanism: a stably attached vortex forms along the leading edge during the translational stroke, generating suction pressures far exceeding quasi-steady predictions. The LEV is a Reynolds-number-robust mechanism present across all flapping fliers from insects ($Re\approx 10^{2}$–$10^{3}$) to birds and bats ($Re\approx 10^{4}$–$10^{5}$) [34], and dynamic wing morphing intensifies LEV strength, linking Section 3.1’s morphing strategies to the unsteady aerodynamic regime [34].

Prisăcariu and Dumitrescu [11] identify six unsteady force-generation mechanisms in flapping flight: (i) delayed stall: LEV attachment sustains lift at high AoA; (ii) rotational circulation (Kramer effect): wing rotation at stroke reversal generates an additional lift peak; (iii) wake capture: the wing recovers energy from its own shed wake at the beginning of a new stroke; (iv) clap-and-fling: dorsal wing convergence enhances circulation buildup at downstroke initiation; (v) wing flexibility: passive aeroelastic deformation stabilises the LEV, extends the delayed-stall AoA range, and eliminates transient negative lift; and (vi) tandem-wing interaction: phase-tuned hindwing–forewing vortex coupling enables aerodynamic forces up to 4.3 times body weight in dragonfly-type configurations [11]. Chin and Lentink [42] established the non-dimensional framework of Reynolds number (Re), Rossby number (Ro), and advance ratio (J), demonstrating that LEV stabilisation requires Ro ≈ 4 or lower and that insect-scale unsteady mechanisms cannot be directly extrapolated to larger vehicles without accounting for changes in boundary-layer transition, vortex breakdown, and flow coherence.

For MAV engineering, flexible wings outperform rigid counterparts at $Re<10^{4}$, aspect ratio (AR) values of ≈ 3–4 optimise the balance between spanwise flow stabilisation and LEV attachment, and tandem-wing configurations with adjustable phase offer lift coefficient multipliers unavailable to single-wing designs [11,42]. Free-flight analysis of butterflies confirmed that biological flyers sequentially switch between multiple LEV-type mechanisms across successive wingstrokes, selecting different mechanisms depending on flight speed and manoeuvre type [55], a design principle directly applicable to adaptive MAV control. Engineered MAVs exploiting these mechanisms have achieved viable lift generation: compliant-hinge sub-gram flapping platforms have demonstrated lift coefficients ($C_{L}$) up to approximately 1.6, validating LEV-based force generation at this scale [9]. The central scaling barrier is the Reynolds number gap between the biological inspiration regime and operationally relevant UAV scales, which alters LEV spatial coherence, vortex breakdown rate, and Kelvin–Helmholtz instability characteristics [11]. Overcoming the Re scaling barrier (through adaptive kinematics and intermediate-scale MAV demonstrators) constitutes a principal open challenge for the field, with the cross-domain potential of LEV-coupled piezoelectric energy harvesting examined in Section 7. It is emphasised that LEV exploitation is not presented here as a direct solution for conventional fixed-wing UAVs, but as a scale-specific aerodynamic principle relevant to MAVs and bio-inspired flapping platforms, and as a broader lesson in unsteady-flow control applicable to adaptive UAV systems at larger scales.

Reported values are not directly comparable across studies, as they were obtained under different Reynolds numbers, geometries, test conditions, and performance metrics.

### 3.5. Transition to Section 4

The four bio-inspired strategies reviewed share a unifying conclusion: the aerodynamic benefit is inseparable from real-time adaptive control. Morphing wings must continuously adjust multiple DoFs in response to changing airspeed and atmospheric perturbations; DS trajectories are inherently unstable and demand persistent feedback control under spatially variable wind fields [38]; V-formation energy recovery requires sub-metre-level inter-vehicle positioning maintained through active sensing and communication [41,52] and LEV-based MAVs depend on precisely timed kinematic control of multiple wing degrees of freedom [11,42]. This common dependency (on sensing, feedback, and actuation bandwidth) establishes the agenda for Section 4, which addresses the structural, material, and control architectures required to realise the efficiency strategies reviewed here.

## 4. Bio-Inspired Structural Materials, Sensing Systems, and Autonomous Control Architectures

This section focuses on bio-inspired technologies that directly alter the UAV system envelope rather than merely reproducing biological form: load-bearing efficiency, autonomous attachment, distributed sensing, low-latency perception, and energy-aware decision-making. The technologies reviewed are selected on the basis of their capacity to alter mass budget, energy consumption, or operational endurance, criteria that link directly to the cross-domain synergy analysis in Section 7.

### 4.1. Bio-Inspired Structural Materials and Multifunctional Composites

#### 4.1.1. Biological Archetypes for Lightweight Structural Design

The structural architecture of biological organisms provides compelling templates for mass-critical UAV platforms. Avian skeletal systems, characterised by hollow cortical walls and internal trabecular struts, achieve exceptional bending stiffness-to-weight ratios through hierarchical gradient density distributions: a principle potentially transferable to UAV fuselage spars and wing beams. Dragline silk from Nephila edulis has been reported to exhibit a breaking stress of 1.15 ± 0.20 GPa, a tensile breaking strain of 0.39 ± 0.08, and a mass-specific breaking energy of 165 ± 30 kJ kg−1, indicating a distinctive combination of strength, extensibility, and toughness [56]. In the UAV context, these characteristics are most appropriately interpreted as a biomimetic material-design analogue for lightweight, compliant, and energy-dissipating components, rather than as evidence of a directly deployable UAV structural material. Practical translation remains constrained by scalable artificial production and, within the UAV literature reviewed here, by the absence of demonstrated UAV-scale system integration.

Of particular systems-level relevance is gecko-inspired dry adhesion. The hierarchical fibrillar microstructure of gecko setae generates van der Waals adhesion forces sufficient for surface attachment without chemical bonding [57]. Applied to UAV perching mechanisms, this principle enables meaningful endurance gains: hovering a multirotor demands continuous thrust generation, whereas perching on a vertical or inverted surface reduces propulsion-related power consumption to near zero during stationary observation. More recently, Li et al. [58] reported a self-sensing adhesive (SSA) that integrates micro-adhesive pillar arrays with a capacitive pressure sensor, enabling real-time monitoring of contact force and adhesion state. This dual sensing-adhesion functionality is particularly relevant to autonomous UAV perching control, where attachment verification without additional external sensors represents a meaningful reduction in avionics mass and power draw. However, current gecko-inspired adhesives remain sensitive to surface contamination and substrate roughness, and the reliable transition between attachment and detachment under aerodynamic loading has not yet been demonstrated at operationally relevant scales.

#### 4.1.2. Smart Actuator Materials for Morphing Structures

Biological musculoskeletal systems achieve continuous shape reconfiguration through the coordinated action of skeletal elements, compliant tissues, and muscle-driven actuation, a principle that has motivated engineering analogues based on shape-memory alloys (SMAs), piezoelectric transducers such as lead zirconate titanate (PZT) and Macro-Fibre Composite (MFC) actuators, and electroactive polymers (EAPs) [59]. Nickel–titanium SMAs provide relatively large recoverable deformation and high-force actuation, making them attractive for large-amplitude camber or rib reconfiguration; however, their thermally activated response and cooling requirements limit actuation speed and introduce thermal-management and power penalties. MFC and related piezoelectric actuators provide faster, low-strain actuation suitable for trailing-edge vibration control, flutter suppression, and small-amplitude trim adjustment, and can also support sensing functions through the direct piezoelectric effect. EAPs offer larger and more compliant deformation, making them relevant to soft morphing skins and artificial-muscle-inspired concepts, but their practical use remains constrained by high driving-voltage requirements, durability, and integration challenges. Overall, these actuator classes define a trade-off landscape involving achievable strain, force output, response speed, driving voltage, and system complexity, which must be evaluated together with the propulsion and energy-budget constraints discussed in Section 5 and Section 6 [9,59]. Despite substantial progress, most demonstrations remain concentrated at the material, component, or wind-tunnel prototype level, and fully integrated closed-loop implementations on free-flying UAV platforms remain comparatively limited.

#### 4.1.3. Multifunctional Structural Integration

Beyond actuator functionality, a transformative direction in bio-inspired structural design is the concurrent assignment of energy-storage and load-bearing functions to a single composite laminate. Structural battery composites (SBCs) achieve this by integrating carbon-fibre electrodes, structural electrolyte matrices, and glass-fibre separators within a continuous laminate. Nie et al. [60] reviewed structural-battery-integrated UAV concepts for which the surveyed literature reported up to 41% longer hover time relative to conventional battery configurations; cylindrical structural batteries were discussed separately as a route to improved mechanical robustness and extended drone hover time. Accordingly, the 41% figure should be interpreted as a literature-reported projection for structural-battery-integrated UAVs, not as an experimentally validated flight result for a cylindrical structural-battery quadcopter. A complementary study by Ye et al. [61] demonstrated that switching from hot-press to vacuum-assisted resin transfer moulding (VARTM) in carbon-fibre SBC fabrication raised cell energy density from 23.6 Wh/kg to 41.2 Wh/kg (an improvement of 74.6%) while simultaneously reducing charge-transfer resistance by 80%, underscoring the sensitivity of multifunctionality metrics to manufacturing parameters. The principal barrier to aerospace deployment remains the simultaneous optimisation of electrochemical and mechanical performance, as reinforcing the electrode–matrix interface for structural integrity tends to impede ionic transport, and no in-flight structural battery demonstration on a UAV platform has yet been reported in the open literature.

A second multifunctional pathway involves the use of morphing wing skins as substrates for triboelectric and piezoelectric nanogenerators (TENGs/PENGs). Periodic wing deformation induced by gust response or active morphing constitutes a mechanical energy input directly convertible to electrical energy through contact-electrification or strain-induced polarisation. The dual-function potential of morphing wing skins (as aerodynamic surfaces and mechanical energy-harvesting substrates) represents an emerging cross-domain coupling that is examined systematically in Section 7 (Coupling Mechanism C-2).

### 4.2. Bio-Inspired Sensing Systems

#### 4.2.1. Compound-Eye Optics and Wide-Field Vision

Arthropod compound eyes consist of closely arranged ommatidia distributed over a curved visual surface, enabling wide-field visual coverage, rapid response, and large depth of field; selected artificial compound-eye prototypes reviewed by Jing et al. have demonstrated fields of view approaching approximately 160–180° [62]. This distributed architecture favours broad angular sensing and compact optical integration, but typically trades fine spatial resolution for field coverage and motion sensitivity when compared with vertebrate single-aperture eyes. For UAV platforms requiring wide-area situational awareness, particularly obstacle detection and terrain-relative navigation, this trade-off can be advantageous, although current implementations remain shaped by fabrication, optical assembly, and system-integration constraints.

**Figure 7:**
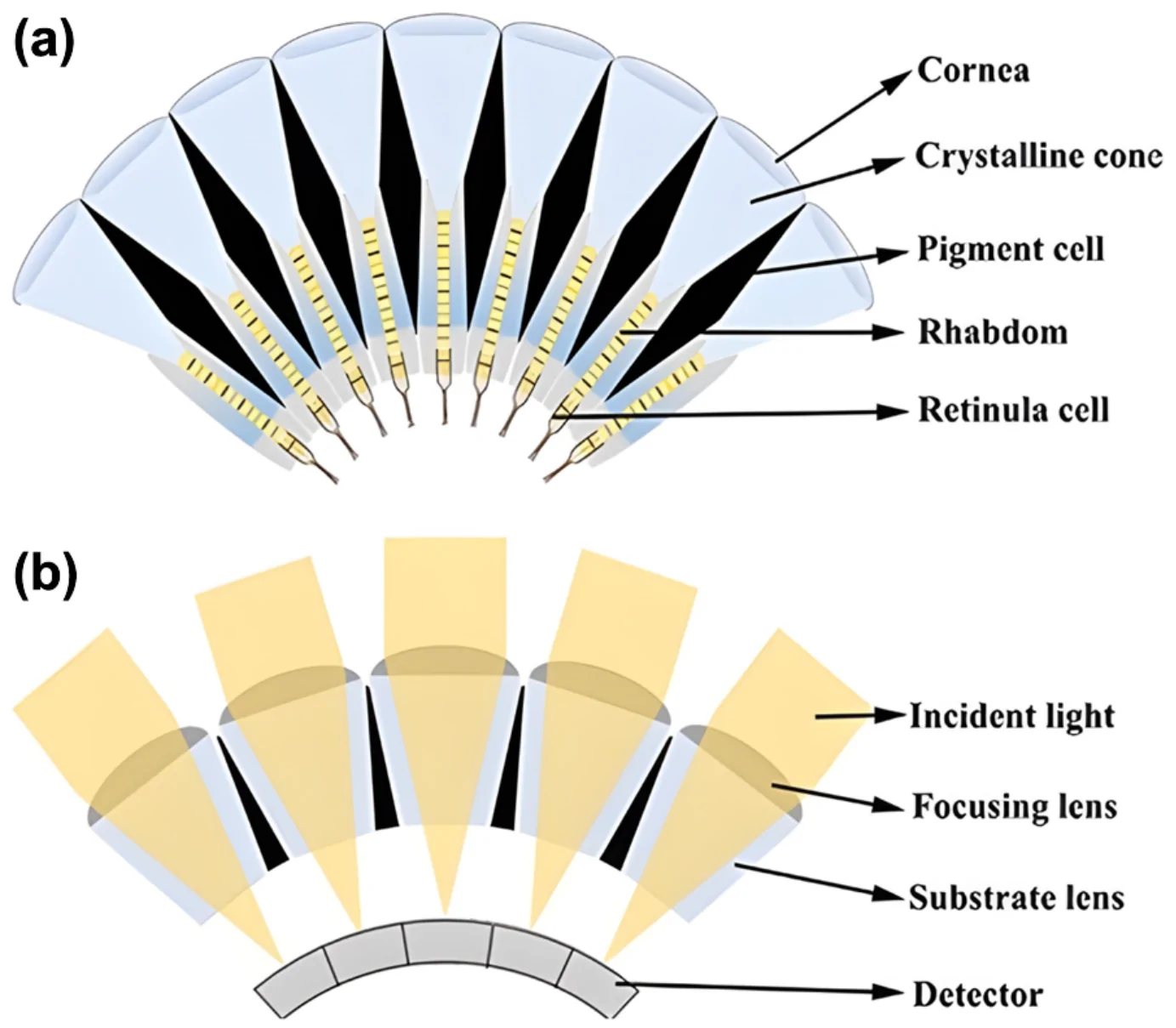
Schematic of biological apposition compound-eye architecture and its engineering translation into an artificial apposition compound-eye imaging system. (**a**) Schematic structure of a natural apposition compound eye, showing the cornea, crystalline cone, pigment cells, rhabdom, and retinula cells as principal ommatidial components. (**b**) Conceptual design of an artificial compound-eye imaging system in which focusing lenses, substrate lenses, and detectors are arranged along a curved geometry to enable parallel light collection and photodetection. Together, the two panels illustrate the biological principles underlying bioinspired compound-eye cameras, including distributed visual sensing, wide field-of-view acquisition, and compact optical integration. Adapted from Jing et al. [62].

Zhang et al. [63] developed an advanced biomimetic multispectral curved compound-eye camera (BM3C) that emulates curved compound-eye geometry by arranging 169 multispectral ommatidia on a curved shell. The prototype achieved a tested maximum field of view of 127.4°, seven simultaneous spectral channels spanning the nominal 500–800 nm range, and a maximum frame rate of 13 fps, with optical distortion below 2%; UAV-mounted airborne experiments demonstrated its feasibility for aerial multispectral imaging and ground-object classification, although further reduction in size and mass remains necessary for broader UAV deployment (Figure 7). A complementary sensing modality exploits the polarisation-sensitive dorsal rim region of insect compound eyes: Liu et al. [64] demonstrated a miniaturised bio-inspired polarisation navigation sensor (weighing 15 g and occupying a 4 × 4 × 2 cm envelope) with indoor and outdoor calibration accuracies of ±0.3° and ±0.5°, respectively. By estimating direction from skylight polarisation patterns, this approach supports artificial-signal-independent heading sensing for mobile robotic and UAV platforms. Together, these compound-eye-derived sensing modalities broaden UAV environmental awareness by providing wide-field multispectral perception and polarisation-based heading information [62,63,64]. However, their platform readiness differs substantially: miniaturised polarisation sensors are already compatible with stringent mass budgets, whereas current multispectral curved compound-eye prototypes remain relatively large and require further size and mass reduction for broader deployment on small UAVs. In addition, the distributed optical architecture can limit per-channel spatial resolution for high-resolution mapping tasks, and the 13 fps frame rate reported for BM3C may constrain fast obstacle-avoidance scenarios unless combined with algorithmic prediction, sensor fusion, or higher-speed readout.

#### 4.2.2. Lateral-Line and Hair-Sensor-Inspired Flow Sensing

The fish lateral-line system, composed of lines of hundreds to thousands of neuromasts distributed along the body surface, provides a biological model for distributed hydrodynamic flow sensing. In this system, superficial neuromasts primarily detect local flow velocity and direction, whereas canal neuromasts respond to flow acceleration and oscillatory stimuli, offering a basis for artificial lateral-line-inspired flow sensors. Analogously, arthropod trichoid sensory hairs detect local airflow and vibration perturbations relevant to insect flight control, with cricket-inspired hair-cell sensors providing a benchmark for highly sensitive low-speed flow detection. Translation of these principles to fixed-wing and rotary-wing UAVs is commonly framed within the flight-by-feel (FBF) paradigm, in which distributed pressure, strain, and flow sensors provide real-time aerodynamic state awareness and can complement conventional sparse probes and model-based state estimation. Hollenbeck et al. [65] systematically reviewed artificial hair sensors (AHSs) for UAV-relevant FBF applications, covering trichoid-like short hairs, whisker-like long flexible hairs, neuromast-type covered-hair designs, and ultrasensitive microscale or cilia-like sensors. These sensor classes differ in morphology, sensitivity, bandwidth, robustness, and integration requirements, but collectively indicate how distributed bioinspired flow sensing could support local airflow characterisation, gust detection, separation monitoring, and aerodynamic-state estimation on UAV platforms.

#### 4.2.3. Neuromorphic Event Cameras

Conventional frame-based cameras acquire images at fixed frame rates, generating significant redundant data during static scene periods while risking motion blur at high angular velocities, characteristics that impose continuous processing loads on the computing subsystem and are poorly matched to the dynamic, low-latency requirements of autonomous UAV flight; reducing this data burden directly alleviates perception-related power consumption. Biological retinae, by contrast, encode only luminance change events at the pixel level, transmitting sparse, asynchronous spike trains that concentrate information in proportion to scene dynamics. Akanbi and Ayomoh [66] describe event cameras as bio-inspired vision sensors that emulate change-driven visual sensing through asynchronous per-pixel brightness-change detection, offering microsecond-scale temporal resolution, low latency, low power consumption, high pixel bandwidth, and high dynamic range of approximately 140 dB compared with approximately 60 dB for conventional frame cameras. Their systematic review of event-based vision for autonomous UAVs covered peer-reviewed studies from 2015 to 2025 and was structured around datasets, simulation tools, algorithmic paradigms, application areas, and future research directions. In terms of UAV applications, the review synthesised evidence across visual simultaneous localisation and mapping (SLAM) and odometry, obstacle avoidance and collision detection, GPS-denied navigation and terrain-relative flight, infrastructure inspection and anomaly detection, object and human tracking, and high-speed manoeuvring. Across these domains, event cameras were reported to offer operational advantages over frame cameras in high-speed, low-light, and high-dynamic-range scenarios, although practical deployment remains constrained by real-world validation, hardware integration, dataset availability, and standardised evaluation.

### 4.3. Bio-Inspired Autonomous Control Architectures

#### 4.3.1. Swarm Intelligence and Collective Biological Behaviours

From an energy perspective, swarm intelligence reduces mission-level energy expenditure through optimised task allocation, trajectory sharing, and formation management, biological principles with direct engineering relevance. Eusocial insects and schooling fish achieve coordinated collective behaviours (optimal foraging, predator evasion, and distributed task allocation) through local interaction rules that require no centralised coordination, yielding emergent global optimality at minimal per-agent computational cost. Applied to multi-UAV trajectory planning, biologically inspired algorithms (BIAs) formalise analogous principles: Particle Swarm Optimisation (PSO) abstracts the velocity-matching and position-attraction dynamics of bird flocking, while Ant Colony Optimisation (ACO) encodes the pheromone-reinforced shortest-path convergence of foraging ants. Aljalaud et al. [67] provided a systematic review of bio-inspired algorithms for multi-UAV path planning, classifying the surveyed literature around eight foundational algorithm families (particle swarm optimisation (PSO), genetic algorithms (GA), grey wolf optimisation (GWO), ant colony optimisation (ACO), pigeon-inspired optimisation (PIO), fruit fly optimisation (FOA), life-cycle swarm optimisation (LSO), and intelligent water drops (IWD)) together with their enhanced and hybrid variants. The review frames multi-UAV path planning as a computationally challenging problem often related to multiple travelling salesman and vehicle-routing formulations, and shows that bio-inspired heuristics are widely used because of their ability to handle large, constrained, and uncertain search spaces. Rather than establishing a universal superiority of any single method, the surveyed studies indicate problem-dependent trade-offs among solution quality, convergence speed, coordination architecture, online or offline planning mode, and adaptability to dynamic environments. A comprehensive treatment of trajectory-design paradigms for autonomous UAV swarms (encompassing traditional algorithms, bio-inspired algorithms (BIAs), and modern AI-based approaches across 250 reviewed research articles) is provided in [68]. From an energy-aware systems perspective, mission-level energy expenditure can be reduced when formation geometry, inter-UAV coordination, task allocation, and total flight distance are jointly considered. Swarm-intelligence and hybrid BIA–AI frameworks provide computational routes for addressing these coupled objectives, linking trajectory planning, coordination, and energy-budget considerations in the cross-domain analysis developed in Section 7.

#### 4.3.2. Neuromorphic Computing and Spiking Neural Networks

The mammalian central nervous system processes sensory information through networks of spiking neurons whose sparse, asynchronous, event-driven activation achieves complex perception and motor control at energy expenditures of approximately 20 W for the entire human brain, far below conventional von Neumann processors despite performing complex perception and motor functions. Spiking Neural Networks (SNNs) emulate this principle by propagating information exclusively through binary spike events, replacing floating-point multiply-accumulate operations with integer additions and exploiting temporal sparsity to reduce active switching. Paredes-Vallés et al. [14] demonstrated one of the first fully neuromorphic vision-to-control pipelines for a freely flying UAV, deploying an SNN on Intel’s Loihi neuromorphic processor in the two-chip Kapoho Bay form factor interfaced with an event-based camera. The on-board pipeline operated at 200 Hz and was reported to spend 27 μJ per network inference. Real-world flight tests demonstrated hovering, lateral manoeuvring, and optical-flow-divergence landing with the neuromorphic vision-to-control pipeline, while additional robustness tests under varying illumination conditions used the neuromorphic vision-based state estimator combined with a hand-tuned proportional-integral (PI) controller [14]. In a separate offline benchmark, performed on a Nahuku evaluation board containing 32 Loihi chips because the Kapoho Bay configuration does not support energy probing, the Loihi implementation achieved execution frequencies of 274–1637 Inf/s and energy costs of approximately 7.2–27.2 μJ per inference depending on input event density. By comparison, the NVIDIA Jetson Nano platform achieved 14 Inf/s in 5 W mode and 25–26 Inf/s in 10 W mode, with corresponding energy costs of approximately 85,600–86,200 μJ and 75,200–76,500 μJ per inference, respectively [14]. These offline benchmark figures should be interpreted separately from the 200 Hz streaming in-flight operation, since the benchmark used data already loaded in memory and did not include the input/output (I/O) and preprocessing overheads present during real-world flight. A complementary study by Stroobants et al. [69] demonstrated SNN-based end-to-end attitude estimation and control on a Crazyflie quadrotor, with the network running at 500 Hz on an onboard microcontroller and achieving an attitude-tracking root-mean-square error (RMSE) of 3.03°; its 15% spiking activity level suggests the potential energy-efficiency benefits of sparse neuromorphic implementations for low-level flight control. At the functional decision-making level, Guerrero-Criollo et al. [70] developed a bio-inspired neural architecture for a differential-drive ground robot, integrating short-term memory circuits, winner-take-all competitive networks, neuromodulatory control, and nonlinear oscillation circuits to support obstacle avoidance and exploratory behaviour. Together with fully neuromorphic vision-to-control demonstrations, these studies indicate that sparse, bio-inspired computation could reduce the onboard computational burden of autonomous robots and UAVs, although the extent to which such savings translate into propulsion energy or mission endurance remains a system-level question to be evaluated in Section 7 (Coupling Mechanism C-4). Despite these advances, SNN-based controllers currently face challenges in generalising across diverse flight conditions, and the lack of mature neuromorphic hardware development tools and training frameworks compared to conventional deep learning remains a barrier to broader adoption.

#### 4.3.3. Neural Network-Based Adaptive Flight Control

Although artificial neural network (ANN)- and reinforcement-learning-based controllers do not replicate a specific biological mechanism in the way that SNNs emulate spiking neurons or BIAs emulate foraging and flocking behaviour, they are included here as complementary adaptive-control tools because they target the same functional objective pursued by biological flight control systems: robust, real-time adaptation to disturbances, damage, and uncertainty. Their relevance to energy-aware bio-inspired UAVs, therefore, lies in functional rather than mechanistic biomimicry, particularly when deployed alongside the morphing structures and bio-inspired flight-control strategies reviewed in Section 2, Section 3 and Section 4. Within the flapping-wing UAV literature, Geronel and Bueno [7] reviewed control strategies, including sliding-mode control (SMC), adaptive control, neural-network-assisted control, neural-network-based adaptive SMC, and adaptive sliding-mode control (ASMC) combined with reinforcement learning, with emphasis on robustness to disturbances, uncertainties, and wing-damage scenarios. However, direct evidence that such controllers reduce propulsion energy or extend mission endurance remains limited; their power and endurance implications are, therefore, treated as a system-level consideration in Section 7, while C-4 specifically addresses neuromorphic computation–avionics power-budget compatibility.

### 4.4. Synthesis: Cross-Domain Implications for UAV Energy Systems

The bio-inspired advances surveyed in this section define the aeromechanical, sensory, and computational envelope within which UAV energy systems must operate. Smart actuator materials such as SMAs, PZT-based piezoelectric actuators, and EAPs provide implementation routes for morphing wing geometries associated with the aeroacoustic and aerodynamic-efficiency benefits discussed in Section 2 and Section 3. In parallel, structural battery composites can contribute to endurance improvement by integrating energy-storage and load-bearing functions into the airframe; Nie et al. [60] reported structural-battery concepts for UAVs with up to 41% longer hovering time relative to conventional configurations, although this value should be interpreted as a literature-reported projection rather than as a flight-validated result. Distributed flow-sensing arrays and event-based neuromorphic vision reduce sensing and perception burdens, while Paredes-Vallés et al. [14] reported that a Loihi-based neuromorphic vision pipeline consumed three to four orders of magnitude less inference energy than a Jetson Nano benchmark. Swarm intelligence frameworks optimise mission-level energy through task allocation and formation management. These advances couple across domains: morphing skins serve as TENG energy-harvesting substrates, event cameras couple naturally to SNN processors, and neuromorphic avionics reduce the electrical load on energy storage. Most distributed sensing technologies reviewed in Section 4.2 are treated here primarily as standalone sensing enablers rather than as direct C-1–C-5 coupling mechanisms; event cameras are the main exception because of their direct architectural pairing with neuromorphic processors under C-4. Gecko-inspired perching likewise falls outside C-1–C-5, since its energy benefit arises from reducing propulsive hover power during perched observation rather than from a shared physical mechanism or co-located energy substrate. Table 4, Table 5 and Table 6 consolidate each technology domain, biological archetype, UAV application, key quantitative performance metric, and Section 7 coupling label. Section 5 and Section 6 examine bio-inspired energy storage and harvesting technologies for this operational envelope, while Section 7 formalises six cross-domain mechanisms: five performance couplings (C-1 through C-5) and one structural enabling architecture (E-1). The coupling labels indicate primary system-level interactions rather than equal technological maturity.

## 5. Bio-Inspired Ionic Energy Storage Concepts and Energy-Conversion Pathways for UAV Systems

Energy storage constitutes one of the principal bottlenecks constraining UAV endurance, alongside aerodynamic efficiency, propulsion power-to-weight ratio, and autonomous control overhead. As reviewed in Section 2, Section 3 and Section 4, bio-inspired optimisation of propulsion and sensing can reduce specific power demand. Yet the absolute ceiling on mission duration remains set by onboard electrochemical energy capacity. Section 5 addresses Axis B of this review: bio-inspired ionic energy storage concepts and energy-conversion pathways. The scope encompasses two distinct categories. The first comprises ionic-gradient batteries (devices that store chemical potential energy in ion concentration gradients, directly analogous to the electric organ of the electric eel), which represent genuine energy storage architectures (Section 5.2). The second comprises reverse electrodialysis (RED) and nanofluidic osmotic membranes. These are energy-conversion devices included not as onboard power sources but as bio-inspired ionic energy-conversion architectures relevant to UAV charging infrastructure and maritime deployment (Section 5.3). Section 5.1 characterises the conventional UAV power baseline. Section 5.4 synthesises the findings and bridges to Section 7.

### 5.1. Conventional UAV Energy Storage: Performance Benchmarks and Limitations

Lithium polymer (LiPo) batteries currently dominate as the onboard power source for commercial multirotor UAVs. At the pack level, LiPo cells achieve energy densities of approximately 150–250 Wh/kg [2,73], confining typical mission endurance to 20–40 min under representative operational loads [2]. Several interconnected limitations underlie this constraint. First, the theoretical specific energy of lithium intercalation chemistry is approaching practical packaging limits, leaving little headroom for incremental gains [1]. Second, high C-rate discharge during take-off and hover accelerates electrode degradation through lithium plating and mechanical cracking, precipitating capacity fade over operational lifetimes [1]. Third, sustained high-current discharge generates heat that elevates thermal runaway risk and produces an infrared emission signature during flight, limiting certain surveillance applications [74]. Notably, thermal gradients as small as 3 °C within a cell have been shown to accelerate degradation by up to 300% via positive feedback [75], a finding potentially relevant to UAV battery packs, where asymmetric aerodynamic cooling may produce non-uniform cell temperatures. Near-term improvements to lithium-ion chemistry are unlikely to fully remove the endurance bottleneck [1]. These limitations motivate examination of fundamentally distinct energy storage approaches.

Proton exchange membrane hydrogen fuel cells (PEM-FCs) represent the leading near-term alternative for extended-endurance UAVs. Compressed hydrogen carries a gravimetric energy density of approximately 33 kWh/kg. Practical PEM-FC hybrid systems achieve system-level specific energies of 250–540 Wh/kg [74], enabling substantially longer flight durations than LiPo platforms at equivalent system mass. Huang et al. [76] demonstrated that a 3.5 kW hexacopter equipped with a 9 L/35 MPa hydrogen tank and a 0.5 kWh lithium buffer sustains approximately 62 min of flight, extendable to 70 min with optimised energy management. Shen et al. [74] document a 331-min continuous flight by a compressed-hydrogen drone equipped with a 19 L/35 MPa cylinder: an exceptional result achieved under highly optimised conditions and not representative of typical multirotor operation. Despite these performance advantages, PEM-FC adoption remains constrained by hydrogen storage certification, balance-of-plant mass overhead, and the low power density of PEM stacks, which requires lithium hybridisation for high-power transients [74,76]. Energy management strategies for fuel cell hybrid UAVs are reviewed by Wu et al. [77], who categorise rule-based, optimisation-based, and learning-based approaches. Table 7 summarises the comparative energy characteristics of these and bio-inspired systems.

### 5.2. Ionic-Gradient Energy Storage Inspired by the Electric Organ of the Electric Eel

**Figure 8:**
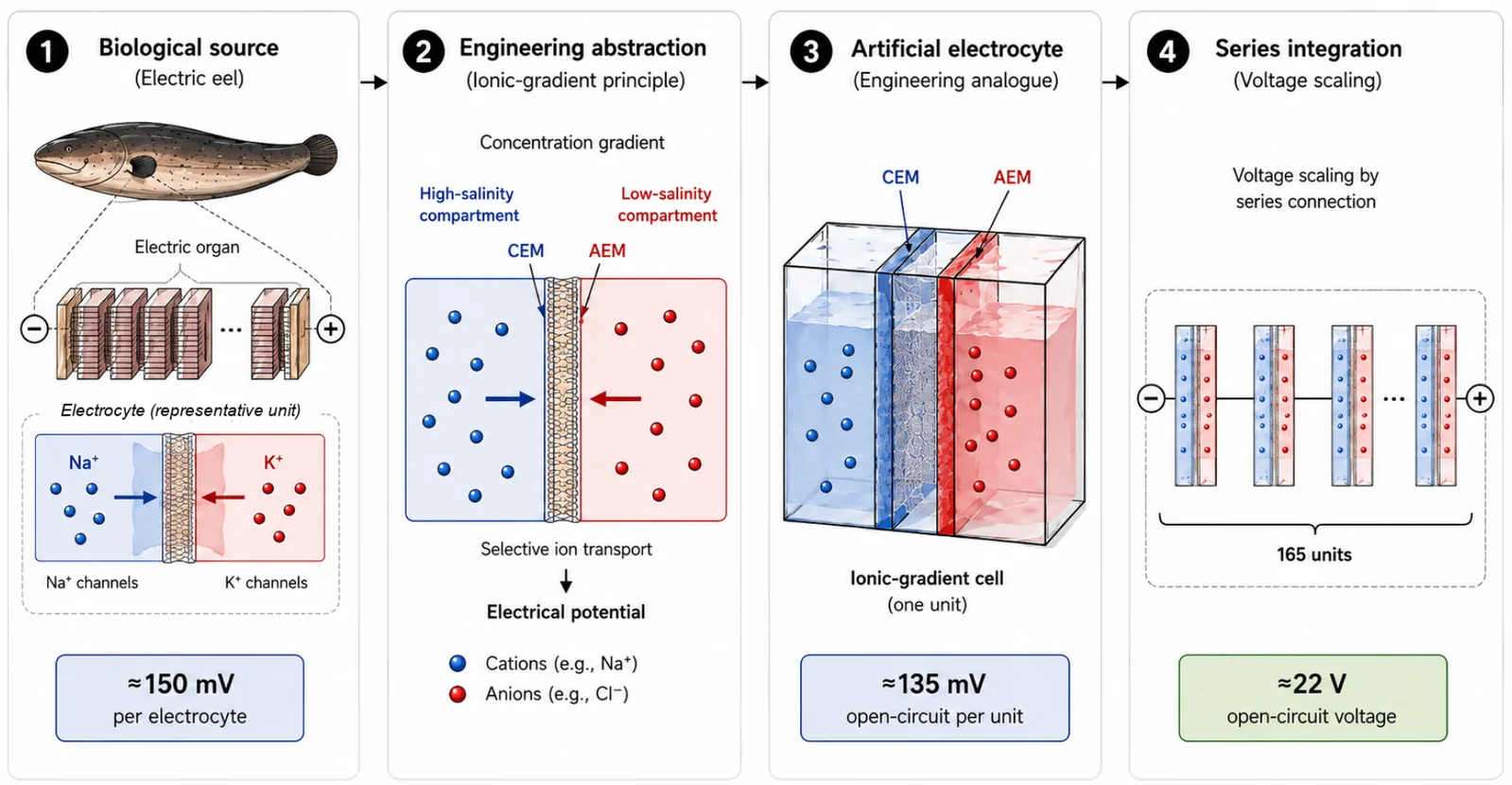
Engineering interpretation of electric-eel-inspired ionic-gradient power architectures. (1) The biological electric eel uses serially arranged electrocytes, each generating approximately 150 mV; the representative electrocyte schematic illustrates the biological ion-gradient principle at a simplified conceptual level. (2) The underlying ionic-gradient principle is abstracted as selective ion transport across concentration compartments. (3) A membrane-based artificial electrocyte produces approximately 135 mV open-circuit voltage per unit. (4) Series integration of 165 artificial electrocytes produces approximately 22 V open-circuit voltage, demonstrating voltage scaling rather than sustained UAV-load power. Schematic prepared by the authors based on the biological and artificial-electrocyte studies cited herein [16,80].

De Santana et al. [82] classified Electrophorus voltai as a distinct species and established the biological benchmark for ionic-gradient energy conversion. E. voltai produces peak discharges of 860 V (among the highest bioelectric discharges recorded in living organisms) through synchronous activation of thousands of series-stacked electrocytes within its electric organ. Each electrocyte exploits voltage-gated Na^+^ and K^+^ channels in its asymmetrically polarised membranes to produce approximately 150 mV per cell [16]. The precise coordination of conduction-velocity differentials across nerve fibres ensures near-synchronous activation of all electrocytes, maximising the summated voltage. This biological synchronisation principle has direct engineering analogues in the contact-activation mechanisms developed by Schroeder et al. [78] and the on-demand thermal gelation mechanism of Zhang et al. [79]. The related species Tetronarce nobiliana (the Atlantic torpedo ray) achieves high-current output through parallel rather than series electrocyte organisation, providing a complementary biological template for systems that prioritise current density over voltage. The fundamental engineering insight is that chemical potential energy stored in ion concentration gradients can be transduced into electrical work via selective membrane permeation, without redox reactions, combustion, or solid-state intercalation. Xiao et al. [16] reviewed this field comprehensively, covering ionic-gradient power sources, triboelectric nanogenerators, and nanochannel membrane applications. The biological architecture and electrocyte operating mechanism are summarised in Figure 8.

The foundational macroscale demonstration was provided by Schroeder et al. [78], who arranged miniature polyacrylamide hydrogel compartments in the electrocyte sequence (high-salinity, cation-selective, low-salinity, anion-selective) repeating in series. Each set of four gel compartments constitutes one tetrameric gel cell. Upon contact activation using a scalable stacking geometry, 2449 individual gel compartments arranged as 612 tetrameric gel cells in series generated 110 V open-circuit with a maximum power density of 27 mW $m^{-2}$ per tetrameric gel cell [78]. A separate Miura-ori thin-film folding approach was also demonstrated as a manufacturable variant. The system is soft, flexible, transparent, and potentially biocompatible, demonstrating that the biological mechanism can be replicated from benign, mechanically compliant materials. However, 27 mW $m^{-2}$ is insufficient to power standard electronic devices. The root cause is the high internal resistance of large hydrogel volumes: limited ionic conductivity combined with long diffusion paths constrains current output even as voltage scales linearly with cell count.

Guha et al. [83] addressed this limitation by reorienting the architecture from eel-inspired (series, high voltage) to torpedo-ray-inspired (parallel, high current) organisation. Using hydrogel-infused paper films of arbitrarily large area configured in both series and parallel, this approach increased maximum power density to approximately 1.80 W $m^{-2}$ per tetrameric unit: a 67-fold improvement over Schroeder 2017 [83]. This demonstrated that the power deficit of the original design is a consequence of geometry and internal resistance, not of the concentration-gradient mechanism itself. The broader materials framework is reviewed by Yang and Suo [84], who establish that hydrogels function as stretchable, transparent ionic conductors enabling hybrid ion–electron circuits with properties inaccessible to conventional solid-state electronics.

The most significant miniaturisation was achieved by Zhang et al. [79]. A microscale soft ionic power source was fabricated from lipid-supported networks of nanolitre agarose pre-gel droplets in the electrocyte arrangement. Before activation, self-assembled lipid bilayers at droplet interfaces prevented premature ion flux, preserving stored chemical energy for more than 24 h. Activation was triggered by transferring the network to lipid-free medium and inducing gelation at 4 °C. For 1.84 nL droplets, the device generated an open-circuit voltage of approximately 86 mV and achieved a power density of approximately 1300 W $m^{-3}$: a volume reduction exceeding $10^{5}$-fold relative to the Schroeder 2017 array [79]. For larger 100 nL droplets, the open-circuit voltage was approximately 127 mV at the cost of substantially lower volumetric power density (∼87.8 W $m^{-3}$), as documented in Extended Data Table 1 of that study. The device was further demonstrated as a biocompatible ionic current source capable of modulating neuronal network activity in three-dimensional neural microtissues, confirming its ionic current character.

The principal limitation of aqueous hydrogel systems (thermal instability and evaporative water loss across UAV operational temperatures) was addressed by Han et al. [80] through fully solid-state artificial electrocytes. Three attributes make this architecture potentially well-suited to UAV deployment: (i) elimination of aqueous electrolyte evaporation under aerodynamic and thermal loads; (ii) mechanical compliance compatible with conformal integration into curved structural surfaces; and (iii) demonstrated operation across both high-altitude cold environments and ground-level tropical conditions. Low- and high-concentration zwitterionic gel films (L-ZIG and H-ZIG) were laminated with anion- and cation-exchange polymer membranes, exploiting a room-temperature ionic liquid medium. Differential scanning calorimetry confirmed no phase transitions from −60 to 150 °C; electrical output was verified stable from −20 to 100 °C [80]. The device generates approximately 135 mV per electrocyte unit. It maintained initial open-circuit voltage when bent to a 2 mm radius and retained greater than 80% voltage over 100 charge–discharge cycles. A 165-unit monolithic array on a polydimethylsiloxane (PDMS) substrate achieved an open-circuit voltage of approximately 22 V. This device is among the most practically engineered ionic-gradient cells reported to date.

Further research has addressed fabrication scalability and storage stability. Xiao et al. [85] introduced the bi-ionic gradient battery (BGB) from exclusively polyelectrolyte hydrogel components, achieving 0.54 V open-circuit voltage and 13 μA ${\mathrm{cm}}^{-2}$ short-circuit current density per unit; origami-based assembly of 56 units yields 22 V [85]. He et al. [86] demonstrated microfluidics-assisted fabrication enabling 801 hydrogel particles within 1.53 min, with serial connection of eight packs yielding 73.33 V and stable performance from 0 to 90 °C; dehydration preserves batteries with full capacity recovery upon rehydration [86]. Wang et al. [81] resolved the storage-stability challenge through an accordion-structured hydrogel battery (ASHB). The accordion geometry suppresses spontaneous inter-compartment ion diffusion during non-working states, extending gradient retention time by at least 30 h relative to simple layer stacking [81].

Notwithstanding these advances, ionic-gradient systems face a critical performance gap with respect to UAV propulsion requirements. Gravimetric energy density in Wh/kg (the metric most directly relevant to UAV endurance) has not been reported for any of these devices; direct gravimetric measurements remain unavailable in the published literature. Sustained propulsion-level energy delivery at UAV-relevant timeframes has not been demonstrated. Current systems also lack characterisation of sustained high-current delivery, mechanical durability under flight-induced vibration, and cycle life under operational conditions. Comparisons with LiPo systems must, therefore, remain qualitative. The absence of gravimetric benchmarks after nearly a decade of development suggests that propulsion-scale deployment remains a distant prospect.

Near-term UAV relevance accordingly lies not in replacing propulsion batteries, but potentially in roles as low-power conformal auxiliary power elements for onboard sensors, embedded power sources for morphing actuators, or structural energy layers within compliant wing skins. None of these applications has yet been demonstrated at UAV-relevant scale. The integration of ionic-gradient films within morphing aerodynamic surfaces remains a long-term design hypothesis grounded in demonstrated mechanical properties, not an engineering claim.

### 5.3. Osmotic Energy Conversion and Nanofluidic Ion-Selective Membranes

RED and nanofluidic osmotic membranes are included here not as onboard energy storage devices but as bio-inspired ionic energy-conversion architectures. They require a continuous external salinity supply, which precludes direct onboard installation. Their UAV relevance lies in ground-based or maritime charging infrastructure exploiting naturally occurring salinity gradients (at coastal river estuaries, inland salt lakes, or from ship-borne deployments) to recharge UAV swarms. This categorisation is maintained throughout the subsection.

RED converts the Gibbs free energy of mixing solutions of differing salinity into electricity via selective ion transport across semipermeable membranes: a mechanism analogous to osmoregulatory processes in fish gills and vertebrate nephron tubules. The theoretical global potential of river-to-ocean salinity gradients is approximately 2.6 TW [87,91]. The foundational framework was established by Logan and Elimelech [95], the technology landscape is reviewed by Yip et al. [87] and Siria et al. [88], and the nanofluidic route is analysed by Zhang et al. [89]. A technology-readiness perspective on RED infrastructure (covering ion-exchange membrane synthesis, fouling mechanisms, and stack design) is provided by Gul et al. [96].

Bio-inspired membrane design has delivered the most significant performance advances. Yao et al. [90] fabricated a UiO-66-NH2 metal-organic framework composite membrane with sub-nanometre (angstrom-scale) window apertures and ∼1.2 nm internal channels that mimic the protein ion channels of the electric eel. Under a 50-fold NaCl gradient, this membrane achieved 1.47 W $m^{-2}$ with long-term saline stability and pH-tuneable surface charge [90]. Wang et al. [91] demonstrated a vermiculite-based heterogeneous nanofluidic membrane with a dual-separation mechanism, achieving 33.76 W $m^{-2}$ under a 500-fold salinity gradient (a 6.2-fold power-density improvement per tenfold increase in salinity gradient) and 25.9 W $m^{-2}$ using natural salt-lake brine [91]. Among 2D nanofluidic platforms, Ti3C2Tx MXene lamellar membranes achieved 21 W $m^{-2}$ (40.6% conversion efficiency), increasing to 54 W $m^{-2}$ with thermal surface-charge regulation [97]; single-layer nanoporous carbon membranes from core-rim polycyclic aromatic hydrocarbons reached 67 W $m^{-2}$: among the highest reported for synthetic RED membranes [92]. The 2D material design space is further surveyed by Xin et al. [17], encompassing graphene, MXene, MoS2, and covalent-organic-framework (COF)-based platforms [17,93], with low-cost nanocellulose alternatives also demonstrating competitive performance [94].

From a UAV systems perspective, the power densities achievable at membrane level are potentially sufficient to sustain trickle charging for lightweight UAV swarms at fixed coastal or estuarine ground stations. Realising this scenario requires engineering continuous salinity supply management, membrane fouling mitigation, electrochemical balance-of-plant, and UAV-side power management, none of which has been addressed in a UAV operational context. No study has yet demonstrated a complete RED-powered UAV charging ecosystem under realistic field conditions. RED systems are accordingly treated as infrastructure-level energy-conversion concepts rather than onboard UAV power technologies.

### 5.4. Synthesis and Pathways to Section 7

The technologies reviewed in this section occupy two fundamentally different roles (Table 7). LiPo batteries and PEM-FC hybrid systems are operationally mature and define the current UAV energy baseline. Ionic-gradient batteries are laboratory-scale concentration-gradient power sources at an early research stage: their voltage and power density are well-characterised, but gravimetric energy density, sustained power delivery, cycle durability, and UAV-environment compatibility remain undemonstrated. RED and nanofluidic osmotic membranes are energy-conversion architectures relevant at the infrastructure level, not the onboard level. Because Wh/kg data are absent for ionic-gradient devices, comparisons with LiPo systems must remain qualitative. This absence reflects the immaturity of the field: current research prioritises proof-of-concept ionic transduction rather than propulsion-scale optimisation, and gravimetric benchmarking will only become meaningful once sustained device-level energy delivery has been demonstrated.

This review’s analytical framework does not assess near-term deployability. Instead, it identifies which physical and functional properties may enable integration paradigms unavailable to conventional energy storage. By this criterion, ionic-gradient batteries occupy a distinctive position. Their mechanical compliance (demonstrated to be retained through bending to 2 mm radii [80] and repeated deformation cycles [86]) is a property not achievable with conventional LiPo or PEM-FC architectures. This compliance raises the possibility of co-locating thin-film power elements within morphing aerodynamic structures reviewed in Section 3 and Section 4. A second distinctive attribute is the ionic (rather than electronic) current character of these devices, which offers potential long-term compatibility with ionic neuromorphic computing substrates. A third attribute relevant to UAV operational robustness is the demonstrated thermal range of solid-state ZIG devices (−20 to 100 °C electrical output; no phase transitions from −60 to 150 °C [80]), substantially exceeding the thermal tolerance of conventional LiPo systems. Each of these integration concepts carries substantial engineering risk and has not been validated at UAV-relevant energy density or scale; all should be treated as long-term research hypotheses rather than near-term solutions.

The RED and osmotic systems, while not onboard-compatible, establish a foundation for bio-inspired ionic energy infrastructure relevant to maritime and coastal UAV operations. A UAV swarm based at a coastal or estuarine site could, in principle, be continuously recharged by a RED stack exploiting the natural river-to-sea salinity gradient, providing an energy infrastructure with no moving parts and no fuel logistics overhead. Realising this scenario requires stack engineering, balance-of-system design, and UAV-side power management advances that currently remain conceptual.

Accordingly, current bio-inspired ionic energy systems should be interpreted primarily as multifunctional auxiliary energy concepts and long-term structural integration hypotheses rather than near-term propulsion-scale replacements. Their value to UAV engineering lies not in immediate deployment, but in establishing biologically validated design principles (ionic selectivity, mechanical compliance, gradient-driven transduction, and conformal form-factor) that may be incorporated into future hybrid energy architectures as the field matures. The engineering feasibility, cross-domain synergy potential, and principal barriers of all approaches reviewed here are systematically evaluated in Section 7.

## 6. Bio-Inspired Energy Harvesting: Photovoltaics and Mechanical Nanogenerators

Complementing the bio-inspired energy storage strategies reviewed in Section 5, this section examines how energy is generated and harvested aboard the UAV platform. Two mechanistically distinct pathways are reviewed: (i) light energy harvesting via bio-structured photovoltaics and photosynthesis-analogous architectures, and (ii) mechanical energy harvesting via triboelectric nanogenerators (TENGs) and piezoelectric nanogenerators (PENGs) integrated with flexible wing structures. The physical couplings between harvesting modules and aeromechanical components (a principal analytical contribution of this paper) are characterised in Section 7.

### 6.1. Light Energy Harvesting: Photosynthesis-Inspired and Bio-Structured Photovoltaics

#### 6.1.1. Photosynthesis-Inspired Architectures and Nanostructured Thin-Film Absorbers

Natural photosynthesis provides one of the most sophisticated biological models for solar energy conversion, with certain light-harvesting complexes (LHCs) exhibiting near-unity energy-transfer efficiency from antenna pigments to reaction centres [98]. Saxena et al. [98] catalogued mechanisms spanning photosynthetic antennas, marine-shell architectures, and deoxyribonucleic acid (DNA)-assembled nanostructures, establishing a framework for translating biological photonic principles into scalable optoelectronic devices and identifying artificial photosynthesis as a promising conceptual pathway towards aerospace energy harvesting.

A compelling structural model for thin-film photovoltaic (PV) enhancement is the wing-scale nanostructure of the black butterfly Pachliopta aristolochiae. Unlike conventional bio-inspired photonic studies that replicate periodic structures, Siddique et al. [99] demonstrated that the disordered, phase-separated arrangement of nanoholes in the wing scales confers superior broadband and wide-angle light absorption relative to periodic counterparts. Through combined microspectroscopy and three-dimensional finite-element optical simulations, the authors quantified a relative integrated absorption increase of approximately +93% at normal incidence (450–800 nm) and +207% at a 50° incident angle in hydrogenated amorphous silicon (a-Si:H) thin-film absorbers [99]. The fabrication employs a scalable self-assembly technique based on lateral phase separation of binary polymer mixtures: a manufacturing route compatible with flexible substrate deposition relevant to UAV wing surfaces.

The extension of bio-structural disorder principles to next-generation PV materials was advanced by Duan et al. [100] who reviewed bio-inspired multiscale design strategies applied to perovskite solar cells (PSCs), drawing structural analogies from butterfly wings, moth-eye antireflection surfaces, and leaf hierarchical textures.

#### 6.1.2. Flexible Quasi-Two-Dimensional Perovskite Solar Cells for UAV Integration

The most significant recent advance in UAV-integrated photovoltaics is the quasi-two-dimensional (quasi-2D) perovskite solar cell developed by Hailegnaw et al. [101], which achieves a champion specific power of 44 W/g and an average of 41 W/g: approximately one to two orders of magnitude above conventional crystalline silicon modules (typical specific power: 0.3–1 W/g). Devices are fabricated directly on an ultrathin polymer foil (<2.5 μm) coated with an alumina barrier layer, yielding a champion power conversion efficiency (PCE) of 20.1% and an open-circuit voltage of 1.15 V. A photovoltaic module consisting of 24 interconnected 1 cm^2^ cells was integrated onto a 13 g quadcopter, demonstrating fully energy-autonomous flight [101]. Unencapsulated small-area devices retained ∼90% of initial PCE after 50 h of continuous MPPT operation in ambient air (ISOS-L-1 protocol), while large-area modules retained ∼74% over the same period [101]. This specific power is one of the highest reported among photovoltaic devices demonstrated on lightweight aerial platforms, and the hardware-validated UAV demonstration establishes quasi-2D PSC technology as the current benchmark for drone-integrated photovoltaics [101].

#### 6.1.3. Flexible Organic Solar Cells and System-Level Demonstrations

At the device level, Tian C. et al. [102] reported a high-efficiency flexible organic solar cell (OSC) module employing an ultra-low sheet resistance transparent conductive electrode (LITO: low-resistance indium tin oxide, <1 Ω/□; 90% transmittance), achieving a 1 cm^2^ single-cell PCE of 17.12% (certified 16.88%) and a 42 cm^2^ module PCE of 15.60%. Stability characterisation demonstrated 97% PCE retention after 1000 bending cycles and 90% retention after 1080 h under the ISOS-D-1 ageing protocol [102]. Integration onto a solar-extended UAV (SUAV) prototype yielded a flight-time extension of 24.2% in outdoor testing [102].

At the system level, Geronel and Bueno [7] compiled evidence from multiple primary studies suggesting that distributed integration of ultra-thin OSCs (specific power > 6 W/g) across wing, fuselage, and tail surfaces can extend UAV endurance by approximately 20%; this figure aggregates results across individual platform demonstrations rather than constituting a single directly-measured system-level value [7]. The most rigorous system-level demonstration of solar-only flight is provided by Liller et al. [103] who developed a battery-free, solar-powered fixed-wing UAV employing capacitive storage and two energy-aware control algorithms (Greedy Energy-Aware Control, GEAC, and Predictive Energy-Aware Control, PEAC) to prevent power brownouts during peak-thrust manoeuvres, enabling a complete flight cycle (take-off, cruise, and landing) powered exclusively by photovoltaic harvesting [103]. However, flexible PSCs face significant mechanical durability barriers: Tian R. et al. [104] identified cracking of rigid indium tin oxide (ITO) transparent electrodes along grain boundaries under repeated bending as a dominant failure mechanism for flexible perovskite devices, while uniform deposition onto aerodynamically optimised wing cambers remains an unresolved manufacturing challenge [104].

### 6.2. Mechanical Energy Harvesting: Bio-Inspired Triboelectric and Piezoelectric Nanogenerators

Mechanical energy harvesting from wing deformation, flutter oscillations, propeller-induced vibration, and ambient airflow provides a complementary power pathway operating independently of solar irradiance. Liu et al. [105] reviewed progress across hybrid harvesting systems combining piezoelectric (strain-to-charge), triboelectric (contact electrification), and electromagnetic (flux-induction) transduction mechanisms, reporting that combining two transduction mechanisms can improve space-utilisation efficiency and output power in representative configurations, while cautioning that the achievable gain in power density and conversion efficiency is strongly configuration-dependent and not yet generalisable [105]. For a resistive load resistance $R_{L}$, with $V_{L}$ denoting the voltage across the load, the instantaneous electrical power delivered by a TENG can be expressed as:

(1) $$P=\frac{V_{L}^{2}}{R_{L}}$$

Because TENGs inherently exhibit high open-circuit voltage ($V_{\mathrm{OC}}$) but high internal impedance ($R_{\mathrm{int}}$), maximum power transfer to an external load is achieved at the impedance-matched condition $R_{L}=R_{\mathrm{int}}$, yielding

(2) $$P_{\max}=\frac{V_{\mathrm{OC}}^{2}}{4R_{\mathrm{int}}}$$

This impedance mismatch between TENG output characteristics and the low-impedance input requirements of conventional electronic subsystems constitutes the principal motivation for the power conditioning architectures reviewed in Section 6.2.8.

#### 6.2.1. Hummingbird-Wing Triboelectric Nanogenerator (H-TENG)

Ahmed et al. [106] demonstrated that the flutter mechanics of hummingbird wings (high-frequency, low-amplitude oscillations confined between paired contact surfaces) constitute a viable model for small-scale wind energy harvesting. The resulting H-TENG device weighs only 10 g and achieves a peak output of 1.5 W/m^2^ at 7.5 m/s in a six-unit network configuration, which the authors demonstrated powering Internet of Things (IoT)-class sensing devices [106]. The mass and output characteristics position H-TENG as a candidate for self-powered sensor nodes on UAV airframes without aerodynamic penalty.

#### 6.2.2. Hierarchical Honeycomb TENG (h-TENG) for Morphing-Wing Integration

**Figure 9:**
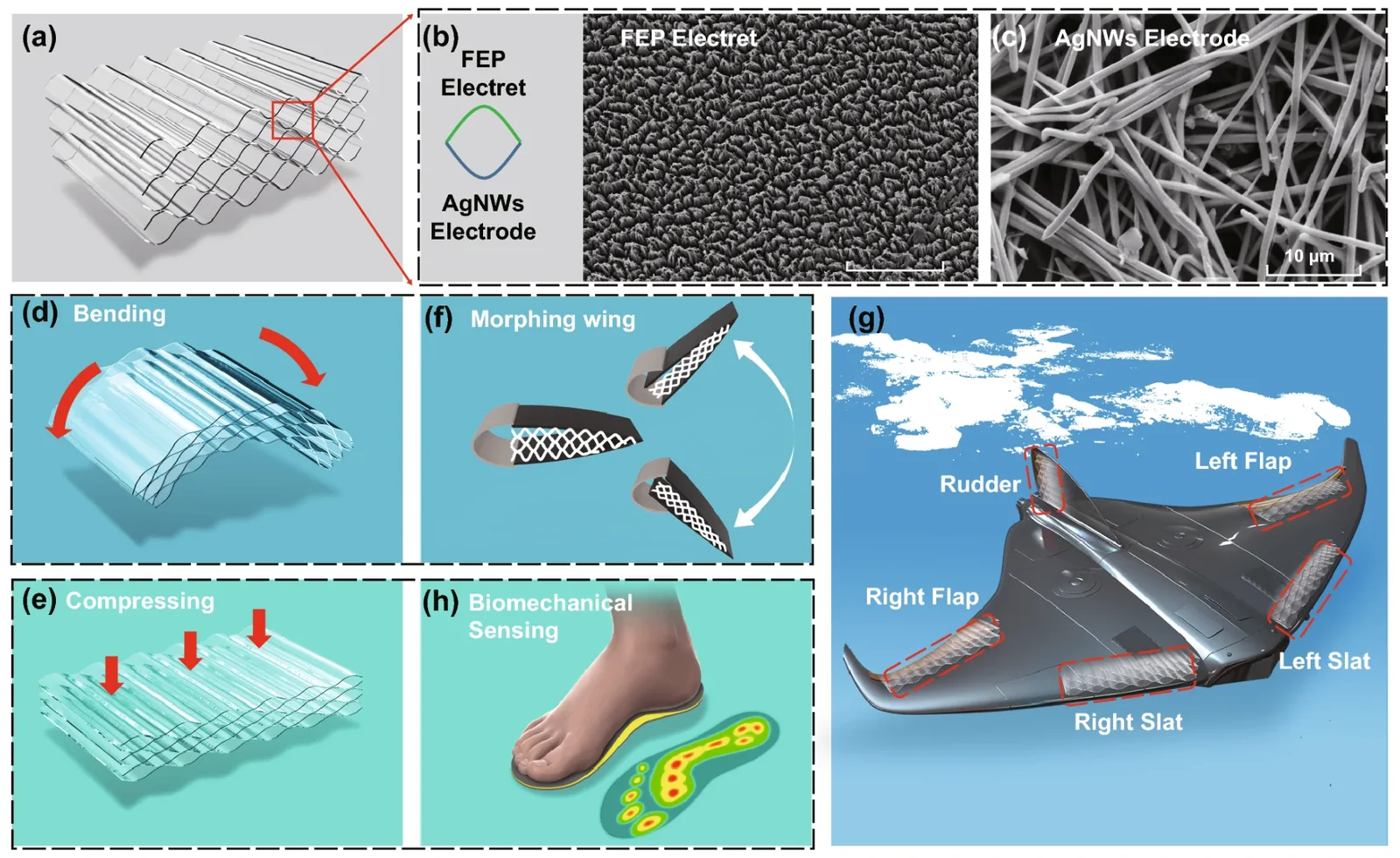
Conceptual overview of a hierarchical honeycomb-structured triboelectric nanogenerator (h-TENG) for biomechanical and morphing-wing energy harvesting. (**a**) Structural design of the honeycomb electret architecture. (**b**,**c**) Scanning electron micrographs of the FEP electret surface and AgNW electrode network. (**d**,**e**) Energy-harvesting mechanisms under bending and compressive deformation. (**f**) Morphing-wing integration concept. (**g**) Implementation of h-TENG skins on UAV control surfaces, including flaps, slats, and rudders. (**h**) Demonstration of biomechanical sensing functionality. It shows the dual role of morphing wing surfaces as both aerodynamic structures and distributed triboelectric energy-harvesting substrates, providing a physical basis for integrated morphing–harvesting architectures in bio-inspired UAV systems. Reproduced from Tao et al. [12].

Tao et al. [12] introduced a hierarchical honeycomb-structured electret/TENG capable of harvesting energy simultaneously from bending and compressive deformation modes (Figure 9). The honeycomb cellular architecture provides mechanical compliance comparable to bio-inspired morphing-wing skin materials, enabling the TENG layer to co-deform with the wing substrate without structural decoupling [12]. This architecture constitutes the physical basis for Coupling Mechanism C-2 identified in Section 7 (morphing wing surface serving simultaneously as a triboelectric energy substrate) and represents the first direct demonstration of dual-functional wing-skin design in the bio-inspired harvesting literature.

#### 6.2.3. Seagull-Wingbeat-Inspired Bistable PENGs

Wu et al. [107] developed a bio-inspired bistable PENG modelled on the distinct upstroke and downstroke phases of a seagull in flight, which impose different force profiles on the wing structure. The bistable configuration (a 301 stainless steel and polyvinylidene fluoride (PVDF) composite beam with imposed displacement limits) enables snap-through transitions that amplify energy conversion from low-frequency (1–20 Hz), low-amplitude, and stochastic vibration inputs, yielding a maximum open-circuit voltage of 11.5 V and a peak output power of 0.734 mW at 18 Hz [107]. Xiong et al. [108] extended this concept with a magnet-free bionic M-shape beam, inspired by the same seagull wingbeat biology, that further reduces stress concentration and improves fatigue resistance. Under optimal conditions, the M-shape harvester produces a maximum open-circuit voltage of 11.5 V and a peak output power of 73 μW: critical reliability metrics for embedded wing structures subjected to millions of flight cycles [108].

#### 6.2.4. Aeroelastic and Wind-Driven Energy Harvesting

Aeroelastic phenomena (flutter, galloping, and vortex-induced vibration) represent physically natural sources of mechanical energy aboard UAV lifting surfaces, exploiting the same fluid–structure interaction mechanisms responsible for wing flexure during flight. Abdelkefi [109] provided a comprehensive review of aeroelastic energy harvesting, cataloguing flutter-based, galloping-based, and vortex-shedding-based harvesters and summarising that representative aeroelastic harvesting devices, as documented across the reviewed literature, typically achieve milliwatt-range continuous output at wind speeds of 4–12 m/s depending on characteristic device dimensions and stiffness parameters [109]. Shan et al. [110] demonstrated a novel aerofoil-based piezoelectric energy harvester with two small square prisms attached to a profiled aerofoil section, achieving two-degree-of-freedom plunge–pitch response and confirming nonlinear limit-cycle oscillation (LCO) as the dominant operating mode. Wind tunnel and field testing confirmed stable LCO-based harvesting over a broad wind speed range, with measured peak outputs in the milliwatt range under laboratory conditions [110]. Liu Q. et al. [111] reported flexible PVDF wing membranes for a flapping-wing micro air vehicle (FWMAV), dividing the wing surface into 16 measurement zones; at a flapping frequency of 12 Hz, the output voltage reflects flutter state without additional sensors, establishing a dual-function (energy harvesting and structural condition monitoring) role for piezoelectric wing skins [111].

#### 6.2.5. TENG Composite Integration in Flapping-Wing Micro Air Vehicles

Zheng et al. [13] presented a nanogenerator-integrated self-powered multi-functional wing system for FWMAVs. Using ultra-thin sandwich structures (total thickness < 20 μm; mass increment < 0.2 g, corresponding to a ∼7% wing-mass increase) with silk-fibroin as the triboelectric friction layer, TENG-integrated wings achieved a peak voltage of 468.7 V, a current of 100.6 μA, and a power output of 379.8 μW. Wind tunnel verification confirmed that TENG integration preserved the aerodynamic properties of the wings while enhancing onboard load capacity by +29.89% [13]. The self-powered sensing capability (enabling flapping frequency measurement via the triboelectric signal) demonstrates that harvested energy can add mission value beyond propulsion power contribution.

#### 6.2.6. Silk-Protein TENG Directly Integrated into a Flapping-Wing Aircraft

The most rigorous validation of TENG-based energy self-consistency in an airborne platform is provided by Zhao and Liu [112]. The authors proposed an Energy Self-Consistent Model (ESCM) for flapping-wing aerial vehicles (FAVs), integrating a silk-protein-based TENG into flexible-wing technology. Over a 100-hour simulated flight evaluation, the TENG-embedded wing (FAV-ESCM) maintained a mean energy output of 40.5 ± 2.2 mW, representing a +36.82% improvement over conventional flexible wings without TENG integration (29.6 ± 3.4 mW), with a reported energy-recovery accuracy of up to 93.5% [112]. The selection of silk protein as the triboelectric friction layer (exhibiting high surface charge density, biodegradability, and mechanical flexibility relative to synthetic polymer electrets) itself constitutes a material-level biomimetic decision independent of the structural design.

#### 6.2.7. PVDF Piezoelectric Transducers on UAV Propulsion Systems

A distinct approach exploits propeller-induced airflow and motor vibration rather than wing deformation. Goncalves et al. [113] integrated PVDF piezoelectric transducers coupled to a commercial energy harvesting power management unit (LTC3588-1) onto a multirotor UAV arm. At a motor rotational speed of 3975 rpm, the transducers generated a voltage amplitude of 17.3 V, charging a coupled capacitor to the maximum regulated output voltage in approximately 162 s [113]. This study established that propeller-/motor-induced mechanical energy can sustain ultra-low-power onboard sensors while reserving the battery entirely for propulsion, thereby establishing a partitioned energy architecture that extends operational autonomy at the subsystem level without modifying the main powertrain.

#### 6.2.8. Power Management and Conditioning Circuits

The high-voltage, low-current, pulsed output of TENGs and PENGs presents significant power conditioning challenges. Dien et al. [114] reviewed recent progress in TENG energy storage interfaces, reporting that synchronous electric charge extraction (SECE) circuits increase effective power output by up to fivefold relative to conventional full-bridge rectifiers, and that transformer-based impedance matching yields a fivefold power transfer improvement with a 40% reduction in Joule heating losses [114]. Satpute et al. [115] demonstrated that bio-inspired microparticle coatings on triboelectric electrodes achieve 0.262 mW/m^2^ at 0.5–6 Hz in a sliding-mode TENG configuration, introducing a bio-material design paradigm with potential advantages in surface charge density and material availability [115]. Looking ahead, AI-driven maximum power point tracking (MPPT) and neuromorphic impedance optimisation (a coupling with the neuromorphic control systems reviewed in Section 4) represent high-leverage research directions for maximising harvester yield without hardware modification.

### 6.3. Self-Powered UAV Systems and Energy-Neutral Flight Strategies

Wei et al. [116] provided a systematic review of energy harvesting as the enabling technology for self-powered UAV platforms, categorising solar cell placement topologies, flapping-wing motion recovery, airstream-induced vibration, and bio-inspired DS as the four principal harvesting modalities [116]. Hassanalian and Abdelkefi [117] framed endurance as the central design constraint across all UAV size classes, noting that energy consumption scales non-linearly with payload and structural mass: a relationship that makes supplementary harvesting proportionally most impactful in lightweight (<5 kg) platforms [117].

The battery-free solar demonstration by Liller et al. [103] represents one of the most integrated photovoltaic UAV demonstrations reported to date, eliminating electrochemical storage entirely. Its principal limitation is complete loss of operational capability at night and severely degraded performance under partial cloud cover. The flexible OSC module of Tian C. et al. [102] was directly demonstrated on a SUAV platform, constituting hardware-validated integration of next-generation flexible PV onto a drone; the principal challenge is the coupling between PV laminate bending stiffness and wing aeroelastic behaviour [102].

The ESCM framework of Zhao and Liu [112] enables a rigorous assessment of TENG energy contribution relative to total FAV consumption. The reported mean output of 40.5 ± 2.2 mW over 100 simulated flight hours must be contextualised against typical small UAV avionics power consumption (flight computers, inertial measurement units (IMUs), and communication modules collectively: 5–25 W) and propulsion requirements (50–500 W depending on vehicle class and mission) [116]. TENG harvesting, therefore, constitutes a supplementary source capable of sustaining low-power onboard subsystems (sensors, telemetry, lightweight actuators) rather than contributing to propulsion energy. Perez et al. [118] confirmed that quadcopter piezoelectric arm-integrated harvesters achieve up to 5.35 mW during stationary flight, identifying applications in battery trickle charging, structural health monitoring, and vibration damping, establishing piezoelectric harvesting as a multi-function rather than single-purpose technology [118].

A prospective integration scenario couples the aerodynamic energy recovery strategies reviewed in Section 3 (particularly albatross-inspired DS) with TENG-based airflow harvesting during low-power glide phases. During DS, reduced propulsion demand may increase the relative relevance of boundary-layer airflow harvesting, although its quantitative contribution remains to be experimentally verified [116]. Neuromorphic control systems (Section 4), by substantially reducing avionics power consumption, increase the relative contribution of harvested energy to the net energy balance: an indirect coupling related to Coupling Mechanism C-4 that merits systematic experimental characterisation in future programmes.

### 6.4. Comparative Analysis: Capabilities, Limitations, and Bridge to Section 7

What the evidence establishes. Several performance claims in bio-inspired energy harvesting have now been validated at a level sufficient to inform engineering design. The disordered nanohole approach of Siddique et al. [99] provides a laboratory-validated optical enhancement principle (approximately +93% to +207% absorption improvement) with a reproducible fabrication route. Hailegnaw et al. [101] demonstrated energy-autonomous drone flight using quasi-2D PSC technology, achieving a specific power of 44 W/g: a value that exceeds the specific power of conventional crystalline silicon modules by approximately one to two orders of magnitude and represents one of the highest reported among photovoltaic devices demonstrated on lightweight aerial platforms [101]. The flexible OSC module of Tian C. et al. [102] achieved a PCE of 17.12% with 97% retention after 1000 bending cycles, directly demonstrated on a SUAV with 24.2% endurance extension [102]. The H-TENG of Ahmed et al. [106] provides a mass-budget-compatible (10 g) device at 1.5 W/m^2^. Most significantly, Zhao and Liu [112] validated stable TENG energy delivery (40.5 ± 2.2 mW, up to 93.5% energy-recovery accuracy) over 100 simulated flight hours, providing long-duration evidence of energy-output stability for wing-integrated mechanical harvesting. Liller et al. [103] demonstrated solar-only sustained flight at system level.

What remains unproven. The gap between demonstrated TENG/PENG output densities (tens to hundreds of microwatts to milliwatts) and practical UAV propulsion requirements (tens to hundreds of watts) spans three to four orders of magnitude: no published study has demonstrated that triboelectric or piezoelectric harvesting alone contributes measurably to propulsion energy in any UAV class [105,116]. The disordered nanohole PV architecture has not been characterised under representative outdoor UAV conditions: sustained ultraviolet (UV) flux, combined mechanical vibration and thermal cycling, and precipitation exposure. The ITO electrode cracking failure mode identified by Tian R. et al. [104] and the insufficient maturity of miniaturised TENG power conditioning circuits [114] represent the principal near-term engineering barriers.

Emerging pattern and bridge to Section 7. A notable pattern emerging from Table 8 and Table 9 is that structurally and materially grounded forms of bio-inspiration currently appear closer to near-term UAV integration than abstract functional analogies. Technologies that closely replicate the structural or material properties of their biological counterparts (silk-protein TENG, disordered nanohole PV, seagull-inspired bistable PENG) currently occupy more advanced integration levels than those relying on functional analogy alone (artificial photosynthesis, flexible PV benchmarks). This suggests that material-level and structural-level bio-inspiration is currently driving practical progress in UAV energy harvesting more effectively than high-level functional analogies. Integration of morphing wing structures as triboelectric substrates [12,112] and the dual-functional role of flexible photovoltaic surfaces [101,102] bridge the aeromechanical axis (Section 2, Section 3 and Section 4) and the energy axis (Section 5 and Section 6); whether these coupling mechanisms yield measurable endurance gains at the system level is the central question of Section 7.

## 7. Cross-Domain Synergy Analysis: Towards System-Level Bio-Inspired UAV Integration

The preceding sections (Section 2, Section 3, Section 4, Section 5 and Section 6) reviewed bio-inspired technologies across the principal technical domains: aeroacoustic noise reduction, bio-inspired flight strategies, adaptive structures, neuromorphic sensing and control, and bio-inspired energy storage and harvesting. A recurring observation across these domains is that the relevant technologies are not independent: the same coherent vortex structures responsible for propeller noise can also contribute to propulsive aerodynamic losses; the same structural deformations enabling morphing wings produce recoverable mechanical energy that triboelectric films can transduce and the same sparse computation architecture that reduces neuromorphic inference energy also reduces the electrical demand that onboard energy sources must supply. Such an integrative perspective is absent from prior biomimetic UAV reviews [7,116], which have addressed individual technologies without characterising the emergent properties arising at their intersections. This section provides an integrative synthesis identifying and characterising six mechanisms (five performance couplings (C-1 through C-5) and one structural enabling architecture (E-1)) that emerge at these intersections, assessed using quantitative evidence from primary literature.

The six mechanisms are organised according to their dominant linking role. Physical Couplings (C-1, C-2, C-5) arise where two performance attributes share a common physical origin or co-located substrate. C-3 is retained within the five-coupling framework as a long-horizon system-level complementarity pathway: bio-inspired flight strategies may reduce overall primary-energy demand, but they do not change the independent output capability of the ionic-gradient source. C-4 is a System Compatibility Coupling in which low-energy neuromorphic sensing and computation reduce avionics power demand within the shared energy-constrained UAV architecture. The Structural Enabling Architecture (E-1) provides the multifunctional material platform required for C-2 and C-5 to coexist on the same aerodynamic surface. This classification is not intended as a rigid taxonomy; it is an analytical framework whose purpose is to organise technology interactions according to their dominant linking role, including shared physical processes, system-level complementarity or demand-side compatibility, and structural co-location.

### 7.1. C-1: Aeroacoustic–Propulsive Physical Coupling

Aerodynamic noise and propulsive drag arise from related but not identical flow mechanisms (principally blade–wake interaction (BWI) and the turbulent vortex structures generated at blade surfaces), constituting the aeroacoustic–propulsive physical coupling. Candeloro et al. [4] established the mechanistic foundation, classifying BWI-induced tonal noise at blade-passing frequencies and boundary-layer-transition broadband noise as the two primary targets for passive surface modification in low-Reynolds-number drone propulsors: both susceptible to owl-feather-inspired serration geometries.

Wei et al. [6] demonstrated this coupling most directly: a three-dimensional sinusoidal serration propeller integrating owl feather morphology with cicada forewing geometry simultaneously reduced OASPL by up to 5.5 dB at 5 m and reduced mechanical power consumption by 20.2% relative to the DJI Phantom 3 benchmark at 50 gf thrust (22.6% at 150 gf). Wei et al. [5] demonstrated that simultaneously applying LE and TE serrations in a BCP achieves a maximum noise reduction of 3.1 dB in laboratory conditions, a 7.9% improvement in the average force-to-speed ratio ($I_{g}$), and 2.6–3.8 dB suppression in outdoor quad-rotor hovering tests at 3–12 m. Spectral analysis confirmed dual suppression of tonal noise at the fundamental blade-passing frequency and broadband noise above 2 kHz. Wei et al. [19] established generality across five serration geometries: sawtooth designs achieved an average 2.43 dB noise reduction (maximum 4.18 dB) with a concurrent 3.53% thrust increase, confirmed in UAV hover tests at 4.73 dB and 3.79 dB reductions at 5 m and 8 m altitude.

Gu et al. [25] extended the analysis to trailing-edge serration configurations on a DJI 9450S propeller, systematically testing multiple variants across serration shape, ratio, and spanwise coverage, and identifying the optimal round-serrated design (serration ratio 0.9, 50% spanwise coverage) as achieving up to 4.4 dBA noise reduction. LES-based CFD analysis confirmed that the serrated trailing edges disrupt propeller tip vortices, expediting vortex shedding and dissipation and promoting the intermixing of flow structures, thereby reducing broadband trailing-edge noise. Wang et al. [119], working in a mixed-flow fan context, mechanistically distinguished LE serrations (which suppress Kelvin–Helmholtz instability and reduce broadband noise across 40 Hz–4 kHz) from TE serrations operating via distinct vortex-shedding dissipation mechanisms, providing the physical basis for the superior combined performance of Wei et al. [5]. At the wing scale, Miklosovic et al. [26] established through wind tunnel measurements on a humpback whale (Megaptera novaeangliae) flipper model that leading-edge tubercles delay stall by approximately 40% while increasing lift and reducing drag, extending the dual-benefit principle from propellers to fixed-wing UAV surfaces.

A 20.2% reduction in shaft power, if preserved after motor–electronic speed controller (ESC) integration losses and across the propulsion operating envelope, would reduce electrical draw at the propulsion stage. For a battery-electric UAV, a first-order endurance estimate can be expressed as

(3) $$t_{\mathrm{end}}\approx \frac{E_{\mathrm{usable}}}{{\bar{P}}_{\mathrm{total}}}$$

where $t_{\mathrm{end}}$ is flight endurance; $E_{\mathrm{usable}}$ is the usable onboard electrical energy available for the mission; and ${\bar{P}}_{\mathrm{total}}$ is the mission-averaged total electrical power demand, including propulsion, avionics, sensing, computation, payload, and conversion losses. Accordingly, a propeller-level shaft-power reduction does not translate one-to-one into endurance gain. The resulting endurance improvement depends on the fraction of total electrical demand attributable to propulsion, motor–ESC efficiency, thrust-equivalent operating conditions, battery discharge behaviour, added mass, auxiliary loads, and the mission profile. Equation (3) is, therefore, used only as a first-order battery-electric energy-budget relation, not as a complete vehicle-specific endurance model. It should further be noted that noise reduction and aerodynamic efficiency are not universally correlated across serration geometries: certain configurations in the broader aeroacoustic literature exhibit performance trade-offs rather than simultaneous gains, underscoring the necessity of design-specific empirical validation. Critically, all C-1 experimental validations apply to hover or near-zero-advance-ratio conditions; forward-flight performance under inflow turbulence and varying BWI geometry remains entirely unvalidated.

### 7.2. C-2: Morphing Wing Deformation–Triboelectric/Piezoelectric Physical Coupling

Morphing wing structures undergo periodic mechanical deformation under aerodynamic loading: a recoverable mechanical energy source that triboelectric nanogenerators (TENGs) and piezoelectric nanogenerators (PENGs) can transduce. Unlike C-1, where both performance benefits arise from a shared flow-structural mechanism, C-2 is a co-located transduction coupling: the same deformation required for aerodynamic shape adaptation also drives energy harvesting, with the aerodynamic surface serving simultaneously as the conversion substrate, thereby reducing the need for dedicated off-wing generators, although the TENG films, wiring, encapsulation, and power-management circuits still impose non-negligible integration costs. The biological precedent is the elastic energy storage in avian feather shafts and bat wing membranes, in which passive structural compliance and energy management coexist within the same morphological element.

**Figure 10:**
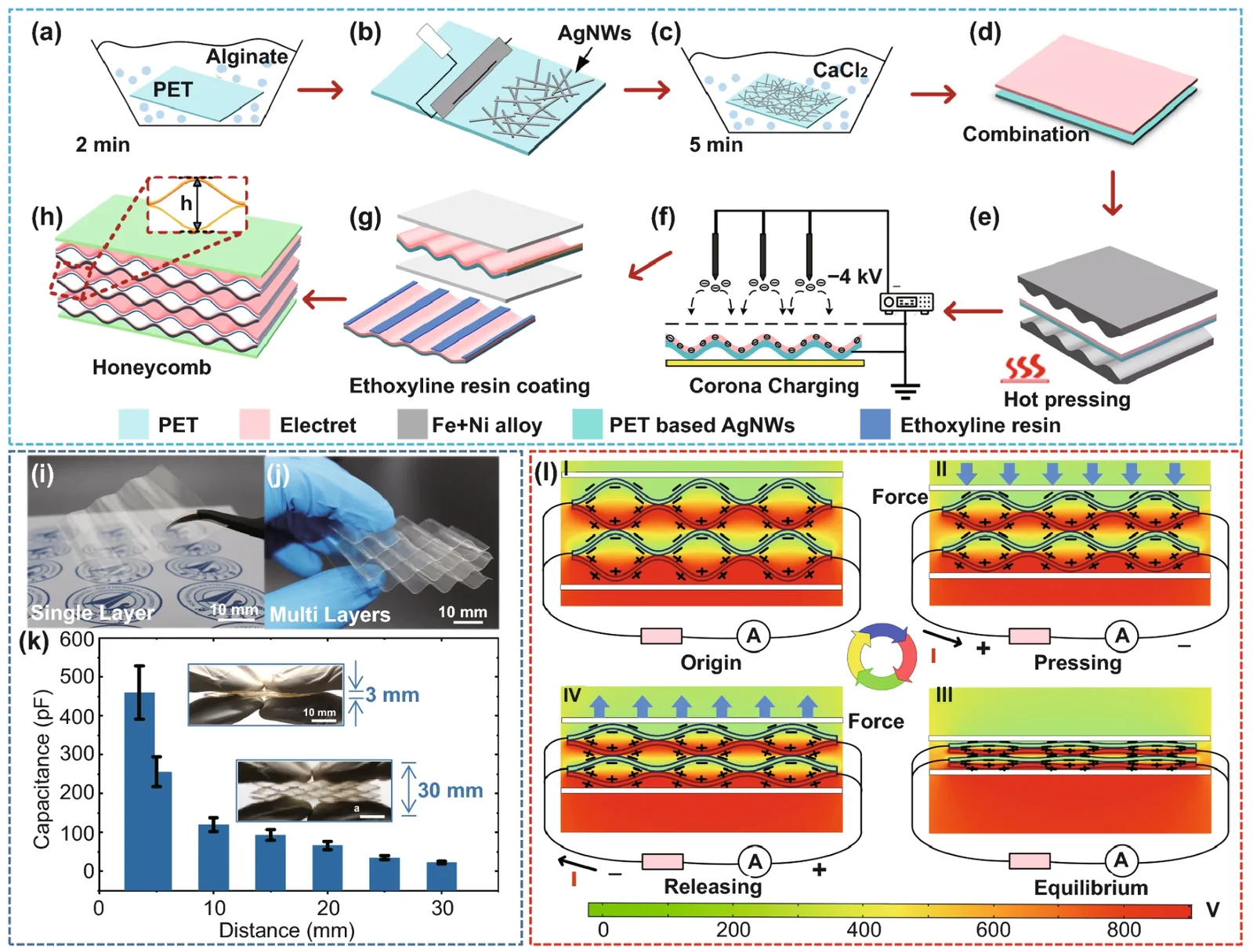
Fabrication process and operating mechanism of the hierarchical honeycomb-structured triboelectric nanogenerator (h-TENG) for morphing-wing energy harvesting. (**a**–**h**) Sequential fabrication procedure, including PET substrate preparation, AgNW deposition, CaCl_2_-assisted bonding, hot pressing, corona charging, resin coating, and honeycomb assembly. (**i**,**j**) Photographs of single-layer and multilayer h-TENG architectures. (**k**) Capacitance variation as a function of structural separation distance. (**l**) Charge redistribution during cyclic compression and release, illustrating the triboelectric energy-conversion mechanism. It illustrates the physical basis for deformation-induced energy harvesting on morphing UAV surfaces and shows how structural deformation can be directly converted into electrical output. Reproduced from Tao et al. [12].

Tao et al. [12] achieved an early TENG–UAV morphing-wing integration using a hierarchical honeycomb-structured h-TENG, which produced 275 μW at a 30° deflection angle in the morphing-wing test. This result should be distinguished from the separate hand-tapping benchmark, where the device reached 1207 V and 12.4 mW, corresponding to 0.275 mW cm^−3^ and 2.48 mW g^−1^ (Figure 10). Zheng et al. [13] demonstrated TENG integration in FWMAV wings as an ultra-thin sandwich structure (<20 μm added thickness, +7% wing mass), yielding 379.8 μW at a peak open-circuit voltage of 468.7 V (100.6 μA) while enhancing the FWMAV onboard load capacity by 29.89%. Liu et al. [111] complementarily established PVDF-based piezoelectric harvesting and self-powered sensing for flapping-wing MAVs, generating up to 21 μW at 6 V RMS at 12 Hz flapping frequency, with the harvester output simultaneously reflecting the wing flutter condition, confirming that structural–energy integration can actively support self-powered onboard sensing functions. Perez et al. [118] extended the concept to rotary-wing platforms: piezoelectric transducers substituted into DJI F450 quadcopter arm components harvested up to 5.35 mW during hover, demonstrating practical energy recovery without propulsion modification. Ahmed et al. [106] established the bio-inspired precedent at UAV-relevant scales through a hummingbird flutter-driven TENG (10 g, 1.5 W $m^{-2}$ at 7.5 m/s). Wu et al. [107] proposed a seagull wingbeat-inspired bistable PENG targeting the 1–20 Hz UAV structural vibration frequency range, achieving 0.734 mW at 18 Hz; the bistable architecture substantially reduces stress concentration relative to conventional cantilever designs, directly advancing the long-term durability case for operational durability (Barrier B2, Section 7.7). Singh et al. [120] provided complementary analytical treatment through modal analysis of a NACA 2412 fixed-wing UAV, identifying optimal spanwise piezoelectric patch locations for maximum voltage output. Under UAV-relevant or structurally integrated operating conditions, the harvested power levels generally span hundreds of microwatts to a few milliwatts, whereas higher benchmark outputs reported under hand-tapping or other laboratory excitation modes should not be interpreted as direct propulsion-relevant power.

The harvested power levels (spanning hundreds of microwatts to a few milliwatts) are insufficient for propulsion contributions on small platforms where propulsion operates at 10–100 W. C-2 is, therefore, near-term viable primarily for self-powered distributed sensing, structural health monitoring, and low-power auxiliary electronics. Net propulsion-level endurance extension requires demonstration that energy output exceeds both the mass penalty and aeroelastic modification costs: a balance not yet achieved across any cited study. Three barriers persist: (i) the net mass–energy balance remains unquantified at full system level (Zheng et al. [13] constitute a partial exception); (ii) TENG-embedded surfaces may alter aeroelastic response; and (iii) performance retention over 10^4^–10^6^ operational bending cycles is undocumented.

### 7.3. C-3: Bio-Inspired Flight Strategy–Low-Power Storage Complementarity Pathway

C-3 is retained within the five-coupling framework as a long-horizon system-level complementarity pathway, not a demonstrated source-level compatibility coupling or a quantitative compatibility threshold. Bio-inspired flight strategies, including albatross dynamic soaring and V-formation flight, can reduce the energy demanded from the primary propulsion source by exploiting atmospheric wind gradients and inter-aircraft aerodynamic interactions, respectively. Such reductions, however, do not increase the electrical output capability of an ionic-gradient source and, therefore, cannot by themselves make that source capable of sustaining onboard avionics.

Han et al. report approximately 135 mV open-circuit voltage per solid-state artificial electrocyte and demonstrate voltage scaling through integration of multiple electrocytes, including an open-circuit voltage of approximately 22 V from a 165-unit series array [80]. These results demonstrate electrical scalability but do not establish UAV-relevant sustained regulated load power, gravimetric energy density, or mission-level energy delivery. For an ionic-gradient source to sustain an avionics load independently, its usable conditioned output must satisfy

(4) $$\eta_{\mathrm{pc}}P_{\mathrm{ion},\mathrm{src}}\geq P_{\mathrm{av}}$$

together with sufficient usable energy over the required mission duration. Here, $P_{\mathrm{ion},\mathrm{src}}$ is the sustained loaded electrical power available from the ionic-gradient source before power conditioning, $\eta_{\mathrm{pc}}$ is the efficiency of the required power-conditioning stage, and $P_{\mathrm{av}}$ is the avionics electrical demand. The currently available data do not permit this condition to be evaluated for a representative UAV avionics load.

At the system level, the complementarity can be represented by the following simplified power-budget relation introduced in this review:

(5) $$P_{\mathrm{primary}}=\left(1-s\right)P_{\mathrm{prop}}+\max\;\left[0,P_{\mathrm{av}}-\eta_{\mathrm{pc}}P_{\mathrm{ion},\mathrm{src}}\right]$$

In this simplified two-load power-budget representation, the baseline propulsion-power demand is denoted by $P_{\mathrm{prop}}$, and *s* (0≤s<1) is the mission-averaged fractional reduction in propulsion-power demand relative to the baseline flight condition. The formulation separates the two mechanisms: dynamic soaring reduces the propulsion contribution to primary-source demand, whereas the ionic-gradient source can offset part or all of the avionics load only if sufficient loaded power and usable energy are independently available. By construction, propulsion-side savings enter only the first term and, therefore, cannot relax the independent avionics-support condition in Equation (4); any ionic-gradient output in excess of $P_{\mathrm{av}}$ is not credited to propulsion in this simplified relation. Consequently, no universal percentage of propulsion-power saving can presently be specified that would make the reported ionic-gradient architecture, with approximately 135 mV open-circuit voltage per unit and approximately 22 V open-circuit voltage for the 165-unit series array, capable of sustaining onboard avionics. Equation (5) is a conceptual system-level power-budget relation introduced in this review, not a validated design model or a prediction of ionic-gradient battery performance.

**Figure 11:**
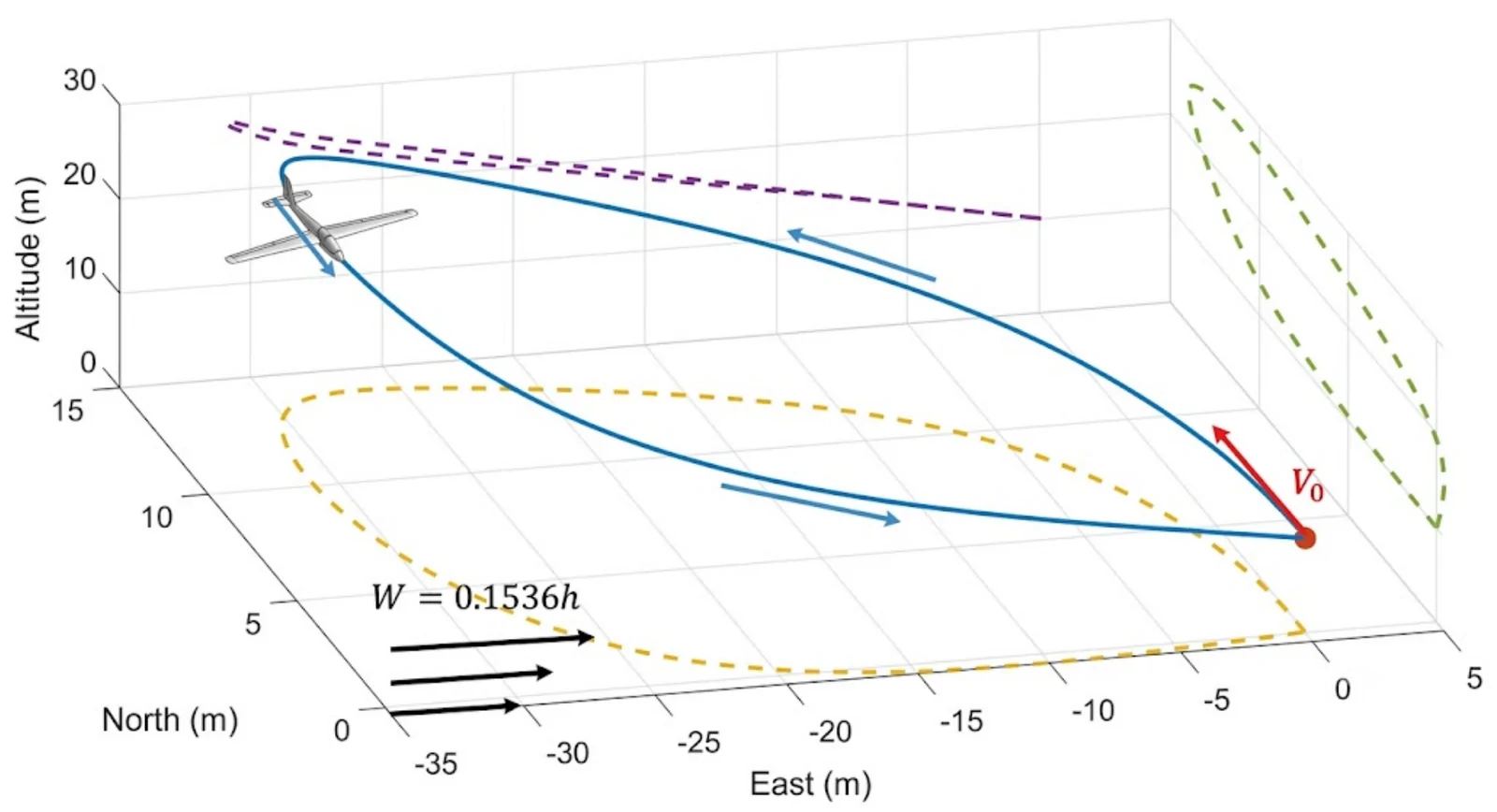
Closed-loop dynamic-soaring trajectory under the minimum wind-gradient condition required for sustained energy-neutral flight. The optimised flight path consists of four recurring phases: windward climb, high-altitude turn, leeward descent, and low-altitude turn. By repeatedly traversing atmospheric wind-shear layers, the UAV extracts environmental energy and reduces net propulsive power demand. The trajectory illustrates the aerodynamic basis of bio-inspired DS and highlights the sensing, estimation, and trajectory-planning requirements necessary for autonomous implementation. Adapted from Wang et al. [123].

Deittert et al. [121] established the theoretical foundation for engineless UAV propulsion by DS, demonstrating via differential flatness of the flight dynamics model that optimal trajectories for maritime wind-gradient environments are computationally tractable and remain within practical controllability limits (notably bank-angle and altitude constraints). Wang et al. [122] characterised morphing-augmented soaring aerodynamics via RANS simulation: anhedral wingtip morphing reduced the drag coefficient by 7.75% and decreased downwash intensity by 30.32%, with flight simulation reporting a 47.99% peak energy gain improvement and 13.05% endurance increase per soaring cycle. These results are entirely simulation-derived and require experimental validation before deployment implications can be quantified. Wang et al. [123] quantified parameter sensitivity, finding that UAV mass positively influences DS energy harvesting while initial airspeed and entry angle govern both efficiency and trajectory shape (Figure 11). Hong et al. [51] advanced practical feasibility through a quadratic programming-based free-cycle soaring trajectory optimiser requiring only instantaneous wind information, removing the requirement for pre-characterised wind gradient profiles and substantially extending the applicability of autonomous DS to unstructured atmospheric environments.

For V-formation flight, Portugal et al. [52] provided direct biological evidence from northern bald ibises (*Geronticus eremita*): individuals position themselves at aerodynamically optimum spanwise locations, synchronise wingbeat phase to maximise upwash capture throughout the entire flap cycle, and adopt anti-phase timing when flying directly behind a conspecific to avoid downwash, confirming that the formation energy benefit is achieved through real-time sensory feedback rather than passive geometric alignment. Qiao et al. [124] quantified engineering gains through variable-fidelity CFD: lift-to-drag ratio improvements of 22.8% in a two-aircraft unit and 57.5% in a three-aircraft unit, with average L/D improvement exceeding 19%.

On the storage side, Xiao et al. [85] demonstrated an electric-eel-type BGB achieving 0.54 V per unit, scaling to 22 V via a 56-unit Miura-ori folded assembly or up to 60 V via a 126-unit planar first-and-tail stacking configuration. The Han et al. [80] architecture and its present power-delivery limitations are characterised in the opening framework above. C-3, therefore, remains a long-term research direction in which advances in flight-strategy algorithms, control, and ionic-gradient source capability may eventually converge. It should not be interpreted as a currently realisable engineering integration or as evidence that a particular dynamic-soaring power reduction would make the present ionic-gradient architecture viable for avionics. C-3 represents system-level complementarity rather than direct enabling compatibility, and its present value is as a system-level co-design boundary condition.

### 7.4. C-4: Neuromorphic Computation–Avionics Power Budget Compatibility Coupling

SNNs, which emulate the sparse asynchronous signalling of biological neural circuits, offer computation energy per inference that is orders of magnitude lower than conventional ANN implementations, constituting a coupling between the neuromorphic computation paradigm and the UAV avionics power budget. Davies et al. [125] established the computational foundation: brain-inspired networks employing recurrence, spike-timing, and sparsity on the Loihi neuromorphic processor achieve orders of magnitude lower energy and latency than conventional approaches, with the advantage specifically associated with bio-inspired network architectures. Event cameras, characterised by Gallego et al. [126] as bio-inspired silicon retina sensors with microsecond-resolution asynchronous events, 140 dB dynamic range, and intrinsically low power consumption, provide the perceptual foundation for this coupling.

Paredes-Vallés et al. [14] translated this advantage to autonomous drone flight: a fully neuromorphic vision-to-control pipeline operated on board at 200 Hz and was reported to spend 27 μJ per network inference. A separate offline energy benchmark on a 32-chip Nahuku evaluation board measured execution frequencies of 274–1637 Inf/s and approximately 7.2–27.2 μJ per inference depending on input event density, corresponding to three to four orders of magnitude lower inference energy than an NVIDIA Jetson Nano platform running the same SNN workload in software. Sanyal et al. [127] extended the paradigm through EV-Planner, combining dynamic vision sensor (DVS) event cameras, a shallow SNN, and a physics-guided neural network embedding brushless direct-current (DC) motor equations to generate energy-optimal trajectory plans for gate-navigation and obstacle avoidance. Stroobants et al. [69] deployed a recurrent end-to-end SNN for attitude estimation and control on a Crazyflie quadrotor at 500 Hz, achieving an attitude tracking RMSE of 3.03° at only 15% neuronal spiking activity: a level of sparse activation closely associated with the inference energy advantage achievable on dedicated neuromorphic hardware. Vitale et al. [15] further demonstrated a neuromorphic SNN for UAV visual line-tracking deployed on the Intel Loihi chip (Kapoho Bay platform), achieving up to three orders of magnitude better speed–power tradeoff than conventional image sensors and realising on-chip adaptation via synaptic plasticity, providing additional evidence that the energy efficiency advantage is a property of the spike-based, event-driven computation paradigm itself.

A critical qualification is required: propulsion typically consumes 80–95% of a small UAV’s total power budget, and quantitative system-level endurance gains attributable solely to neuromorphic computing have not been demonstrated in any flight experiment. The primary near-term value of C-4 lies in enabling low-latency perception, reducing onboard thermal load, and providing compatibility with distributed low-power sensing subsystems. More strategically, the high-bandwidth event-based sensing enabled by C-4 may be well-suited to the real-time wind-field estimation and adaptive trajectory planning required for autonomous DS and V-formation coordination, although direct demonstrations of SNN-based control in these flight modes are entirely absent from the current literature. This dependency renders C-4 maturation a prerequisite for C-3 feasibility, establishing the two couplings as sequential rather than parallel research priorities.

### 7.5. C-5a/C-5b: Bio-Inspired Photonic Nanostructure–Photovoltaic Coupling

C-5 is presented as two sub-components of a single coupling, C-5a and C-5b, distinguished by maturity level rather than by mechanism; both concern the same bio-inspired photonic-to-photovoltaic pathway and are together counted as one of the five performance couplings.

Bio-inspired photonic nanostructures are relevant to UAV endurance because solar-powered UAV performance is fundamentally constrained by incident energy capture efficiency, and the angular robustness of disordered bio-inspired designs addresses a specific limitation of conventional periodic grating structures under continuously varying irradiance angles. This coupling is most relevant not to conventional short-endurance multirotors but to long-endurance fixed-wing or high-altitude solar-assisted UAVs, where improvements in angle-tolerant light absorption can accumulate over extended mission durations to potentially contribute to net energy availability: if performance can be maintained after cell integration and airframe-level implementation.

Siddique et al. [99] demonstrated that disordered nanohole arrays derived from the wing scales of *Pachliopta aristolochiae* (a disordered nanohole pattern transferred to a fabricated a-Si:H absorber with mean nanohole diameter 238 ± 105 nm) achieve a relative integrated absorption increase of 93% at normal incidence (450–800 nm), rising to +207% at 50° angle of incidence when transferred to thin-film hydrogenated amorphous silicon absorbers. As the authors explicitly acknowledge, this absorber-layer optical enhancement does not directly translate to an equivalent full-cell photovoltaic efficiency improvement, which is additionally governed by front-surface reflection losses, electrical contact resistance, and minority carrier recombination dynamics. Kumar et al. [128] extended the approach to plasmonic butterfly-wing-shaped nanostructures in organic solar cells, reporting a measurable increase in short-circuit current density (Jsc): a device-level metric more directly relevant to UAV energy harvesting than absorber-layer characterisation alone.

C-5 encompasses two sub-components of differing maturity. The disordered nanohole absorber architecture of Siddique et al. [99] remains a long-term research priority, conditional on advances in scalable flexible-substrate fabrication and performance retention under UAV operational conditions. By contrast, quasi-2D perovskite solar cell integration has been demonstrated directly on a UAV platform [101] and is accordingly assessed as a mid-term priority, with large-area encapsulation and operational stability constituting the principal remaining barriers.

### 7.6. E-1: Structural Enabling Architecture: Avian-Inspired Multifunctional Morphing Composites

E-1 is not an independent performance coupling but the material and structural platform that physically enables C-2 (TENG/PENG harvesting) and C-5 (photovoltaic nanostructure coating) to be implemented simultaneously on the same aerodynamic surface, maximising functional density per unit airframe mass. Without a suitable multifunctional morphing composite substrate, neither C-2 nor C-5 can advance beyond isolated component-level demonstrations to integrated flight systems.

**Figure 12:**
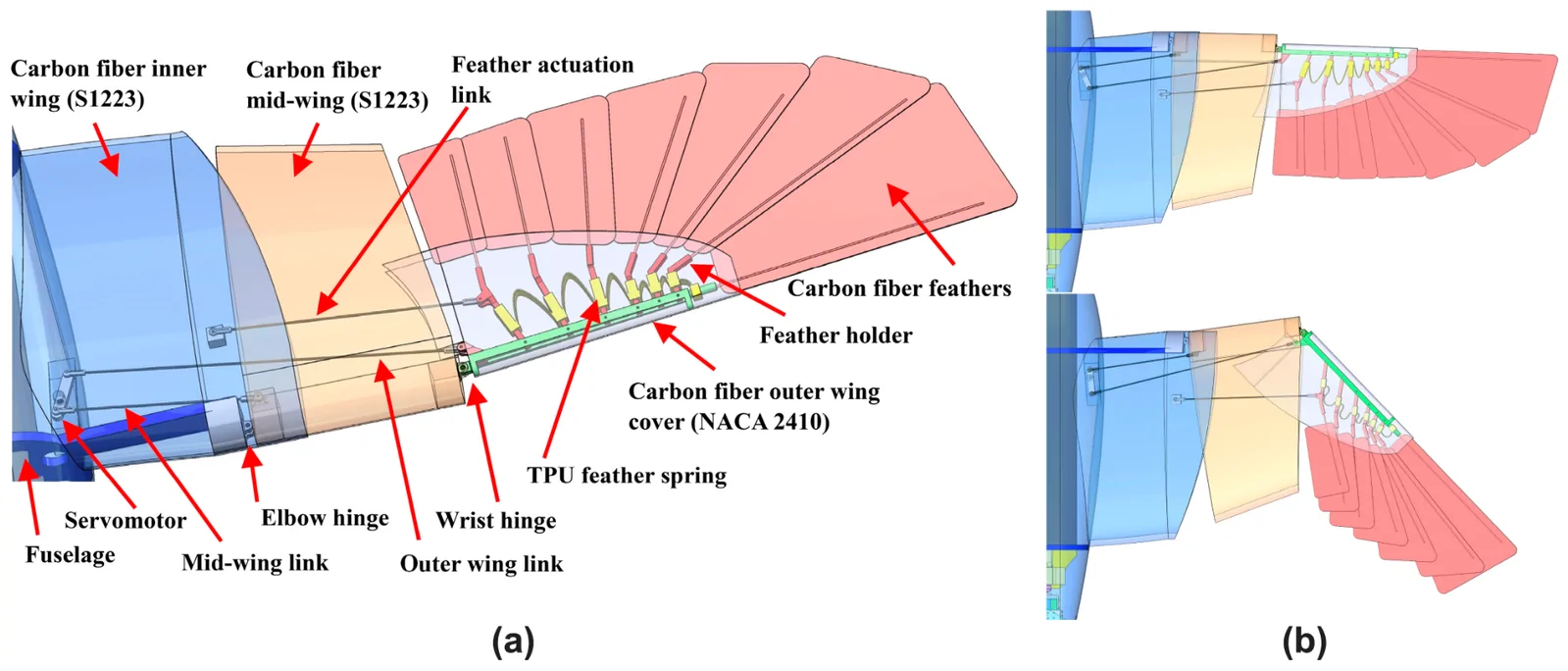
Structural enabling architecture of the CGull bio-inspired morphing UAV. (**a**) Detailed wing assembly showing the carbon-fibre inner wing, mid-wing, feathered outer wing, linkage system, hinges, feather actuation mechanism, and compliant spring elements responsible for coordinated morphing. (**b**) Comparison between extended and tucked wing configurations, demonstrating the large geometric variation achievable through the coupled morphing mechanism. The continuous wing surface provides a multifunctional structural substrate capable of simultaneously supporting aerodynamic load bearing, shape adaptation, and future integration of energy-harvesting or photovoltaic functional layers. Adapted from Bishay et al. [129].

Bishay et al. [129] demonstrated CGull, a flight-tested bio-inspired composite morphing UAV whose carbon-fibre wings replicate the two degree-of-freedom sweep pattern of the Great Black-Backed Gull (*Larus marinus*): a coupled mechanism advances the mid-wing forward and sweeps the outer feathered wing backwards, with successful flight tests validating path control via the bio-inspired morphing system (Figure 12). Bishay et al. [130] further developed 3D-printed corrugated flexible structures with articulated artificial feathers as a lower-cost fabrication route for bird-like morphing UAV prototyping. Although carbon-fibre composites are not inherently biomimetic, their high stiffness-to-weight ratio makes them well-suited engineering substrates for implementing bird-inspired morphing architectures at flight-relevant structural loads [129,130]. Shahid et al. [9] reported endurance improvements of up to 30% from aerodynamic shape adaptation and identified flexible composite wing materials as multi-role substrates capable of simultaneously providing structural load-bearing, aerodynamic shape adaptation, and energy-conversion substrate functions.

The central engineering challenge of E-1 is a multi-objective co-design problem: the structural element must simultaneously deliver load-bearing stiffness, elastic morphing compliance, surface compatibility for TENG or photovoltaic bonding, and a mass budget consistent with UAV endurance targets. Ajaj et al. [131] systematically documented the aeroelastic dimension: morphing degrees of freedom simultaneously alter aerodynamic load distributions, structural stiffness, and inertial properties, producing coupled changes in flutter speed, divergence onset, and gust response that cannot be predicted from the non-morphing baseline. Any TENG or photovoltaic coating modifying skin stiffness without concurrent multi-physics aeroelastic reanalysis represents an uncharacterised structural safety gap in all current integration studies, a critical prerequisite for flight-ready E-1 realisation. Resolving this barrier will require co-simulation frameworks coupling morphing aerodynamics, composite structural dynamics, functional layer mechanics, and aeroelastic response within a unified design environment.

### 7.7. Cross-Domain Engineering Barriers

Four systemic barriers span multiple mechanisms and require coordinated resolution:

B1: Mass–Energy Balance. All functional additions (TENG and PV films, neuromorphic processors, bistable piezoelectric structures) impose mass penalties potentially offsetting energy gains. Net system-level endurance analyses simultaneously accounting for aerodynamic drag changes, gravitational energy costs, and conversion efficiency losses remain absent across all six mechanisms. Zheng et al. [13] constitute a partial exception by reporting both mass increase (+7%) and harvested power output in the same study.

To provide a common assessment framework, this review proposes a mission-level net mass–energy benefit index, $B_{\mathrm{ME}}$, defined as

(6) $$B_{\mathrm{ME}}=\frac{E_{0}-E_{\mathrm{bio}}}{E_{0}}$$

where $E_{0}$ is the onboard primary energy required by the baseline UAV to complete a specified reference mission, and $E_{\mathrm{bio}}$ is the net onboard primary energy required by the bio-inspired configuration to complete an equivalent reference mission under the same payload, mission objective, and environmental boundary conditions. $E_{\mathrm{bio}}$ should include propulsion, actuation, control, and auxiliary energy demands while crediting only harvested or recovered energy that is usable after conversion and storage losses and that would otherwise have been supplied by the primary energy source. The energetic penalty associated with added mass Δm should be evaluated through a vehicle-specific propulsion model rather than by mass alone, because UAV propulsion-energy requirements depend on carried mass and operating conditions, and the resulting energy estimates vary with vehicle configuration and model formulation [132,133].

(7) $$E_{\Delta m}=\int_{0}^{T}\;\left[P_{\mathrm{prop},\mathrm{bio}}\left(m_{0}+\Delta m,t\right)-P_{\mathrm{prop},\mathrm{bio}}\left(m_{0},t\right)\right]\;dt$$

where $P_{\mathrm{prop},\mathrm{bio}}\left(m,t\right)$ denotes the propulsion-power model for the same bio-inspired configuration, evaluated at different vehicle masses. All non-mass characteristics of the bio-inspired design should, therefore, be held fixed when evaluating $E_{\Delta m}$, so that this term isolates the energetic contribution of added mass rather than mixing the mass penalty with aerodynamic or functional benefits. For small Δm, this may be approximated by

(8) $$E_{\Delta m}\approx \int_{0}^{T}\;{\left.\frac{\partial P_{\mathrm{prop},\mathrm{bio}}}{\partial m}\right|}_{m=m_{0}}\Delta m\;dt$$

$E_{\Delta m}$ is a diagnostic quantity used to isolate the contribution of added mass to the modified mission-energy balance. It must not be added separately to $E_{\mathrm{bio}}$ when the propulsion-energy calculation for $E_{\mathrm{bio}}$ already evaluates the modified vehicle at $m_{0}+\Delta m$.

Under this framework, $B_{\mathrm{ME}}>0$ denotes a net-positive mission-energy benefit, $B_{\mathrm{ME}}=0$ the break-even condition, and $B_{\mathrm{ME}}<0$ a net energy penalty. For a specified bio-inspired configuration and reference mission, the primary criterion for energetic favourability is $B_{\mathrm{ME}}>0$. If $B_{\mathrm{ME}}$ decreases monotonically with added mass and a unique zero crossing exists, a critical added mass $\Delta m_{\mathrm{crit}}$ can be defined by

(9) $$B_{\mathrm{ME}}\left(\Delta m_{\mathrm{crit}}\right)=0$$

Under this condition, an actual added mass below $\Delta m_{\mathrm{crit}}$ corresponds to a net-positive mission-energy benefit. If monotonicity or a unique zero crossing is not present, $B_{\mathrm{ME}}$ itself should be used as the governing criterion rather than $\Delta m_{\mathrm{crit}}$.

For endurance-oriented comparisons, the same vehicle-level balance can be expressed through the usable onboard primary-energy capacity $E_{\mathrm{use}}$ and the net primary-power demand $P_{\mathrm{primary}}\left(t\right)$. In Equations (10)–(12), $P_{\mathrm{primary}}\left(t\right)$ denotes the complete vehicle-level net primary-power demand used in this mission-level framework; Equation (5) should be interpreted only as a reduced two-load illustration retaining the propulsion and avionics terms needed to analyse C-3. Equation (11) restates the first-order endurance relation introduced in Equation (3) using the notation of the present mission-level framework. The endurance $T_{\mathrm{end}}$ is determined by

(10) $$\int_{0}^{T_{\mathrm{end}}}\;P_{\mathrm{primary}}\left(t\right)\;dt=E_{\mathrm{use}}$$

which can equivalently be written as

(11) $$T_{\mathrm{end}}=\frac{E_{\mathrm{use}}}{{\bar{P}}_{\mathrm{primary}}}$$

when the mission is represented by its mission-averaged net primary-power demand. Baseline and bio-inspired configurations should therefore be evaluated under comparable operating conditions using their respective $E_{\mathrm{use}}$ and $P_{\mathrm{primary}}\left(t\right)$. An endurance improvement is obtained only when

(12) $$\frac{E_{\mathrm{use},\mathrm{bio}}}{{\bar{P}}_{\mathrm{primary},\mathrm{bio}}}>\frac{E_{\mathrm{use},0}}{{\bar{P}}_{\mathrm{primary},0}}$$

so that changes in usable onboard energy capacity and net power demand are evaluated simultaneously rather than in isolation.

This framework is proposed in this review as a system-level comparison metric and is not presented as an experimentally validated universal vehicle model. Accordingly, $P_{\mathrm{prop},\mathrm{bio}}\left(m,t\right)$ must be evaluated using a propulsion model appropriate to the UAV configuration and mission.

B2: Operational Durability. Performance retention of TENG-integrated morphing surfaces over 10^4^–10^6^ operational bending cycles at flight-relevant frequencies is undocumented. The bistable architecture of Wu et al. [107] reduces stress concentration and represents progress towards this gap, but systematic fatigue characterisation under realistic flight-spectrum loading profiles remains absent. Bio-inspired nanostructures for C-5 have not been assessed for UV photodegradation, high-velocity particle impact, moisture ingress, or cyclic thermal loading at altitude.

B3: Aeroelastic Co-Design. As systematised by Ajaj et al. [131], morphing wings alter aerodynamic load distributions, structural stiffness, and inertial properties simultaneously, producing coupled changes in flutter boundaries and gust load responses unpredictable from the non-morphing baseline. TENG or PV coatings modifying skin stiffness without concurrent multi-physics aeroelastic reanalysis constitute an uncharacterised structural risk in E-1, C-2, and C-5.

B4: Standardised Performance Metrics. Heterogeneous testing conditions impede cross-study comparison: aeroacoustic noise reductions are measured at varying microphone placements, RPM points, and advance ratios [4]; energy harvesting power densities are normalised inconsistently per mass, area, or volume; neuromorphic efficiency metrics alternate between inference-level and system-level measurements. UAV-specific standardised benchmarks are a prerequisite for the quantitative cross-coupling evaluation necessary to design multi-mechanism integrated platforms.

### 7.8. Dynamic Similarity and Scaling Constraints

Dynamic similarity provides an additional constraint on transferring biological mechanisms to UAV platforms [20]. Reynolds number (Re) governs the relative importance of inertial and viscous effects and is particularly important for fixed-wing and tubercled-surface transfer; as discussed in Section 2.3, biological humpback-whale flow occurs at approximately $Re∼10^{6}$, whereas small-UAV/MAV operation commonly occupies the lower $Re∼10^{4}$–$10^{5}$ regime represented in the reviewed evidence [20,21]. For unsteady flapping or oscillatory mechanisms, Strouhal number (St) and reduced frequency (*k*) describe the timing of motion relative to the convective flow scale, while aspect ratio (AR) influences finite-wing vortex development and induced effects. Rotating bio-inspired propellers additionally require consideration of advance ratio (*J*), and rotational or revolving-wing concepts can depend on Rossby number (Ro) through the relative importance of rotational effects. These parameters are not interchangeable: matching Re alone does not guarantee similarity when the underlying mechanism depends on unsteady, rotational, or finite-span aerodynamics. Consequently, biological geometries should be transferred by preserving the nondimensional parameters governing the target mechanism wherever possible, followed by validation under the Reynolds number, operating condition, and vehicle-scale constraints of the intended UAV.

To consolidate these scale-transfer constraints, Table 10 compares the principal nondimensional parameters, representative operating regimes, and transfer limitations for the aerodynamic strategies in which dynamic similarity is particularly important. Where the reviewed literature does not establish a universal nondimensional performance window, the table explicitly identifies the range as study-specific rather than extrapolating a common threshold across heterogeneous configurations.

### 7.9. Synergy Potential Matrix and Strategic Research Agenda

The hierarchical dependency structure defines a sequential strategic programme rather than parallel independent development tracks. C-1 warrants immediate implementation: it is experimentally grounded, requires no additional subsystem integration beyond propeller geometry modification, and provides the most direct path from bio-inspired design to commercial UAV performance improvement. C-4 maturation is a prerequisite for C-3: DS and V-formation coordination demand high-bandwidth real-time environmental sensing and adaptive trajectory planning for which neuromorphic event-based processing is architecturally suited. E-1 is the structural enabling prerequisite for C-2 and C-5: advances in multifunctional morphing composite design, aeroelastic co-analysis, and multi-layer fabrication are required before C-2 and C-5 can be co-located on the same flight-ready aerodynamic surface. Table 11 consolidates the six mechanisms in terms of coupling type, quantitative evidence, evidence level, principal barrier, and strategic priority.

The collective implication is that biomimetic UAV research must transition from domain-specific optimisation of individual bio-inspired features to functional, system-level integration of complementary technologies spanning aerodynamic, structural, control, and energy domains. The mechanisms characterised here are not additive improvements: they define configurations in which technologies reinforce one another’s effects, potentially producing system-level performance gains beyond those achievable through isolated optimisation of any single technology. The pathway from near-term propulsion improvement through C-1 to the long-horizon possibility of energy-autonomous flight through the convergence of C-3, C-4, and C-5 represents the strategic continuum of this research agenda. Specific future research directions are developed in Section 8.

The synergy matrix above identifies evidence maturity and strategic priority, but does not explicitly show where subsystem benefits are accompanied by penalties elsewhere in the vehicle. Table 12 therefore provides a qualitative interaction matrix in which positive, negative, negligible, and operating-condition-dependent effects are displayed simultaneously. The entries are an engineering synthesis of the evidence reviewed in Section 2, Section 3, Section 4, Section 5, Section 6 and Section 7 rather than universal quantitative ratings.

Table 13 complements the interaction analysis by comparing representative technologies across the six principal domains in terms of reported performance, integration burden, and UAV readiness.

Taken together, Table 10, Table 11, Table 12 and Table 13 can also be read as a mission-conditioned prioritisation aid rather than as a universal ranking. For short-duration multirotor inspection missions, near-term priorities include passive propulsion improvements such as C-1 and low-power sensing and control options because propulsion and avionics directly draw from the onboard energy budget. For long-endurance fixed-wing missions, dynamic-soaring or formation-flight strategies and lightweight photovoltaic integration become more relevant where atmospheric, geometric, and operational conditions permit, whereas MAV-scale platforms are better matched to flapping/LEV mechanisms and low-power wing-integrated harvesting. These examples are illustrative and should be filtered through the dynamic-similarity limits in Table 10, subsystem interactions in Table 12, and readiness evidence in Table 13.

## 8. Conclusions

This review has shown that the principal performance constraints of electrically powered UAVs (flight endurance, aerodynamic efficiency, and acoustic emissions) are not independent engineering problems but physically and functionally coupled challenges, each mediated by the shared onboard energy budget. Bio-inspired technologies were examined across the principal domains of aeroacoustic and drag-reduction surface design, aerodynamic efficiency, structural materials and multifunctional composites, sensing and neuromorphic control, ionic energy storage, and energy harvesting. These domains were organised through a cross-domain synergy analysis that treats bio-inspired technologies as potentially interacting components of a shared engineering architecture rather than as isolated biological analogues.

Many previous UAV biomimetics reviews have tended to categorise technologies by biological inspiration or engineering function, whereas the present review organises them through cross-domain functional coupling. This framework identifies a shared origin of aeroacoustic noise and propulsive inefficiency in coherent vortex structures at the blade surface. Accordingly, bio-inspired serration geometries can simultaneously address both penalties, as demonstrated by experimental UAV platform studies. Similarly, morphing wing surfaces that improve aerodynamic adaptability constitute deformable substrates for mechanical energy harvesting, and neuromorphic event-driven architectures that substantially reduce avionics power consumption directly relieve the energy budget constraining every other onboard subsystem. These are not additive improvements; they define configurations in which bio-inspired technologies reinforce one another’s effects, potentially yielding system-level performance gains beyond those achievable through isolated optimisation of any single technology.

Among the reviewed pathways, aeroacoustic–propulsive coupling and neuromorphic perception appear closest to near-term UAV implementation. Bio-inspired propeller serration geometries have been experimentally validated on quadrotor platforms under representative hover conditions, and neuromorphic processors have demonstrated UAV-relevant autonomous perception and control tasks with substantially lower power consumption than conventional graphics processing unit (GPU)-based implementations. By contrast, morphing-integrated mechanical energy harvesting and bio-inspired ionic-gradient storage represent longer-term directions: harvested power levels from integrated triboelectric and piezoelectric devices remain insufficient for propulsion-scale contributions, and ionic-gradient electrochemical architectures inspired by the electric organ of the electric eel have not yet demonstrated energy densities sufficient for UAV propulsion requirements. Bio-structured photovoltaics, including quasi-two-dimensional perovskite devices demonstrated on ultralight UAV platforms, represent one of the most practically advanced bio-inspired energy technologies reviewed. However, their integration at operationally representative scales remains an open manufacturing challenge.

Several cross-cutting barriers currently impede the transition from domain-specific bio-inspired innovation to integrated multi-mechanism UAV platforms. Material durability under UAV operational conditions remains largely uncharacterised for flexible photovoltaic laminates and triboelectric composite structures. The net mass–energy balance of any multi-technology integrated configuration has not been quantified at the full system level. Aeroelastic co-design represents a structural risk whenever energy-harvesting or photovoltaic coatings alter wing-skin stiffness without concurrent multi-physics reanalysis. Finally, the absence of standardised UAV-specific performance metrics (governing aeroacoustic measurement conditions, energy harvesting power density normalisation, and neuromorphic efficiency reporting basis) constitutes a methodological prerequisite for the quantitative cross-domain comparison that integrated platform design requires.

Future research should proceed hierarchically: near-term implementation of validated propeller geometries and neuromorphic perception should be followed by medium-term development of multifunctional morphing composites as the structural prerequisite for co-located energy harvesting and photovoltaic integration. Standardised UAV-specific performance benchmarks are a cross-cutting prerequisite for this progression. The long-term horizon of energy-autonomous UAV operation depends on convergence between atmospheric energy harvesting strategies and advances in bio-inspired electrochemical storage that remain at early research stages.

The overarching implication of this review is that the most consequential advance available to biomimetic UAV research is conceptual: a transition from collecting isolated biological analogies towards deliberately engineering systems in which aeromechanical, structural, sensing, control, and energy technologies function as interacting components of a shared, energy-constrained aerial architecture. Biological flyers achieve aerodynamic adaptability, energetic economy, and acoustic efficiency not through individually optimised subsystems, but through co-evolved integration in which each component’s performance enhances that of others. The future of biomimetic UAV design lies not in reproducing isolated biological structures, but in translating these integrated, co-optimised functional principles: a systematic transition from structural imitation towards functional, system-level biomimetics.

## Figures and Tables

**Table 1:** Comparative summary of bio-inspired passive flow-control strategies reviewed in Section 2.

Strategy	Flow-Control Mechanism	Biomimetic Classification	Key Bench-Scale Evidence	UAV Evidence Context	Critical Integration Constraint	Ref.
LE serration	Aeroacoustic source disruption; laminar-turbulent transition modification	Direct functional biomimicry	Noise reduction (sawtooth: mean 2.43 dB, max 4.18 dB; 3.53% thrust gain; UAV hover: 4.73 dB at 5 m, 3.79 dB at 8 m)	CFD + experiment; UAV propeller; $Re\approx 10^{4}$–$10^{5}$	Condition-dependent aerodynamic impact; independent replication limited; no mission-envelope validation	[19]
LE+TE BCP	Combined LE disruption + TE scattering attenuation	Direct functional biomimicry	Lab: max 3.1 dB, 7.9% force-to-speed ratio ($I_{g}$) improvement; outdoor hover: 3.8 dB (3 m), 3.3 dB (6 m), 2.6 dB (12 m)	Experiment + CFD; single geometry; outdoor hover confirmed; $Re\approx 1.5\times 10^{5}$	Power consumption increases at fixed RPM; mechanism unconfirmed in rotating frame	[5]
3D-SC (owl + cicada)	Integrated serration + bio-derived planform	Mixed: direct biomimicry (serration) + geometric abstraction (planform)	Max 5.5 dB noise reduction (far-field, 5 m, 30°); 20.2% power reduction vs. DJI Phantom 3; 6-inch prototype	Rotor bench; specific operating conditions; 6-inch scale	Planform Re-regime transfer unvalidated; 6-inch constraint; advance-ratio envelope uncharacterised	[6]
Tubercles	CRVP-induced BL energisation; stall delay	Morphology-inspired adaptation (aquatic → aerodynamic)	Performance gain confined to high wind speed; low-speed (low-incidence) performance penalty; benefit governed by A/λ ratio and outer 60% blade-span coverage; root backflow elimination at rated wind speed	Low-Re experimental and numerical airfoil evidence (Re≈1.0–$1.2\times 10^{5}$) [20,21]; HAWT RANS, $V_{\infty }=7$–25m/s [22]	Pre-stall drag and low-speed performance penalties; no rotating-hardware UAV validation	[20,21,22]
Riblets	Turbulent sublayer vortex displacement; skin-friction drag reduction	Bio-derived engineering abstraction (aquatic → aeronautical)	Up to 21.45% skin-friction reduction (LES, pipe flow, Re=40,459); drag increase outside $s^{+}$ optimum	LES + pipe/channel flow; no aeronautical rotor data	Narrow, regime-dependent $s^{+}$ optimum; contamination susceptibility; non-aeronautical evidence base	[23]

**Table 2:** Comparison of avian morphing wing degrees of freedom: biological model, primary aerodynamic benefit, quantified performance, and key engineering limitation.

Morphing DoF	Biological Model	Primary Aerodynamic Benefit	Quantified Performance	Key Engineering Limitation
Wing Sweep (20–60°)	Gull; Peregrine Falcon	Induced drag reduction; longitudinal stability and L/D control	↓ 18% drag [9];	Actuator mass penalty (payload fraction −6 pp, 24% → 18% [9]); coupled stability shifts
Span Extension	Common Swift (*Apus apus*)	Cruise drag reduction; range extension at constant power	+40% span → ∼15% fuel saving [9]; FishBAC camber morphing: L/D ↑ 20–25% vs. hinged flap (wind-tunnel validated) [35]; modular design ΔCL ≈ 0.55 [36]	Structural integrity under dynamic loading; folding mechanism complexity
Wing Dihedral Variation	Wandering Albatross; Gull	Lateral–directional stability; gust-load alleviation; roll control	Dynamic dihedral variation (−50° to −5°) increases DS energy gain by 25.43% and reduces minimum wind-shear gradient by 15.56% relative to fixed −5° [37]; see also Table 3	Nonlinear roll–yaw coupling; sensor-feedback dependency
Camber/Twist Morphing	Pigeon (*Columba livia*); Owl	Global L/D improvement; local lift distribution tailoring; roll control	SMA camber ±8° → L/D +18% [9]	SMA hysteresis; temperature-dependent response; fabrication cost
Alula-Inspired Leading-Edge Device	Peregrine Falcon; Eagle	Stall delay; sustained lift at high AoA; low-speed manoeuvring enhancement	Stall speed ↓ 20% [9]	Integration with main wing structure; passive vs. active deployment trade-off

**Table 3:** Overview of bio-inspired aerodynamic efficiency strategies reviewed in Section 3.2, Section 3.3 and Section 3.4: operating principle, quantified benefit, and current engineering barrier.

Strategy	Biological Model	Operating Principle	Quantified Benefit	Current Engineering Barrier
DS	Wandering Albatross (*Diomedea exulans*)	Wind-shear energy extraction; sustained low-power cruise in atmospheric boundary-layer gradient	Energy-neutral flight in simulation; minimum wind speed ≈5 m/s under the modelled conditions [38]	Inherent trajectory instability [38]; wind-condition dependency; small-UAV scaling experimentally unvalidated
V-Formation/Wake Recovery	White Pelican; Northern Bald Ibis; Migratory Geese	Upwash capture from leader wingtip vortices; induced drag reduction for trailing members	Range increase up to 71% (theory, 25-bird V) [39]; heart-rate ↓11.4–14.5% [40]; MALE UAV L/D +20–39% [41]	Narrow beneficial spacing band; inter-UAV communication latency; reduced upwash gain at small wingspan scale
LEV Exploitation (Flapping-Wing MAV)	Dragonfly; Hawkmoth; Hummingbird; Butterfly	Delayed stall; rotational lift; wake capture; clap-and-fling: sustained high-AoA lift at low Re	Tandem-wing: aerodynamic force up to 4.3× body weight [11]; sub-gram MAV $C_{L}\approx 1.6$ [9]	Re scaling gap ($10^{2}$–$10^{3}$ vs. $10^{4}$–$10^{5}$) [11,42]; LEV breakdown at larger scale; flexible micro-scale wing fabrication

**Table 4:** Overview of bio-inspired structural and multifunctional technologies reviewed in Section 4.1.

Technology	Biological Archetype	UAV Application	Key Performance Metric	Section 7 Coupling
Avian-inspired hollow spar design	Avian pneumatised bone; trabecular strut hierarchy	Lightweight fuselage/wing beam	Improved stiffness-to-mass performance relative to solid or non-hollow load-bearing designs; quantitative gains remain geometry- and manufacturing-dependent	N/A
Gecko-inspired dry adhesive (SSA)	Gecko setae; hierarchical fibrillar adhesion with sensing analogy	UAV perching; low-power station-keeping	Real-time adhesion-force and adhesion-state monitoring; supports perched observation by reducing reliance on continuous thrust on suitable surfaces [57,58]	N/A
SMA morphing actuator (NiTi)	Avian musculoskeletal antagonist muscle pairs	Large-camber morphing wing rib/trailing-edge reconfiguration	Large-stroke morphing actuation potential; constrained by thermally activated response, cooling latency, and auxiliary power demand [59]	C-2
Piezoelectric MFC actuator	Strain-sensitive mechanoreceptors/campaniform-sensilla analogy	High-frequency vibration suppression, small-amplitude trim, SHM dual function	Fast, small-amplitude actuation suitable for vibration suppression and trim adjustment; simultaneous sense–actuate capability via the direct piezoelectric effect [59]	C-2
Electroactive polymer (EAP) wing skin	Compliant biological muscle/soft-tissue actuation	Soft morphing wing skin; artificial muscle for compliant surfaces	Compliant deformation for soft morphing surfaces; constrained by high driving voltage, durability, fatigue, and integration challenges [9,59]	C-2
Structural battery composite (SBC)	Bone-like multifunctional load-bearing mineralised tissue	Wing/fuselage skin integrating Li-ion storage and structural load path	Up to +41% projected hover-time improvement for structural-battery-integrated UAV concepts [60]; VARTM-fabricated carbon-fibre SBCs reported 41.2 Wh kg^−1^ and 80% lower charge-transfer resistance [61]; no open-literature UAV in-flight validation yet	N/A
Morphing skin as TENG/PENG substrate	Mechanically responsive biological surfaces/strain-sensitive skins	Dual-function aerodynamic surface and triboelectric/piezoelectric harvesting substrate	Gust- or morphing-induced deformation provides mechanical input for electrical generation through contact electrification or strain-induced polarisation; output depends on deformation mode and integration conditions	C-2

C-2 = morphing wing deformation–triboelectric/piezoelectric physical coupling; N/A indicates technologies whose primary coupling falls outside the C-1–C-5 framework formalised in Section 7.

**Table 5:** Overview of bio-inspired sensing systems reviewed in Section 4.2.

Technology	Biological Archetype	UAV Application	Key Performance Metric	Section 7 Coupling
Compound-eye camera (BM3C)	Insect compound eye; curved ommatidial array	Wide-field aerial multispectral imaging; ground-object classification	169 multispectral ommatidia on a curved shell; tested FOV 127.4°; seven spectral channels across nominal 500–800 nm; 13 fps; optical distortion below 2%; further size and mass reduction required for broader small-UAV deployment [63]	N/A
Polarisation navigation sensor	Polarisation-sensitive dorsal rim region of insect compound eyes	Artificial-signal-independent heading estimation for mobile robotic and UAV platforms	Miniaturised sensor with 15 g mass and 4 × 4 × 2 cm envelope; indoor and outdoor calibration accuracies of ±0.3° and ±0.5°, respectively [64]	N/A
Artificial hair sensor/Flight-by-Feel	Fish lateral-line neuromasts; arthropod trichoid hairs; avian/bat flow-sensitive hairs	Distributed wing-surface flow sensing; aerodynamic-state awareness; stall, separation, and gust-related sensing	AHSs provide local airflow, flow-direction, incident-angle, and laminar/turbulent-flow information; FBF uses distributed pressure, strain, and flow sensors to improve real-time aerodynamic-state awareness [65]	N/A
Sparse sensor placement for Flight-by-Feel	Non-uniform biological sensor distributions in lateral-line and hair-cell systems	Minimal-sensor FBF under SWaP constraints	Sparse placement aims to reduce sensor count, lag, SWaP cost, and integration complexity while preserving aerodynamic-state reconstruction capability; performance remains problem- and configuration-dependent [65,71]	N/A
Embodied airflow sensing for FWMAVs	Wing-integrated mechanosensory hairs in insects, birds, and bats	Structural deformation or wing-surface sensing as input for flapping-wing control	Embodied sensing can use distributed wing or membrane deformation as a flow/gust-related signal, potentially reducing reliance on separate protruding transducers; validation remains platform-specific [65,72]	N/A
Neuromorphic event camera	Biological retina; asynchronous per-pixel luminance-change encoding	High-speed visual odometry, obstacle avoidance, optical flow, and UAV navigation	Microsecond-scale temporal resolution, low latency, reduced data redundancy, low power consumption, and high dynamic range of approximately 140 dB versus about 60 dB for conventional frame cameras; UAV studies span datasets, simulators, algorithms, applications, and future directions [66]	C-4

Section 7 coupling label: C-4 = neuromorphic computation–avionics power budget compatibility coupling. N/A indicates technologies whose primary coupling falls outside the C-1–C-5 framework formalised in Section 7.

**Table 6:** Overview of bio-inspired autonomous control architectures reviewed in Section 4.3.

Technology	Biological Archetype	UAV Application	Key Performance Metric	Section 7 Coupling
Bio-inspired multi-UAV path planning	Swarm foraging, ant-colony pheromone search, bird/flock coordination analogies	Multi-UAV trajectory optimisation; task allocation; route planning	Bio-inspired algorithms, including PSO, GA, GWO, ACO, PIO, FOA, LSO, and IWD are widely used for large, constrained, and uncertain multi-UAV path-planning problems; performance is problem-dependent rather than universally superior [67]	C-4
Swarm-intelligence and hybrid BIA–AI coordination	Collective animal motion; decentralised group decision-making	Formation management, inter-UAV coordination, mission planning, and energy-aware route allocation	Trajectory-design reviews cover traditional, bio-inspired, and modern AI-based approaches; energy expenditure can be reduced when formation geometry, task allocation, coordination, and total flight distance are jointly considered [68]	C-4
Fully neuromorphic SNN vision-to-control	Insect-like visual–motor coupling; sparse asynchronous spiking computation	End-to-end autonomous vision-based hovering, landing, and lateral manoeuvring	On-board Loihi-based pipeline operated at 200 Hz and was reported to spend 27 μJ per network inference; separate offline Nahuku-board benchmark measured 274–1,637 Inf/s and approximately 7.2–27.2 μJ per inference, corresponding to three to four orders of magnitude lower inference energy than a Jetson Nano platform comparison [14]	C-4
SNN attitude estimation and control	Sparse spiking sensorimotor computation; recurrent neural control analogy	Low-level attitude estimation and control on a Crazyflie nano-UAV	Onboard SNN ran at 500 Hz on a microcontroller, achieving 3.03° attitude-tracking RMSE; 15% spiking activity suggests potential energy-efficiency benefits for future neuromorphic implementation [69]	C-4
Bio-inspired neural decision-making and neuromodulatory motor control	Cortical short-term memory, winner-take-all decision circuits, basal-ganglia-inspired modulation, nonlinear motor oscillators	Transferable robotic decision-making and motor-control template; not yet UAV-validated in [70]	Differential-drive ground-robot study demonstrated obstacle avoidance and exploratory behaviour using short-term memory, WTA, neuromodulatory, and nonlinear oscillation circuits; UAV relevance remains conceptual/system-level [70]	C-4
ANN/RL-assisted adaptive flight control	Functional analogy to biological sensorimotor adaptation, rather than mechanistic replication	Robust control of flapping-wing and bio-inspired UAVs under nonlinear dynamics, uncertainty, disturbance, and wing-damage conditions	Reviewed strategies include SMC, adaptive control, NN-assisted control, NN-based adaptive SMC, and ASMC with reinforcement learning; direct evidence for propulsion-energy reduction or endurance extension remains limited [7]	C-4

Section 7 coupling label: C-4 = neuromorphic computation–avionics power budget compatibility coupling.

**Table 7:** Comparison of UAV energy storage and bio-inspired ionic energy-conversion technologies. Systems are categorised by UAV integration role.

Technology	Biological Model	Key Performance Metrics	Gravimetric Energy Density	UAV Integration Category	Refs
LiPo battery (baseline)	None	150–250 Wh/kg; 20–40 min endurance	150–250 Wh/kg (pack level)	Current standard	[1,2,73]
PEM H_2_ fuel-cell hybrid	None	250–540 Wh/kg system; 62–70 min demonstrated; 331 min record under optimised conditions	250–540 Wh/kg (system)	Demonstrated in ≥5 kg class	[74,76]
Hydrogel/solid-state ionic-gradient battery	*Electrophorus electricus*/*E. voltai* electric organ	Up to 110 V and 27 mW $m^{-2}$ [78]; ∼1300 W $m^{-3}$ [79]; $V_{\mathrm{OC}}∼$135 mV/unit [80]	Not yet benchmarkable against LiPo; insufficient evidence for propulsion-scale energy density	Research stage: conformal auxiliary power; long-term structural integration hypothesis	[16,79,80,81,82,83,84,85,86]
RED/osmotic nanofluidic membrane	Fish gill; bio ion channels	1.47–67 W $m^{-2}$ under salinity gradients; direct onboard storage not applicable	Not applicable (energy conversion, not storage)	Infrastructure concept: coastal/maritime UAV charging	[87,88,89,90,91,92,93,94]

Notes: the 331-min PEM-FC flight was obtained under highly optimised conditions and is not representative of typical multirotor operation. Ionic-gradient Wh/kg values have not yet been reported; comparisons with LiPo must remain qualitative. RED/osmotic systems require continuous external salinity supply and are, therefore, categorised as energy-conversion infrastructure rather than onboard storage.

**Table 8:** Comparison of bio-inspired photovoltaic and solar-harvesting technologies for UAV integration.

Technology	Biological Inspiration	Peak Performance	Primary Barrier	UAV Integration Status
Disordered nanohole thin-film PV (a-Si:H)	*P. aristolochiae* wing-scale aperiodic nanostructure	+93% integrated absorption increase at normal incidence (450–800 nm); +207% at 50° AOI [99]	UV/moisture degradation; curved-surface deposition uniformity	Lab-validated; wing-surface integration not yet demonstrated
Flexible quasi-2D PSC module	Solar-harvesting benchmark; functionally analogous to biological light harvesting	Specific power 44 W/g (avg. 41 W/g); PCE 20.1%; energy-autonomous 13 g quadcopter demonstrated [101]	Operational stability under prolonged UV; large-area scale-up	Quadcopter drone flight demonstrated
Flexible OSC module (LITO electrode)	Flexible PV benchmark for UAV integration; not intrinsically biomimetic	PCE 17.12% (1 cm^2^); 15.60% (42 cm^2^); 97% retention after 1000 bending cycles; +24.2% SUAV endurance [102]	Scale-up consistency; encapsulation under outdoor flight conditions	SUAV prototype demonstrated
Bio-inspired multiscale perovskite PV	Butterfly wing/moth-eye/leaf hierarchical nanostructures	Structural-disorder design principle validated; PCE approaching record levels [100]	Long-term outdoor humidity and UV stability	Concept stage; structural wing integration undemonstrated
Battery-free solar fixed-wing UAV	Plant photosynthesis (indirect functional analogy)	Full flight cycle on solar power only; capacitive storage [103]	Night operation impossible; cloud-cover vulnerability	Prototype fixed-wing UAV flight demonstrated

**Table 9:** Comparison of bio-inspired mechanical energy-harvesting technologies for UAV integration.

Technology	Biological Inspiration	Peak Performance	Primary Barrier	UAV Integration Status
H-TENG (hummingbird-wing, flutter-driven)	Hummingbird wing flutter mechanics	1.5 W/m^2^ at 7.5 m/s (6-unit network); device mass 10 g [106]	Frequency matching to operational UAV flight regime	Benchtop wind-tunnel validated; UAV mounting not demonstrated
h-TENG (hierarchical honeycomb, dual-mode)	Honeycomb cellular mechanics (bending + compression)	Morphing-wing test: maximum output power of 275 μW at 30° flap deflection, 2 Hz excitation, and 21 MΩ load resistance;separate hand-tapping benchmark: 1207 V, 68.5 μA, 12.4 mW, corresponding to 0.275 mW cm^−3^ or 2.48 mW g^−1^ [12]	Output density vs. UAV power budget	Morphing-wing substrate integration feasibility demonstrated
Bistable PENG–seagull wing (S-type)	Seagull two-phase wingbeat cycle	0.734 mW at 18 Hz; 11.5 V open-circuit [107]	Performance drop above resonant frequency	Benchtop; flapping-wing UAV frequency compatibility pending
Bistable PENG–seagull wing (M-type)	Seagull wingbeat; bionic M-shape stress reduction	11.5 V open-circuit; 73 μW peak; magnet-free; reduced fatigue [108]	Output power under real flight vibration conditions	Lab prototype; UAV integration not yet demonstrated
Aerofoil-based aeroelastic piezo harvester	Avian wing aeroelastic flexibility (functional analogy)	Stable LCO-based harvesting from 4 m/s; milliwatt-range peak outputs [109,110]	Broadband excitation; structural integrity under prolonged fatigue cycling	Wind-tunnel and field-validated; UAV integration not yet demonstrated
TENG composite FWMAV wing (silk-fibroin layer)	Biomimetic FWMAV wing + silk-fibroin triboelectric material	468.7 V; 100.6 μA; 379.8 μW; +29.89% onboard load capacity [13]	Micro-scale output insufficient for propulsion	Integrated FWMAV; self-powered sensing demonstrated
Silk-protein TENG + FAV (ESCM framework)	Silk-fibroin triboelectric material	40.5 ± 2.2 mW avg/100 h; +36.82% vs. control; 93.5% energy recovery accuracy [112]	Output vs. propulsion demand (tens–hundreds of W)	Directly integrated into FAV; 100 h flight-simulated validation
PVDF transducers on multirotor UAV arm	Functional biomechanical harvesting	17.3 V at 3975 rpm; capacitor charged in ∼162 s [113]	Supplementary subsystem power only; no propulsion contribution	Integrated multirotor UAV; flight-test validated

**Table 10:** Dynamic-similarity considerations and scale-transfer limitations for representative bio-inspired aerodynamic strategies.

Bio-Inspired Strategy	Governing Non-Dimensional Parameter(s)	Representative Regime / Effective Condition in Reviewed Evidence	Implication for UAV-Scale Transfer
Humpback-whale leading-edge tubercles	Re; A/λ	Biological source: $Re∼10^{6}$; UAV/MAV-relevant evidence: $Re=1.0\times 10^{5}$ and $1.2\times 10^{5}$, with comparison at $1.5\times 10^{6}$ [20,21,26].	Vortical mechanism can persist at lower Re, but lift, stall, separation, and reattachment characteristics change. Geometry, therefore, requires target-Re validation rather than direct geometric scaling.
Shark-skin-inspired riblets	Re; $s^{+}$; $A_{g}^{+1/2}$	Maximum drag reduction near $s^{+}∼15$; $A_{g}^{+1/2}∼10.7$, with deterioration beyond the narrow optimum range [29].	Performance depends on local wall-shear conditions. A riblet geometry optimised for one speed or surface location may move outside its productive range elsewhere on the UAV.
Avian-inspired morphing wings	Re; AR; nondimensional geometric morphing ratios	Reviewed morphing-wing studies include $Re=2.7\times 10^{5}$, $3.2\times 10^{5}$, and $4.05\times 10^{5}$ [44,45].	Morphing benefit is Reynolds-, geometry-, and flight-condition-dependent. Biological morphology or a laboratory configuration should not be transferred across UAV scales without aerodynamic re-optimisation.
Albatross-inspired dynamic soaring	Re; AR; wind-shear/vehicle-dynamic scaling	Energy-neutral operation is conditional on the available wind field; reviewed evidence reports a minimum wind speed of approximately 5 m s^−1^ for sustained dynamic soaring under the specific conditions considered in [38].	No single universal nondimensional operating window is established in the reviewed UAV literature. Transfer is constrained by atmospheric shear, wing loading, trajectory dynamics, and vehicle scale.
V-formation/wake recovery	AR; normalised inter-vehicle spacing relative to wingspan	Benefit occurs only within a comparatively narrow relative-positioning region; reviewed MALE-UAV studies report L/D improvements of 20–39% under specific formation conditions [41].	Geometric normalisation by wingspan is necessary but insufficient. Wake structure varies with Re, AR, loading, and spacing, and the concept is principally applicable to fixed-wing cruise rather than multirotors.
Flapping-wing/LEV exploitation	Re; St; Ro; *J*; *k*; AR	Representative biological and MAV regimes reviewed here include $Re=10^{2}$–$10^{5}$; LEV stabilisation is associated with Ro≤4; AR∼3–4 is favourable in the reviewed MAV studies. St and *k* remain kinematics- and study-specific [11,34,42].	Matching Re alone does not preserve unsteady aerodynamics. Scaling requires simultaneous consideration of rotational, forward-flight, frequency, amplitude, and finite-span effects.

Notes: Re, Reynolds number; St, Strouhal number; Ro, Rossby number; *J*, advance ratio; *k*, reduced frequency; AR, aspect ratio; A/λ, nondimensional tubercle amplitude-to-wavelength ratio; $s^{+}$, nondimensional riblet spacing; $A_{g}^{+1/2}$, nondimensional groove-area parameter.

**Table 11:** Cross-domain synergy potential matrix covering five performance couplings (C-1–C-5) and one structural enabling architecture (E-1). C-5 is subdivided into C-5a and C-5b as two maturity-differentiated sub-components of a single coupling, preserving the five-coupling count (C-1–C-5). Evidence Level reflects the highest level of validation currently reported in the cited primary literature.

ID	Coupling Type	Key Quantitative Evidence	Evidence Level	Main Barrier	Priority
C-1	Physical: BWI co-origin	−5.5 dB OASPL; −20.2% shaft power; −4.4 dBA TE serration [6,19,25,119]	UAV/propeller experiments	Forward-flight validation; standardised aeroacoustic metrics	Near-term
C-2	Physical: co-located transduction	275 μW h-TENG; 379.8 μW FWMAV; 5.35 mW drone arm; 0.734 mW bistable PENG [12,13,106,107,118,120]	Component/partial UAV demos	Net mass–energy balance; durability; aeroelastic impact	Near-/mid-term
C-3	Complementarity: system-level demand reduction	Drag −7.75%; +47.99% energy gain; L/D +57.5%; $V_{\mathrm{OC}}$ 0.54 V [51,52,80,85,121,122,123,124]	Simulation + component evidence	Power gap; real-time wind estimation; no UAV-scale battery demo	Long-term
C-4	Compatibility: computation–power budget	27 μJ/inference onboard; separate offline Loihi benchmark 3–4 orders lower than Jetson Nano; RMSE 3.03° [14,15,125,126]	UAV platform experiments	Propulsion dominates power; turbulence robustness; SNN training	Mid-term
C-5a	Physical: bio-inspired nanostructure absorber layer	+93% absorption at 0° AOI (450–800 nm); +207% at 50° AOI [99,128]	PV material/absorber layer level	Scalable flexible-substrate fabrication; full-cell PCE validation; UAV operational durability	Long-term
C-5b	Physical: ultra-lightweight PV integration on UAV	Specific power 44 W/g (avg. 41 W/g); PCE 20.1%; energy-autonomous UAV flight demonstrated [101,102]	UAV platform experiments	Large-area encapsulation; ITO electrode cracking; operational stability at scale	Mid-term
E-1	Enabling architecture: structural substrate	Flight-tested morphing; separate study reporting up to +30% endurance; multifunctional morphing composite platform [9,129,130,131]	Morphing UAV prototype	Multi-objective co-design; aeroelastic co-analysis	Mid-term

**Table 12:** Qualitative cross-domain interaction matrix for representative bio-inspired UAV technologies. Entries denote the dominant effect of introducing each technology on the corresponding platform attribute: ++ substantial benefit, + benefit, ∘ negligible or not established, − penalty, −− substantial penalty, and C condition-dependent. The ratings synthesise the evidence reviewed in Section 2, Section 3, Section 4, Section 5, Section 6 and Section 7 and should not be interpreted as universal quantitative scores.

Technology	Aero./Flight	Mass	Energy	Control	Sensing	Manufacture/Maintenance
Serrated propeller geometry	++	∘	C	∘	∘	−
Leading-edge tubercles	C ^*a*^	∘	∘	∘	∘	−
Riblet surfaces	C ^*b*^	∘	C ^*b*^	∘	∘	−−
Multi-DoF morphing wing	++	−−	−	−−	∘	−−
Dynamic soaring/V-formation	++	∘	++	−−	−	∘
Structural battery composite	∘	++	+	∘	∘	−−
TENG/PENG wing skin	−	−	∘	∘	+	−
Flexible photovoltaic skin	−	−	C ^*c*^	∘	∘	−
Neuromorphic sensing/SNN control	∘	∘	+	C ^*d*^	++	−

^*a*^ Benefit is strongest near or beyond stall, whereas cruise-regime penalties can occur. ^*b*^ Benefit depends on remaining within the effective riblet-spacing regime; performance can deteriorate outside it. ^*c*^ Energy benefit depends on illumination, while laminate mass remains present under low- or zero-generation conditions. ^*d*^ Event-driven processing can reduce latency and computational energy, while training and hardware-toolchain maturity add implementation complexity.

**Table 13:** Compact comparative readiness matrix for representative bio-inspired UAV technologies. Performance values reproduce evidence already reported in Section 2, Section 3, Section 4, Section 5 and Section 6. The technology readiness level (TRL) column presents an indicative author-assigned TRL band based on the highest demonstrated integration level described in the reviewed literature; these bands were not assigned by the original studies. NR denotes not reported.

Technology	Indicative TRL	Representative Performance	Integration Complexity	Added-Mass Implication	Manufacturability/ Reliability	UAV Readiness
Bio-inspired serrated propeller	4–5	Max. 5.5 dB OASPL reduction and 20.2% mechanical-power reduction vs. DJI Phantom 3 at 50 gf thrust [6].	Medium	NR	Polyjet fabrication demonstrated; long-term reliability NR	Rotor-bench component validation
Morphing-assisted dynamic soaring	3	7.75% drag-coefficient reduction; 47.99% peak energy-gain improvement; 13.05% endurance increase per soaring cycle [122].	High	NR	Challenging; primarily simulation-based evidence	Experimental validation required
Structural-battery composite	3–4	Up to 41% projected hover-time improvement [60]; VARTM SBC energy density of 41.2 Wh kg−1 [61].	High	NR; mass-substitutive potential	VARTM demonstrated; UAV reliability NR	No open-literature UAV flight demonstration
Neuromorphic sensing/SNN control	5–6	27 μJ/inference onboard; 7.2–27.2 μJ/inference in the separate offline Loihi benchmark vs. 75.2–86.2 mJ/inference on Jetson Nano; autonomous flight demonstrated [14].	High	NR	Hardware demonstrated; training and toolchain maturity limited	Prototype flight demonstrated
Artificial electrocyte/ionic-gradient source	2–3	≈135 mV open circuit per artificial electrocyte; 165-unit series array ≈22 V open circuit [80].	High	NR	Flexible array fabricated; operational reliability limited	No sustained regulated UAV-load demonstration
Bio-inspired h-TENG harvesting	4	275 μW at 30° flap deflection, 2 Hz excitation, and 21 MΩ load resistance in the morphing-wing test [12].	Medium	NR	Morphing-wing substrate integration demonstrated; operational reliability NR	Component-level morphing-wing validation

Indicative author-assigned TRL convention: TRL 2–3 = principle or laboratory proof-of-concept; TRL 4 = component-level validation; TRL 5–6 = subsystem or prototype validation in a relevant environment. These ratings areused only for cross-domain comparison and do not constitute formal programme-specific TRL assessments.

## Data Availability

No new data were created or analysed in this study.

## Supplementary Materials

The following supporting information can be downloaded at: https://www.mdpi.com/article/10.3390/biomimetics11080596/s1, Supplementary Material S1: Literature Search Strategy and Screening Procedure.

## Abbreviations

The following abbreviations are used in this manuscript:

a-Si:H	Hydrogenated amorphous silicon
ACO	Ant colony optimisation
AI	Artificial intelligence
ANN	Artificial neural network
AHS	Artificial hair sensor
AoA	Angle of attack
ASHB	Accordion-structured hydrogel battery
BCP	Bio-inspired coupled propeller
BGB	Bi-ionic gradient battery
BIA	Bio-inspired algorithm
BM3C	Biomimetic multispectral curved compound-eye camera
BWI	Blade–wake interaction
CFD	Computational fluid dynamics
CRVP	Counter-rotating vortex pair
DoF	Degree of freedom
DS	Dynamic soaring
DVS	Dynamic vision sensor
EAP	Electroactive polymer
ESCM	Energy self-consistent model
FAV	Flapping-wing aerial vehicle
FBF	Flight-by-feel
FOA	Fruit fly optimisation
FWMAV	Flapping-wing micro air vehicle
GA	Genetic algorithm
GWO	Grey wolf optimisation
IWD	Intelligent water drops
L/D	Lift-to-drag ratio
LE	Leading edge
LEV	Leading-edge vortex
LES	Large-eddy simulation
LCO	Limit-cycle oscillation
LiPo	Lithium polymer
LITO	Low-resistance indium tin oxide
LSO	Life-cycle swarm optimisation
MALE	Medium-altitude, long-endurance
MAV	Micro air vehicle
OASPL	Overall sound pressure level
MFC	Macro-fibre composite
MPPT	Maximum power point tracking
OSC	Organic solar cell
PCE	Power conversion efficiency
PEM-FC	Proton exchange membrane fuel cell
PENG	Piezoelectric nanogenerator
PIO	Pigeon-inspired optimisation
PSO	Particle swarm optimisation
PSC	Perovskite solar cell
PV	Photovoltaic
PVDF	Polyvinylidene fluoride
RANS	Reynolds-averaged Navier–Stokes
Re	Reynolds number
RED	Reverse electrodialysis
RL	Reinforcement learning
RMSE	Root-mean-square error
SBC	Structural battery composite
SECE	Synchronous electric charge extraction
SHM	Structural health monitoring
SMA	Shape-memory alloy
SMC	Sliding-mode control
SNN	Spiking neural network
SSA	Self-sensing adhesive
SUAV	Solar-extended unmanned aerial vehicle
TE	Trailing edge
TENG	Triboelectric nanogenerator
UAV	Unmanned aerial vehicle
VARTM	Vacuum-assisted resin transfer moulding
