# Supplementary material for: A System-Level Review of Bio-Inspired Technologies for Next-Generation UAVs: From Aerodynamics to Energy Systems
Source: Biomimetics (Basel). 2026 Aug 20;11(8):596. doi: 10.3390/biomimetics11080596 (PMC13511233; doi:10.3390/biomimetics11080596)
Supplement: Supplementary file 1 [file biomimetics-11-00596-s001.zip › biomimetics-4439554-supplementary.pdf]

Supplementary Material S1

Literature Search Strategy and Screening Procedure

for "A System-Level Review of Bio-Inspired Technologies for Next-Generation UAVs: From Aerodynamics to Energy Systems"

Gyeongsu Sim, Hojin Jin, Sangyoon Woo, and Won-gyu Bae

S1. Purpose and Review Design

This supplement documents the literature-identification and screening logic used for the narrative critical engineering review. The review was not preregistered and was not originally conducted as a formal systematic review or meta-analysis. Candidate studies were initially identified iteratively through topic-based searches in Web of Science, Scopus, and Google Scholar, together with backward and forward citation tracking. In response to peer review, database-specific structured Boolean searches were developed and re-executed during revision to audit coverage and provide a reproducible record of the search logic. These revision-stage searches are therefore reported as a reproducibility and coverage audit rather than as a retrospectively claimed PRISMA-style exhaustive screening pool.

S2. Information Sources, Time Window, and Search Roles

The principal publication-eligibility window was 1 January 2015 through 30 June 2026. Earlier foundational studies were retained selectively when they established the biological mechanism, physical principle, or engineering baseline required to interpret later UAV-oriented work. Records published after 30 June 2026 were outside the review cutoff even when identified during the revision-stage audit.

Source	Role in review	Search timing	Operational note
Web of Science Core Collection	Structured revision-stage Boolean searches; citation verification	9 August 2026	2015–2026 year filter; records published after 30 June 2026 excluded during eligibility review
Scopus	Structured revision-stage Boolean searches; complementary coverage	9 August 2026	2015–2026 year filter; records published after 30 June 2026 excluded during eligibility review
Google Scholar	Targeted supplementary discovery; backward/forward citation tracking; difficult-to-index conference or foundational material	Iterative during manuscript preparation and revision	Not treated as an exact-Boolean reproducible database because ranking and hit counts are dynamic
Reference/citation chaining	Backward and forward citation tracking from high-relevance primary studies and reviews	Iterative	Used to identify foundational or directly connected studies missed by keyword indexing

S3. Eligibility Criteria

Criterion	Include	Exclude / handling
Publication type	Peer-reviewed journal articles and substantive peer-reviewed conference proceedings. Reviews were used for background, terminology, and citation chaining, but primary quantitative claims were preferentially traced to original studies.	Editorials, news items, patents, commercial pages, and non-peer-reviewed promotional material were not used as primary evidence.

Criterion	Include	Exclude / handling
Topical relevance	Study addressed a bio-inspired/biomimetic mechanism, or a directly relevant engineering comparator used to benchmark a bio-inspired concept in the manuscript.	Purely biological studies without an engineering-transfer role were excluded from the engineering evidence base.
UAV relevance	Direct UAV/MAV implementation, UAV-scale experiment, operating regime relevant to small aerial vehicles, or a clearly justified subsystem-level transfer pathway.	Technologies confined to unrelated terrestrial/marine applications without a defensible UAV transfer pathway were excluded from comparative synthesis.
Evidence content	Quantitative performance data, validated mechanistic evidence, or system-integration information sufficient to support the manuscript claim.	Studies offering only qualitative analogy without relevant engineering evidence were generally excluded from quantitative comparison.
Date	Primarily 1 January 2015–30 June 2026.	Post-cutoff studies (>30 June 2026) excluded; pre-2015 studies retained only when foundational.
Duplicate/overlapping reports	Most complete peer-reviewed version retained; complementary versions used only when they contributed non-duplicative information.	Duplicate database records and redundant versions removed during screening.

S4. Database-Specific Structured Search Strategy

The Boolean expressions below were developed for the revision-stage audit. They are intentionally domain-specific because the review spans heterogeneous aerodynamic, structural, sensing/control, storage, and harvesting literatures. A UAV term was required where the evidence base is principally aircraft- or platform-oriented (D1–D4), but it was not forced into the early-stage component searches for ionic-gradient storage and biomimetic photovoltaic materials (D5–D6), because doing so would systematically omit relevant component-level studies that are evaluated in the manuscript specifically as long-term transfer candidates. Database syntax is reported in the form actually executed on 9 August 2026. Web of Science Core Collection searches were run using the Topic field selected through the fielded-search interface; Scopus searches used TITLE-ABS-KEY. Publication-year filtering was applied through the database interfaces (2015–2026). Because the interfaces were filtered at the year level, the reported hit counts correspond to the 2015–2026 retrievals; records published after 30 June 2026 were excluded during eligibility review and were not used as review evidence.

S4.1. Web of Science Core Collection domain searches

ID	Domain	Exact Boolean string entered in Web of Science Topic field
D1	Aeroacoustics and passive drag/flow control	((UAV* OR drone* OR aircraft* OR wing* OR airfoil* OR blade* OR "unmanned aerial vehicle*" OR "uncrewed aerial vehicle*" OR "micro air vehicle*" OR "micro aerial vehicle*") AND ((owl* AND (serration* OR feather*)) OR (humpback* AND (tubercle* OR "leading edge")) OR "shark skin" OR riblet* OR "dermal denticle*" OR (biomimet* AND (aeroacoustic* OR "passive flow control"))))
D2	Aerodynamic efficiency and bio-inspired flight strategies	((UAV* OR drone* OR aircraft* OR wing* OR "unmanned aerial vehicle*" OR "uncrewed aerial vehicle*" OR "micro air vehicle*" OR "micro aerial vehicle*") AND ("morphing wing*" OR FishBAC OR "fish bone active camber" OR "dynamic soaring" OR albatross* OR "V formation" OR "formation flight" OR "flapping wing*" OR "leading edge vortex" OR "avian-inspired"))
D3	Multifunctional structures and adaptive materials	((UAV* OR drone* OR aircraft* OR wing* OR "unmanned aerial vehicle*" OR "uncrewed aerial vehicle*" OR "micro air vehicle*" OR "micro aerial vehicle*") AND ("hollow spar" OR pneumat* OR trabecular OR (gecko* AND adhesion) OR "shape memory alloy" OR piezoelectric OR "electroactive polymer" OR "multifunctional composite*" OR "structural battery*" OR "morphing skin"))

ID	Domain	Exact Boolean string entered in Web of Science Topic field
D4	Bio-inspired sensing, neuromorphic perception, and control	((UAV* OR drone* OR "unmanned aerial vehicle*" OR "uncrewed aerial vehicle*" OR "micro air vehicle*" OR "micro aerial vehicle*") AND ("event camera*" OR "event-based vision" OR neuromorphic OR "spiking neural network*" OR "compound eye*" OR "lateral line" OR "optic flow" OR "insect-inspired" OR "bio-inspired control" OR "bioinspired control"))
D5	Bio-inspired and ionic-gradient energy storage	(("artificial electrocyte*" OR "artificial electric organ*" OR "electric eel" OR "ionic gradient*" OR "hydrogel power" OR "hydrogel battery" OR "osmotic power" OR "nanofluidic osmotic" OR "salinity gradient") AND (energy OR power OR battery OR storage OR voltage OR current))
D6	Bio-inspired energy harvesting and photovoltaics	((((UAV* OR drone* OR "unmanned aerial vehicle*" OR "uncrewed aerial vehicle*" OR "micro air vehicle*" OR "micro aerial vehicle*") OR wing* OR "flexible surface*") AND ("triboelectric nanogenerator*" OR TENG OR "piezoelectric nanogenerator*" OR PENG OR "energy harvesting")) OR ((("butterfly wing" OR "photonic nanostructure*" OR (bioinspir* OR "bio-inspired" OR "bio inspired" OR biomimet* OR biomimic* OR bionic*)) AND (photovoltaic* OR "solar cell*" OR "solar energy"))))

S4.2. Scopus domain searches

ID	Domain	Exact Scopus query
D1	Aeroacoustics and passive drag/flow control	TITLE-ABS-KEY((UAV* OR drone* OR aircraft* OR wing* OR airfoil* OR blade* OR "unmanned aerial vehicle*" OR "uncrewed aerial vehicle*" OR "micro air vehicle*" OR "micro aerial vehicle*") AND ((owl* AND (serration* OR feather*)) OR (humpback* AND (tubercle* OR "leading edge")) OR "shark skin" OR riblet* OR "dermal denticle*" OR (biomimet* AND (aeroacoustic* OR "passive flow control"))))
D2	Aerodynamic efficiency and bio-inspired flight strategies	TITLE-ABS-KEY((UAV* OR drone* OR aircraft* OR wing* OR "unmanned aerial vehicle*" OR "uncrewed aerial vehicle*" OR "micro air vehicle*" OR "micro aerial vehicle*") AND ("morphing wing*" OR FishBAC OR "fish bone active camber" OR "dynamic soaring" OR albatross* OR "V formation" OR "formation flight" OR "flapping wing*" OR "leading edge vortex" OR "avian-inspired"))
D3	Multifunctional structures and adaptive materials	TITLE-ABS-KEY((UAV* OR drone* OR aircraft* OR wing* OR "unmanned aerial vehicle*" OR "uncrewed aerial vehicle*" OR "micro air vehicle*" OR "micro aerial vehicle*") AND ("hollow spar" OR pneumat* OR trabecular OR (gecko* AND adhesion) OR "shape memory alloy" OR piezoelectric OR "electroactive polymer" OR "multifunctional composite*" OR "structural battery*" OR "morphing skin"))
D4	Bio-inspired sensing, neuromorphic perception, and control	TITLE-ABS-KEY((UAV* OR drone* OR "unmanned aerial vehicle*" OR "uncrewed aerial vehicle*" OR "micro air vehicle*" OR "micro aerial vehicle*") AND ("event camera*" OR "event-based vision" OR neuromorphic OR "spiking neural network*" OR "compound eye*" OR "lateral line" OR "optic flow" OR "insect-inspired" OR "bio-inspired control" OR "bioinspired control"))
D5	Bio-inspired and ionic-gradient energy storage	TITLE-ABS-KEY(("artificial electrocyte*" OR "artificial electric organ*" OR "electric eel" OR "ionic gradient*" OR "hydrogel power" OR "hydrogel battery" OR "osmotic power" OR "nanofluidic osmotic" OR "salinity gradient") AND (energy OR power OR battery OR storage OR voltage OR current))

ID	Domain	Exact Scopus query
D6	Bio-inspired energy harvesting and photovoltaics	TITLE-ABS-KEY((((UAV* OR drone* OR "unmanned aerial vehicle*" OR "uncrewed aerial vehicle*" OR "micro air vehicle*" OR "micro aerial vehicle*") OR wing* OR "flexible surface*") AND ("triboelectric nanogenerator*" OR TENG OR "piezoelectric nanogenerator*" OR PENG OR "energy harvesting")) OR ((("butterfly wing" OR "photonic nanostructure*" OR (bioinspir* OR "bio-inspired" OR "bio inspired" OR biomimet* OR biomimic* OR bionic*)) AND (photovoltaic* OR "solar cell*" OR "solar energy"))))

S4.3. Cross-domain searches supporting Section 7

Cross-domain queries were used to test whether the functional couplings synthesized in Section 7 were directly represented in the indexed literature. These searches were not assumed to yield a large independent literature; low or zero direct-hit counts are informative where the manuscript identifies the coupling as a review-level systems synthesis rather than an already established research category.

ID	Section 7 coupling	Exact Web of Science Topic-field Boolean string	Exact Scopus query
C1	Aeroacoustics ↔ propulsion	((UAV* OR drone* OR "unmanned aerial vehicle*" OR "uncrewed aerial vehicle*" OR "micro air vehicle*" OR "micro aerial vehicle*") AND (owl* OR serration* OR aeroacoustic* OR "blade wake interaction") AND (noise OR acoustic*) AND (propulsion OR thrust OR shaft OR power OR efficiency OR drag))	TITLE-ABS-KEY((UAV* OR drone* OR "unmanned aerial vehicle*" OR "uncrewed aerial vehicle*" OR "micro air vehicle*" OR "micro aerial vehicle*") AND (owl* OR serration* OR aeroacoustic* OR "blade wake interaction") AND (noise OR acoustic*) AND (propulsion OR thrust OR shaft OR power OR efficiency OR drag))
C2	Morphing surface ↔ TENG/PENG harvesting	((UAV* OR drone* OR "unmanned aerial vehicle*" OR "uncrewed aerial vehicle*" OR "micro air vehicle*" OR "micro aerial vehicle*") AND (morphing OR "flexible wing" OR "wing skin" OR deformation) AND (triboelectric OR piezoelectric OR TENG OR PENG OR "energy harvesting"))	TITLE-ABS-KEY((UAV* OR drone* OR "unmanned aerial vehicle*" OR "uncrewed aerial vehicle*" OR "micro air vehicle*" OR "micro aerial vehicle*") AND (morphing OR "flexible wing" OR "wing skin" OR deformation) AND (triboelectric OR piezoelectric OR TENG OR PENG OR "energy harvesting"))
C3	Flight strategy ↔ low-power/ionic storage	((UAV* OR drone* OR "unmanned aerial vehicle*" OR "uncrewed aerial vehicle*" OR "micro air vehicle*" OR "micro aerial vehicle*") AND ("dynamic soaring" OR "formation flight" OR "V formation") AND ("energy storage" OR battery OR "ionic gradient" OR electrocyte OR avionics OR "power budget"))	TITLE-ABS-KEY((UAV* OR drone* OR "unmanned aerial vehicle*" OR "uncrewed aerial vehicle*" OR "micro air vehicle*" OR "micro aerial vehicle*") AND ("dynamic soaring" OR "formation flight" OR "V formation") AND ("energy storage" OR battery OR "ionic gradient" OR electrocyte OR avionics OR "power budget"))
C4	Neuromorphic sensing/control ↔ power budget	((UAV* OR drone* OR "unmanned aerial vehicle*" OR "uncrewed aerial vehicle*" OR "micro air vehicle*" OR "micro aerial vehicle*") AND (neuromorphic OR "spiking neural network*" OR "event-based vision" OR "event camera*") AND (energy OR power OR consumption OR efficiency OR "power budget"))	TITLE-ABS-KEY((UAV* OR drone* OR "unmanned aerial vehicle*" OR "uncrewed aerial vehicle*" OR "micro air vehicle*" OR "micro aerial vehicle*") AND (neuromorphic OR "spiking neural network*" OR "event-based vision" OR "event camera*") AND (energy OR power OR consumption OR efficiency OR "power budget"))
C5	Wing/surface ↔ bio-inspired photovoltaics	((UAV* OR drone* OR "unmanned aerial vehicle*" OR "uncrewed aerial vehicle*" OR "micro air vehicle*" OR "micro aerial vehicle*") AND (wing OR surface OR skin) AND (photovoltaic* OR solar OR "photonic nanostructure*" OR "butterfly wing") AND (bioinspir* OR "bio-inspired" OR "bio inspired" OR biomimet* OR biomimic* OR bionic*))	TITLE-ABS-KEY((UAV* OR drone* OR "unmanned aerial vehicle*" OR "uncrewed aerial vehicle*" OR "micro air vehicle*" OR "micro aerial vehicle*") AND (wing OR surface OR skin) AND (photovoltaic* OR solar OR "photonic nanostructure*" OR "butterfly wing") AND (bioinspir* OR "bio-inspired" OR "bio inspired" OR biomimet* OR biomimic* OR bionic*))
E1	Multifunctional morphing substrate enabling energy integration	((UAV* OR drone* OR "unmanned aerial vehicle*" OR "uncrewed aerial vehicle*" OR "micro air vehicle*" OR "micro aerial vehicle*") AND (morphing OR multifunctional OR composite OR "wing skin") AND ("energy harvesting" OR photovoltaic* OR TENG OR PENG OR "structural battery*") AND (integration OR substrate OR multifunctional))	TITLE-ABS-KEY((UAV* OR drone* OR "unmanned aerial vehicle*" OR "uncrewed aerial vehicle*" OR "micro air vehicle*" OR "micro aerial vehicle*") AND (morphing OR multifunctional OR composite OR "wing skin") AND ("energy harvesting" OR photovoltaic* OR TENG OR PENG OR "structural battery*") AND (integration OR substrate OR multifunctional))

S4.4. Google Scholar and citation chaining

Google Scholar was used as a supplementary discovery tool rather than as an equivalent structured database. Targeted combinations of the UAV terms and the mechanism terms listed above were used to locate hard-to-index conference papers, recent online-first studies, and citation networks. Because Google Scholar query interpretation, ranking, and reported hit counts can change over time, its results were not treated as a stable exact-Boolean screening denominator. Backward and forward citation tracking was then applied to high-relevance primary studies and major reviews.

S5. Screening and Selection Procedure

Screening during manuscript preparation was performed iteratively at the level appropriate to a narrative critical engineering review. Titles and abstracts were assessed for topical and UAV relevance, and full texts were checked when a study appeared capable of supporting a quantitative statement, mechanism-level inference, or system-integration argument. During revision, the structured database outputs were used as a reproducibility and coverage audit rather than retrospectively converted into an exhaustive systematic-review screening pool. Studies retained in the manuscript satisfied the eligibility criteria in Table S2 and contributed non-redundant evidence to at least one technical domain or cross-domain synthesis. Where a recent review identified a potentially relevant study, the original primary paper was sought and used for quantitative claims whenever accessible.

- Step 1: Remove duplicate records across databases and duplicate versions of the same study.
- Step 2: Title/abstract relevance check against the six technical domains and the UAV transfer criterion.
- Step 3: Full-text verification of quantitative values, experimental/simulation conditions, and the biological-to-engineering rationale.
- Step 4: Prefer primary studies for performance claims; use reviews mainly for synthesis, context, or citation chaining.
- Step 5: Record whether evidence is component-level, subsystem-level, UAV-platform experimental, or conceptual/system-synthesis evidence.
- Step 6: Retain earlier-than-2015 studies only when foundational to a mechanism or benchmark used in the review.

S6. Search Execution Log

Result counts were recorded immediately after each query was executed on 9 August 2026. This log documents the revision-stage reproducibility and coverage audit rather than a retrospective PRISMA screening denominator. Revision-stage hits were therefore not exhaustively re-screened as a new systematic-review pool. Coverage notes summarise whether the retrieved records represented the manuscript technical domain or provided direct evidence for the Section 7 cross-domain coupling. Reported hit counts are the observed database results after the 2015–2026 year filter and were not back-calculated or estimated.

Query	Scope	Execution date	Hits	Coverage-audit note
D1-WOS	Aeroacoustics and passive drag/flow control — Web of Science	9 Aug 2026	600	Retrieved owl-serration, tubercle, riblet, and passive-flow-control records, including Reynolds-sensitive tubercle studies.
D1-SC	Aeroacoustics and passive drag/flow control — Scopus	9 Aug 2026	745	Complementary coverage of owl-inspired aeroacoustics, tubercled leading edges, riblets, and passive-flow-control studies.
D2-WOS	Aerodynamic efficiency and bio-inspired flight strategies — Web of Science	9 Aug 2026	4,717	Retrieved morphing-wing, FishBAC, dynamic-soaring, formation-flight, and flapping-wing records relevant to Section 3.
D2-SC	Aerodynamic efficiency and bio-inspired flight strategies — Scopus	9 Aug 2026	6,467	Complementary coverage of morphing wings, FishBAC, dynamic soaring, formation flight, and flapping-wing strategies.
D3-WOS	Multifunctional structures and adaptive materials — Web of Science	9 Aug 2026	1,891	Retrieved SMA/piezoelectric actuation, morphing skins, multifunctional composites, and structural-battery records.
D3-SC	Multifunctional structures and adaptive materials — Scopus	9 Aug 2026	3,538	Complementary coverage of adaptive actuation, multifunctional composites, structural batteries, and morphing skins.
D4-WOS	Bio-inspired sensing, neuromorphic perception, and control — Web of Science	9 Aug 2026	424	Retrieved event-camera, spiking-network, and neuromorphic UAV records, including low-power flight-control studies.

Query	Scope	Execution date	Hits	Coverage-audit note
D4-SC	Bio-inspired sensing, neuromorphic perception, and control — Scopus	9 Aug 2026	668	Complementary coverage of event-based vision, SNN control, neuromorphic processing, and low-power UAV computation.
D5-WOS	Bio-inspired and ionic-gradient energy storage — Web of Science	9 Aug 2026	2,465	Retrieved electric-eel/electrocyte, ionic-gradient, hydrogel, salinity-gradient, and osmotic-energy records.
D5-SC	Bio-inspired and ionic-gradient energy storage — Scopus	9 Aug 2026	2,804	Complementary coverage of electric-eel biomimetics, hydrogel/ionic systems, and nanofluidic osmotic-energy conversion.
D6-WOS	Bio-inspired energy harvesting and photovoltaics — Web of Science	9 Aug 2026	2,495	Retrieved TENG/PENG harvesting and biomimetic photovoltaic records, including morphing-wing and butterfly-inspired concepts.
D6-SC	Bio-inspired energy harvesting and photovoltaics — Scopus	9 Aug 2026	3,305	Complementary coverage of triboelectric/piezoelectric harvesting and bio-inspired photovoltaic/photonic concepts.
C1-WOS	Aeroacoustics ↔ propulsion — Web of Science	9 Aug 2026	91	Direct cross-domain evidence identified: retrieved studies jointly evaluated UAV/rotor aeroacoustics and aerodynamic or propulsive performance.
C1-SC	Aeroacoustics ↔ propulsion — Scopus	9 Aug 2026	169	Direct cross-domain evidence identified across serrated and bio-inspired propeller studies reporting noise and thrust/efficiency outcomes.
C2-WOS	Morphing surface ↔ TENG/PENG harvesting — Web of Science	9 Aug 2026	44	Direct cross-domain evidence identified: morphing/flexible-wing studies coupled deformation with piezoelectric or other energy harvesting.
C2-SC	Morphing surface ↔ TENG/PENG harvesting — Scopus	9 Aug 2026	78	Direct cross-domain evidence identified, including morphing-wing piezoelectric, triboelectric, and solar-energy-harvesting architectures.
C3-WOS	Flight strategy ↔ low-power/ionic storage — Web of Science	9 Aug 2026	10	Direct evidence limited: flight-strategy records addressed energy use or storage, but not direct ionic-gradient/electrocyte integration.
C3-SC	Flight strategy ↔ low-power/ionic storage — Scopus	9 Aug 2026	17	Direct evidence limited: dynamic-soaring/formation studies addressed energy or battery considerations, but not ionic-gradient/electrocyte coupling.
C4-WOS	Neuromorphic sensing/control ↔ power budget — Web of Science	9 Aug 2026	144	Direct cross-domain evidence identified: neuromorphic/event-based UAV studies explicitly addressed low-power or energy-efficient computation.
C4-SC	Neuromorphic sensing/control ↔ power budget — Scopus	9 Aug 2026	237	Direct cross-domain evidence identified across event-camera and SNN UAV studies reporting power or energy-efficiency advantages.
C5-WOS	Wing/surface ↔ bio-inspired photovoltaics — Web of Science	9 Aug 2026	14	Direct evidence limited: bio-inspired/solar wing records were retrieved, but direct photonic-biomimetic PV surface integration was sparse.
C5-SC	Wing/surface ↔ bio-inspired photovoltaics — Scopus	9 Aug 2026	32	Direct evidence limited: related solar-UAV and bio-inspired wing studies were retrieved, while direct biomimetic-photonic PV integration remained sparse.
E1-WOS	Multifunctional morphing substrate enabling energy integration — Web of Science	9 Aug 2026	19	Direct enabling evidence identified through structural batteries, multifunctional wings/skins, and integrated energy-harvesting concepts.
E1-SC	Multifunctional morphing substrate enabling energy integration — Scopus	9 Aug 2026	28	Direct enabling evidence identified, although retrieved integrations were heterogeneous and mainly subsystem- or prototype-level.

S7. Relationship Between Search Domains and Manuscript Sections

Search ID	Manuscript location	Coverage
D1	Section 2	Owl-feather serrations; humpback-whale tubercles; shark-skin riblets; passive aeroacoustic/drag-control mechanisms
D2	Section 3	Morphing wings; dynamic soaring; V-formation; flapping-wing/leading-edge-vortex strategies
D3	Section 4.1	Bio-inspired structural materials, multifunctional composites, morphing skins, adhesion and adaptive actuation
D4	Section 4.2 and control discussion	Compound-eye/lateral-line sensing; event cameras; neuromorphic and spiking-network perception/control
D5	Section 5	Ionic-gradient/electric-eel-inspired storage; hydrogel/electrocyte architectures; nanofluidic osmotic concepts
D6	Section 6	Triboelectric/piezoelectric harvesting; bio-structured photovoltaic and photonic surfaces
C1	Section 7.1	Aeroacoustics ↔ propulsion
C2	Section 7.2	Morphing surface ↔ TENG/PENG harvesting
C3	Section 7.3	Flight strategy ↔ low-power/ionic storage
C4	Section 7.4	Neuromorphic sensing/control ↔ power budget
C5	Section 7.5	Wing/surface ↔ bio-inspired photovoltaics
E1	Section 7.6	Multifunctional morphing substrate enabling integrated energy functions

S8. Reproducibility Boundaries and Limitations

The structured searches documented here improve transparency but do not convert the manuscript into a formal systematic review. The six technical domains use different terminologies, indexing conventions, and evidence types, and a single umbrella query would generate a large heterogeneous pool with poor specificity. The domain-specific audit therefore prioritises reproducibility of search logic while preserving the critical engineering-review design. In addition, Google Scholar ranking is dynamic, conference-paper indexing is uneven, and some foundational studies predate the principal time window. These limitations are addressed by citation chaining, selective retention of foundational studies, and explicit separation between direct evidence and review-level system synthesis.
